# Estimation of food portion sizes in women of childbearing age and young children in Ouagadougou (Burkina Faso) using a food photography atlas and salted replicas: Comparison with weighed records

**DOI:** 10.1371/journal.pone.0291375

**Published:** 2023-09-18

**Authors:** Stéphanie Zoungrana, Jérome W. Somé, Yves Martin-Prével, Hermann B. Lanou, Séni Kouanda, Claire Mouquet-Rivier

**Affiliations:** 1 Qualisud, Institut de Recherche pour le Développement, L’Institut Agro, Cirad, Avignon Université, Université de la Réunion, Université de Montpellier, Montpellier, France; 2 Research Institute of Health Sciences, Ouagadougou, Burkina Faso; 3 Moisa, Institut de Recherche pour le Développement, L’Institut Agro, Cirad, Ciheam-Iamm, Université Montpellier, Montpellier, France; Wroclaw University of Environmental and Life Sciences: Uniwersytet Przyrodniczy we Wroclawiu, POLAND

## Abstract

Quantitative assessment of foods consumed when using 24-hour dietary recall requires accurate tools to estimate portion sizes. Therefore, we developed a food portion photography atlas with age-appropriate portion sizes for 11 foods frequently consumed by young children (sizes for 6-11-month- and for 12-23-month-old children) and women of childbearing age in Ouagadougou, Burkina Faso capital. We then compared the accuracy and precision of portion estimation with this atlas and with salted replicas relative to weighed records (the reference). After weighing, we randomly assigned food portions to 67 women and their children. The next day, women estimated the served portions and leftovers by recall using the atlas and then salted replicas (n = 1156 measurements, ranging from 19 to 113 for each food). For most food types, the portions estimated with the atlas and salted replicas were positively correlated and showed good concordance with the weighed records. However, accuracy and precision varied in function of the estimation method, food type, and age group. The mean crude differences ranged from -28 to +12g (with errors in absolute values from 24 to 69%) for children, and from -32 to +44g (errors from 17 to 56%) for women. The atlas-based method showed the lowest Lin’s concordances (coefficients of 0.1 to 0.2) for the leafy vegetable dish, meat, and fish in 12-23-month-old children. Bland-Altman plots indicated that the salted replicas allowed estimating the consumed portions with fewer errors than the photographic atlas (56 to 91% vs 46 to 79% between the limits of ±50%). Our study highlights that mothers have difficulties in perceiving the quantities of food consumed by their children. Our findings also indicate that the food atlas could be used in food consumption surveys when salted replicas are not available for all food types.

## Introduction

Undernutrition persists in low-income countries, such as Burkina Faso [1, 2]. Moreover, the nutritional transition in these populations is promoting overweight, obesity and diet-related chronic diseases [1, 3–5]. Therefore, reliable data on the risks of nutrient deficiencies and excesses in populations are needed to better guide nutritional policies and evaluate the effects of nutrition programs [6]. Several methods can be used to measure food intakes at the individual level. Weighed records (WR) are considered the gold standard [7, 8], but this method is difficult to implement in large-scale studies because it is expensive and time-consuming for responders. A common alternative is the 24-hour recall method in which the responder gives a quantitative estimate of all foods and beverages consumed in the previous 24 hours [7–9]. As this approach is cheaper and less time-consuming, it can be used in large samples, and even in low-literacy populations [7, 8]. However, it requires the use of tools to estimate the size of the consumed food portions, A food portion size is the amount of a food item served, consumed or left within an eating occasion [10]. The food portion size determines not only the amount of energy consumed, but also the intake of macro- and micro- nutrients, and therefore has an important nutritional impact. Its estimation is one of the main sources of error. Standardized tools to measure food portion sizes adapted to Burkina Faso are lacking. Moreover, local eating practices (e.g. eating from a common bowl, eating by hand, and sharing food) increase the difficulty.

The challenge is even higher when measuring the food portion eaten by <2-year-old children in the previous day for several reasons. Particularly, infants and young children cannot answer and the interview must be carried out with a caregiver. When feeding children, significant food losses can occur (e.g. on the floor, clothes, bibs, during meals). A national food consumption survey in the United Kingdom showed that on average, <4-year-old children do not eat ~10%, and up to 40% of the served portions, depending on the food [11]. These losses and the leftovers must be estimated, and inaccuracies in their quantification negatively affect the estimation of the actually consumed portions.

Several 2D, 3D, digital, and non-digital methods and tools are available to estimate food portion sizes. Direct weighing of food leftovers or salted replicas (SR) and food photography (FP) atlases are two commonly used methods. SRs are real food portions saturated with salt to ensure their preservation at room temperature for few days; they constitute a realistic way for the responder to visualize the consumed amount in retrospective food consumption surveys [9, 10]. However, their use is constraining because SRs require the preparation and transportation of large quantities of food and cannot be used when testing large numbers of food types [10].

FP atlases are composed of series of scaled-down photographs of a food or mixed dish. Food items are typically shown on a plate. An object of known size is shown next to the plate to provide a reference for size. The portion sizes depicted in the images for a photographic series correspond to different portion sizes from very small to very large [10]. The use of FP atlases is well documented in the literature. They allow concomitantly presenting several foods with a choice of portion sizes for each dish [9]. In Africa, very few FP atlases have been developed and validated. For instance, studies in South Africa, Malawi, Tunisia, and Cameroon, described the development and validation of FP atlases of 5 to 20 food types for different population groups (from 8 to 89 years of age) [12–15]. In Burkina Faso, Huybregts et al. developed and validated a FP atlas with portion sizes for eight food items commonly consumed by rural women of childbearing age [16]. There are also FP atlases specifically developed for <18-year-old children [17–21], but atlases for <2-year-old children are very rare. In Ethiopia, an FP catalog was recently developed to estimate food portion sizes in 6-13-month-old children [22]. This catalog, which contains portion sizes of porridge with five different consistencies, was validated in a study that included 548 parents.

Therefore, the first objective of this study was to develop a FP atlas of 11 food items with portion sizes appropriate for 6-11-month and 12-23-month-old children, and also for women of childbearing age living in the city of Ouagadougou. The second objective was to assess and compare the food portion sizes that women and their children had consumed the day before and that women estimated using the FP atlas and SRs. The accuracy and precision of these two estimation methods were evaluated against the reference method (WR) to select the most appropriate method for future 24-hour recall surveys.

## Material and methods

### Schematic overview of the study program

**Fig 1:**
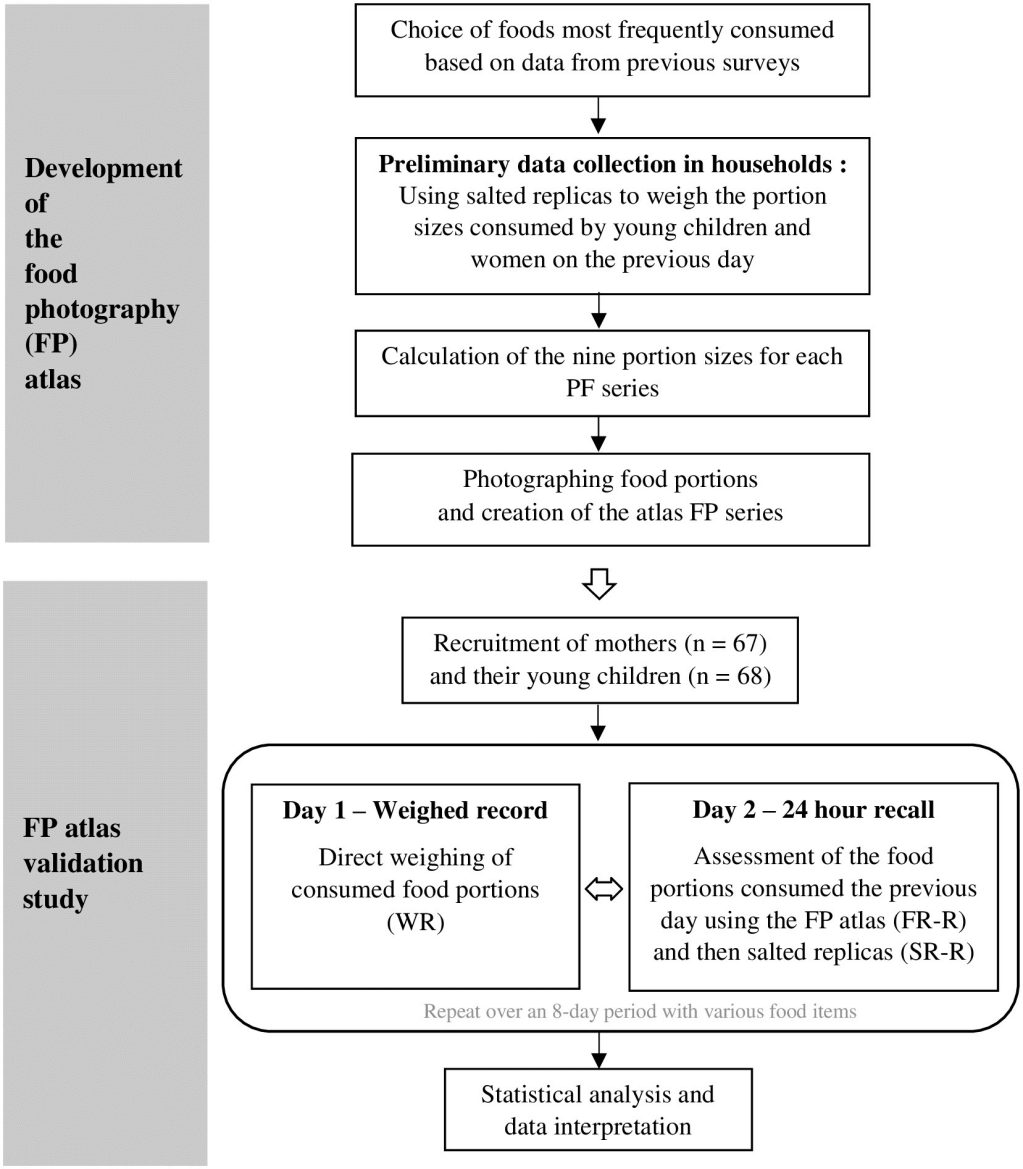
Flow diagram presenting a schematic overview of the study program.

The study involved two main stages: first, the FP atlas was developed, and then submitted to a validation study, as shown on the flow diagram provided in Fig 1. Methodological details are provided for each of the two stages of the study, in the paragraphs thereafter. The detailed protocol of the study was approved by an ethics committee in Burkina Faso, the Ethics Committee of the Research Institute in Health Science (N° 29-2019/CEIRES). Additional information regarding the ethical, cultural, and scientific considerations specific to inclusivity in global research is included in the Supporting Information (S1 Checklist).

### Development of the food photography atlas

We developed the FP atlas for cooked food items or complete dishes according to the guidelines proposed by Nelson and Haraldsdóttir [23, 24].

#### Food choice

We selected eleven traditional food items/dishes from the database of a previous food consumption survey carried out in Ouagadougou (INSTAPA project, unpublished data, 2009): five cereal-based staple food items (stiff corn porridge, fermented millet porridge, cowpea with rice, rice, and spaghetti), one leafy vegetable dish, three sauces (groundnut paste sauce, vegetable sauce, dried okra sauce), and two standalone food items (pieces of meat and fish). We chose these food items on the basis of the following criteria: their high consumption frequency or the difficulty of using other methods to estimate the consumed portions. As meat and fish are rarely consumed by 6-11-month-old children, we did not include them in the atlas for this age group.

#### Portion size determination—Data collection

To roughly estimate the portion sizes to include in the FP atlas, we determined the most frequent portion sizes for the groups targeted by this study (6-11-month and 12-23-month-old children and women of childbearing age) through a preliminary 24-hour portion recall survey using SRs. The aim was to quantify portions in at least ten people for each food item and for each group. We carried out this preliminary survey in 130 households visited at random and that included women of childbearing age, 6-11-month children, or 12-23-month-old children in January 2020. Half of participants in this survey lived in a central area of Ouagadougou (with urban infrastructure i.e. a regularly delimited area with water and electricity supply networks and paved roads) and half in a peripheral area (still without urban infrastructure i.e. area without water and electricity supply networks and paved roads). These areas are formally defined by the territorial authorities. After obtaining their informed consent, we asked participants to estimate, using SRs, the portion of at least one of the eleven selected food items they had consumed the previous day. Specifically, we weighed the amount of SR they thought corresponded to what they had eaten the day before. We collected 628 portion estimates: 151 for 6-11-month-old children (n = 10 to 20 for each food item, except for cowpea with rice where n = 7), 239 for 12-17-month-old children (n = 13 to 31 for each food type), and 238 for women (n = 11 to 36 for each food type).

#### Portion size determination—Calculation method

The objective was to present in the FP atlas nine different portions (named A, B, C, D, E, F, G, H, I), from the smallest to the largest, for each food item and each age group. We took photographs of the four portion sizes B, D, F and H (portions A, C, E, G and I were only virtual). The calculation method we used to determine the portion sizes was adapted from those used in similar studies [14, 16, 25]. We calculated the mean SR portion size and standard deviations (SD) for each food item and each age group, and then we defined the portion sizes as follows:

- Portion E = mean
- Portion D = mean—0.5 SD; portion F = mean + 0.5 SD
- Portion C = (B+D)/2; portion G = (F+H)/2
- Portion B = mean—1.5 SD; portion H = mean + 1.5 SD
- Portion A = B/2; portion I = H + ½H.

For some food items, we adjusted some portions (B or H, n = 9 of the 279 calculated portions) if they were considered too large or too small compared with the mean portion.

#### FP atlas preparation

**Fig 2:**
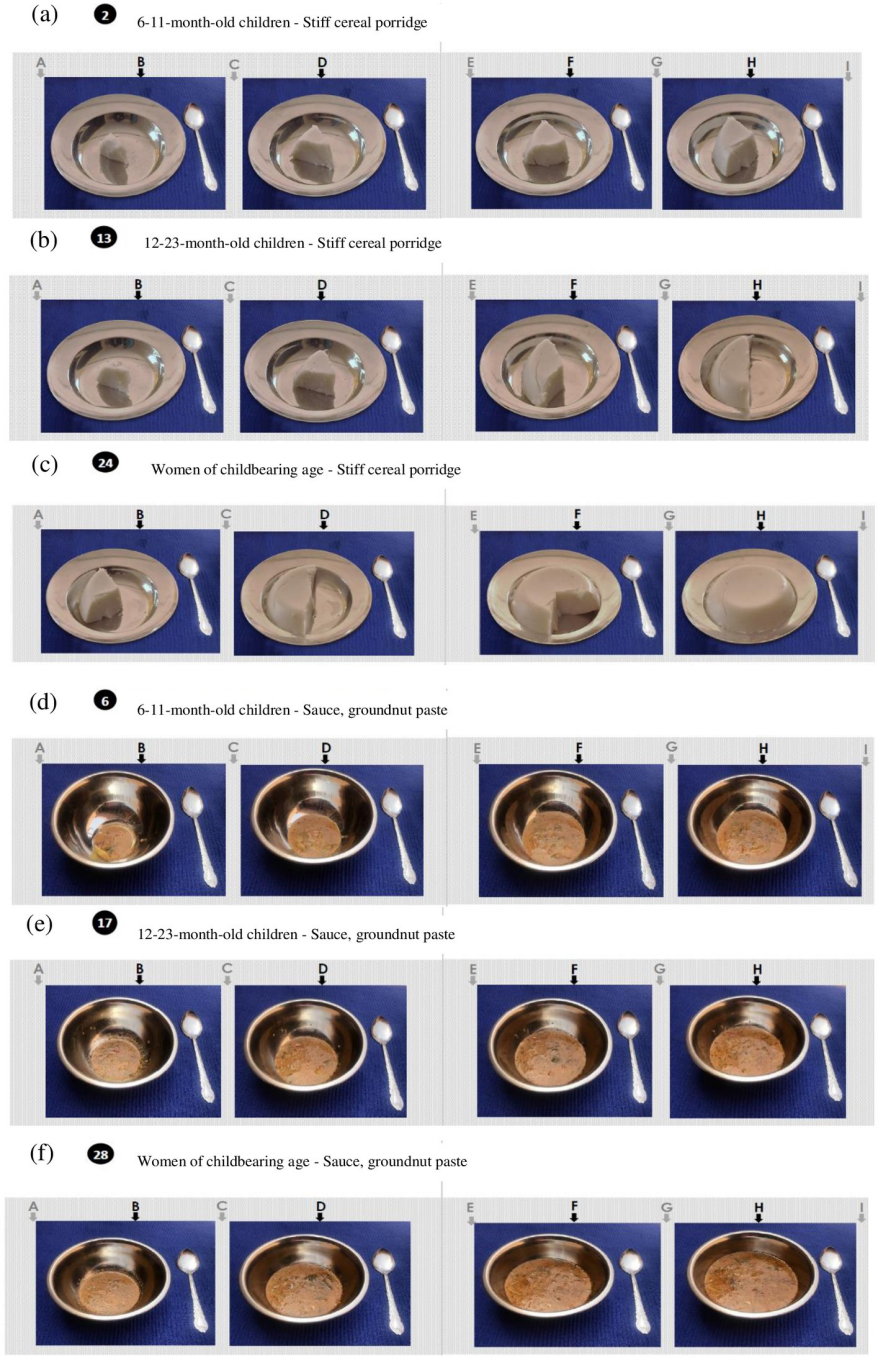
Examples from the food photography atlas. (a) Stiff cereal porridge portions for 6-11-month-old children; (b) Stiff cereal porridge portions for 12-23-month-old children; (c) Stiff cereal porridge portions for women; (d) Groundnut paste sauce portions for 6-11-month-old children; (e) Groundnut paste sauce portions for 12-23-month-old children; (f) Groundnut paste sauce portions for women.

We cooked each food item/dish and then we weighed portions B, D, F, and H according to the calculations described above. We then put them in plates and bowls frequently used by the population for taking photographs with a 12-megapixel camera. We arranged all color photographs (7x9 cm) on A3 size sheets to concomitantly visualize the nine portions (four photographs with virtual portions in between) from left to right (Fig 2; the complete FP atlas with portion weights is available in S1 File).

### Validation study design

#### Study sample

The objective of the validation study was to assess whether participants could estimate the food portions they consumed on the previous day using the FP atlas and SRs, and to measure the accuracy and precision of these tools. In these validation studies, the same participant can make multiple measurements, according to the guidelines by Nelson and Haraldsdottir [23]. To obtain at least 30 measurements for each food item/dish and age group, we enrolled 60 women and their children (n = 30 6-11-month and n = 30 12-23-month-old children). We included another seven children and seven women to compensate for potential dropouts. To recruit the mother-child pairs, we selected a recruitment zone located in a peripheral district of Ouagadougou comprising areas with and without urban infrastructure. Then, we conducted a door-to-door enumeration of households looking for our target population. Eligibility criteria were that participants should reside in the study area, mothers or guardians should be 15–49 years old and have a child aged 6–23 months, and they should be available for at least 8 consecutive days from the study start date. After explaining the study purpose and implementation to the participants, and giving them an information note, we asked them to sign an informed consent form. We recorded the women’s age and education level. To make sure that all participants consumed all the food items/dishes present in the FP atlas at least once and to provide a complete meal every day, we assigned the eleven selected food items/dishes to seven meals that comprised one to three of them. Participants consumed stiff corn porridge, rice, meat and fish at two occasions because they were associated with different sauces to constitute meals (meal 1: spaghetti and fish; meal 2: stiff corn porridge, dry okra sauce, and meat; meal 3: rice, groundnut paste sauce, and meat; meal 4: fermented millet porridge; meal 5: cowpea with rice; meal 6: stiff corn porridge and leafy vegetable dish; meal 7: rice, vegetable sauce, and fish). Thus, we expected to obtain 363 measurements for 6-11-month-old children, 525 for 12-23-month-old children, and 1005 for women. A measurement corresponded to one portion of one weighed and consumed food estimated by 24-hour recall using the FP atlas (FP-R) and SRs (SR-R).

#### Study description

We made a contract with one household located in the study area that agreed to become the study site for three periods of eight consecutive days/each. During each 8-day period, roughly twenty women and their children were asked each day to eat a meal and, the next day, to estimate the portions they or their children had consumed the day before. Portion sizes were randomly assigned to participants; medium-sized portions (C, D, E, F and G) were proposed more frequently (four to five) than very small and very large portions (A, B and H, I). For any given food item, on day one, the portions were weighed in plates or bowls identical to those used in the atlas, using a kitchen scale with a precision of 1g, and then served and consumed by participants. Mothers fed their children before consuming their own portion. At the end of the meal, leftovers were also weighed, to determine by difference the actual portions consumed by each participant (WR i.e. portion served minus leftover). Finally, part of the day food was salted to be used as SRs on day two. On day two, participants came back to the study site and they first estimated the portions they were served the previous day and their leftovers, using the FP atlas (FP-R) and then using the SR (SR-R: each mother put in a plate the part of the SR that corresponded to the food consumed the day before). Mothers started by estimating the portions consumed by their children and then their own portions. After the 24-hour recall, they consumed the meal for that day.

### Data collection and analysis

We collected data on digital tablets using KoboCollect^™^-generated Excel forms to allow their quick and correct transfer to the KoboToolbox^™^ platform. We performed all statistical analyses with the R software, version 4.0.5. We calculated the means and standard deviations (SD) of the amounts of food consumed. As data most often did not follow the normal distribution, we used the Spearman’s test to assess correlations between the estimation methods and the reference method. We calculated the mean of the differences between estimated and weighed portions and the means of the absolute values of these differences (in grams) and corresponding relative errors (based on the reference values). We used Lin’s coefficients to assess the agreement between each estimation method and the reference method [26, 27]. This coefficient quantifies the linear relationship between methods; it ranges from -1 to +1 (-1, 0, and +1 indicate perfect discordance, zero concordance, and perfect concordance, respectively). For the interpretation of the coefficient values, we used the limits defined by Landis and Koch: <0 "Poor", 0–0.2 "Slight", 0.21–0.4 "Fair", 0.41–0.60 "Moderate", 0.61–0.80 "Substantial", and 0.81–1.00 "Almost perfect" [28]. To evaluate the agreement between estimations and reference method, we generated the Bland and Altman’s relative difference plots for each food item [29, 30]; we set the upper and lower limits at ± 50% and then calculated the number of observations within this range.

We also expressed differences as percentages of the recommended intakes: the estimated average requirement of energy and the recommended nutrient intakes of protein, iron, zinc, vitamin A and vitamin B9 [31, 32]. For this, we calculated the nutritional values of each food using the West African Food Composition Table [33].

## Results

### Sample characteristics

**Fig 3:**
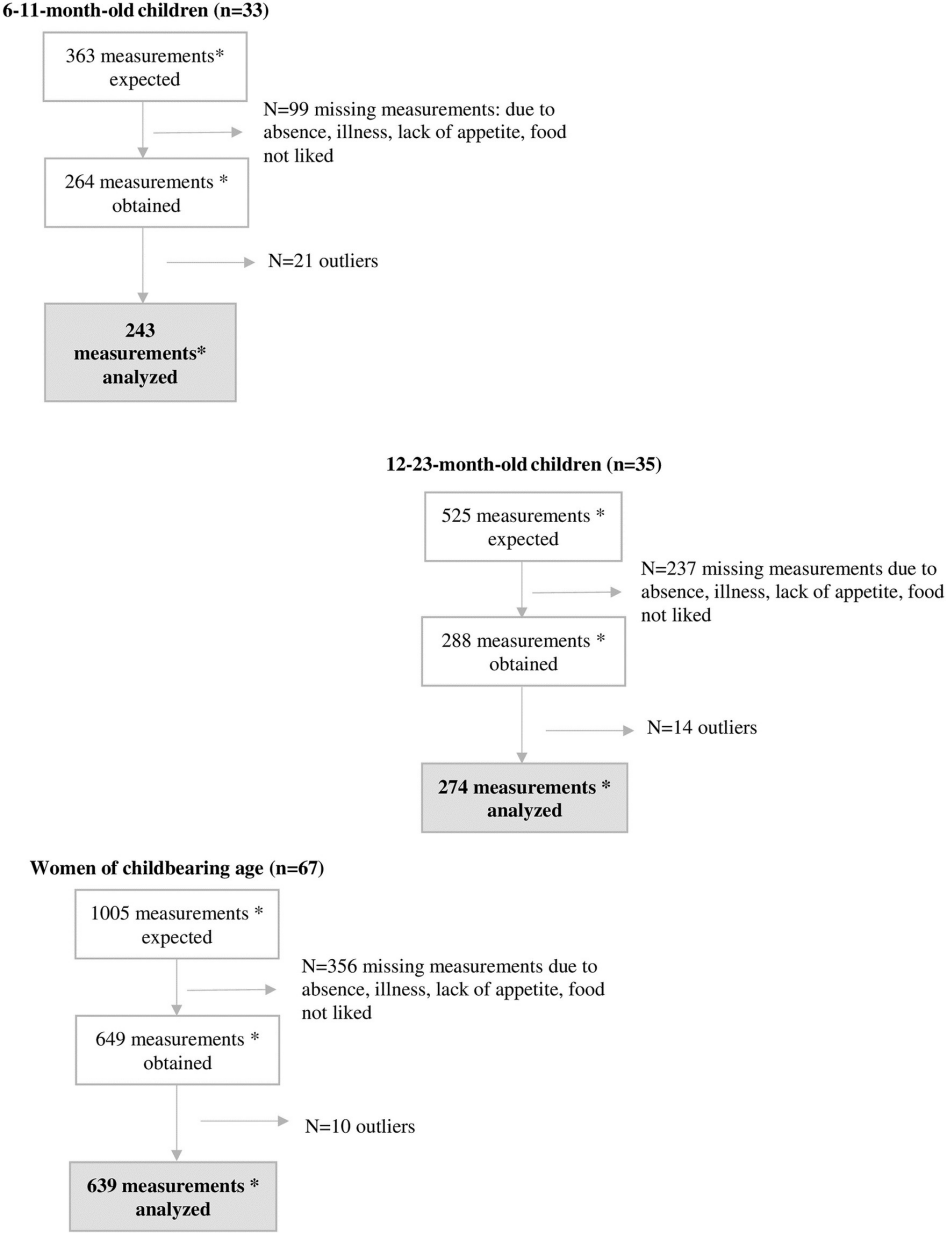
Flowchart of participants and data inclusion for the validation study. For 6-11-month-old children, 9 food items were offered in 7 consecutive days (4 food items were proposed in two different days and 5 food items only once). For 12-23-month-old children and for women, 11 food items were offered in 7 consecutive days (4 food items were proposed in two different days and 7 food items only once). *measurement = 1 weighed record of the actually consumed quantity + 1 estimation of the consumed quantity with the FP + 1 estimation with the SR.

The study sample consisted of 67 caregivers and 68 children (one mother had twins): 33 children were in the 6–11 months group and 35 in the 12–23 months group (Table 1). The caregivers’ mean age was 28 years (18–49 years). Most caregivers were the children’s mothers, except two (for the sake of simplicity, we will call all of them ‘mothers’ or women throughout the article): 52% had attended school and 17% lived in an area with urban infrastructure. Overall, 1156 of the expected 1893 measurements were obtained (692 were missing due to participants’ absence, lack of appetite, refusal of some foods, incomplete measurements, and 45 were outliers) (Fig 3). Therefore, we could analyze 243 measurements for 6-11-month-old children (n = 19 to 36 per food item), 274 for 12-23-month-old children (n = 18 to 46), and 639 for women (n = 24 to 113).

**Table 1:** Sample composition and main characteristics^a^.

	Children aged 6–11 months	Children aged 12–23 months	Women of childbearing age
**Number of participants**	33	35	67^b^
**Education level (mothers)**			
Schooled—n (%)	20 (61)	16 (46)	35 (52)
Unschooled—n (%)	13 (39)	19 (54)	32 (48)
**Residence**			
Area with urban infrastructure- n (%)	7 (21)	10 (29)	17 (25)
Area without urban infrastructure—n (%)	26 (79)	25 (71)	50 (75)
**Mothers’ age**			
Mean age (years) (min—max)	26.3 (18–40)	30.1 (20–49)	28.0 (18–49)

^a^Number of mother-child pairs = 67

^b^One woman was the mother of twins in the 12–23 months group

### For 6–11 month-old children

For most food items, the portion estimates obtained with the two estimation methods (FP-R and SR-R) were strongly and positively correlated with the WRs (reference method) (r = 0.6–0.9, p <0.05), but for the stiff corn porridge and spaghetti portions estimated by FP-R (r = 0.4, p <0.05 and r = 0.3, p >0.05, respectively) and for the dry okra sauce portion estimated by SR-R (r = 0.4, p <0.05) (Table 2). The mean crude differences between the amounts estimated by FP-R and those actually consumed (WR) ranged from -16 to +1 g, but the compensation between underestimation and overestimation decreased the mean difference. The mean absolute differences were higher (from 6 to 24 g) and corresponded to relative estimation errors that ranged from 25 to 45% (Table 2). The mean crude differences between the amounts estimated by SR-R and WR ranged from -1 to +12 g; the mean absolute differences varied between 6 and 18 g and the mean relative errors between 24 and 69%. In this age group, the SR-R tended to overestimate the portion size, compared with the WR. Conversely, the FP-R tended to underestimate the portion size. The mean error obtained by SR-R for spaghetti was much smaller than the one obtained by FP-R, whereas those obtained for rice and cowpea with rice were much larger.

**Table 2:** Comparison between the actual quantity consumed (weighed record -WR) and the consumed portion sizes estimated by 24-hour recall using the food photography atlas (FP-R) or salted replicas (SR-R) for 6-11-month-old children.

	6-11-month-old children																	
	n	WR^b^ (g) Mean (SD)	FP-R (g) Mean (SD)	SR-R (g) Mean (SD)	Lin’s concordance coefficient^c^		Spearman’s correlation *r*		Mean difference^d^ (g)		Mean |difference|^e^ (g)		|Mean error|^f^ (%)		Bland Altman Analysis^g^			
															Bias (%)		Measurements between -50% and +50% limits (%)	
					FP-R	SR-R	FP-R	SR-R	FP-R	SR-R	FP-R	SR-R	FP-R	SR-R	FP-R	SR-R	FP-R	SR-R
Stiff corn porridge	36	41 (25.6)	30 (15.3)	43 (25.2)	0.4	0.7	0.4^a^	0.6^a^	-10	+2	16	15	39	37	-18	10	61	67
Rice, boiled	44	21 (19.6)	23 (22.7)	32 (34.9)	0.8	0.6	0.8^a^	0.8^a^	+1	+10	10	13	45	69	-4	36	52	59
Spaghetti	22	54 (30.6)	37 (20.4)	60 (40.3)	0.3	0.8	0.3	0.9^a^	-16	+7	24	14	44	26	-28	10	46	86
Cowpea with rice	23	35 (23.8)	28 (18.8)	46 (36.6)	0.7	0.5	0.6^a^	0.6^a^	-7	+12	12	18	35	51	-19	18	52	61
Fermented millet porridge	26	58 (39.7)	47 (30.0)	64 (41.3)	0.8	0.9	0.8^a^	0.8^a^	-11	+6	17	14	29	24	-12	10	65	89
Dish, leafy vegetables	23	52 (28.2)	46 (22.1)	54 (31.8)	0.7	0.6	0.6^a^	0.6^a^	-6	+3	13	15	25	29	-8	2	78	78
Sauce, groundnut paste	19	15 (13.4)	13 (13.7)	18 (11.3)	0.8	0.8	0.6^a^	0.9^a^	-2	+4	6	6	40	40	-25	28	63	63
Sauce, vegetables	26	31 (19.6)	25 (17.1)	36 (27.4)	0.6	0.7	0.7^a^	0.8^a^	-6	+6	11	13	35	42	-24	10	58	69
Sauce, dry okra	24	32 (17.0)	26 (12.7)	32 (14.9)	0.7	0.6	0.6^a^	0.4^a^	-7	-1	9	10	27	32	-20	2	71	71

^a^p <0.05

^b^WR (g) = Mean difference between served portions and leftovers

^c^Lin’s agreement coefficient measures the agreement between the estimated weight and the actual weight. It ranges from -1 to +1 where the values of -1, 0 and +1 indicate perfect discordance, zero concordance, and perfect concordance, respectively. According to Landis and Koch [28], the thresholds for interpretation of the coefficient values are: < 0 "Poor", 0–0.2 "Slight", 0.21–0.4 "Fair", 0.41–0.60 "Moderate", 0.61–0.80 "Substantial", 0.81–1.00 "Almost perfect".

^d^Mean difference (g) = Mean difference between FP-R and WR or Mean difference between SR-R and WR

^e^Mean absolute difference (g) = Mean (|FP-R—WR|) / / Mean (|SR-R–WR|).

^f^Mean error = (Mean absolute difference (g) / mean WR) *100

^g^Bland Altman’s graphical analysis by relative difference: X (Mean Weight) = (FP-R + WR)/2 // (SR-R + WR)/2; Y (relative difference) = (FP-R—WR)/ Mean Weight // (SR-R—WR)/ Mean Weight.

For most food items, the Lin’s coefficients of the portions estimated by FP-R and WR varied from 0.6 to 0.8, indicating substantial concordance (Table 2), except for spaghetti and stiff corn porridge (Lin’s coefficients = 0.3 and 0.4, respectively, indicating fair concordance). For the SR-R estimates, the Lin’s coefficients (from 0.5 to 0.9) showed moderate, substantial, and almost perfect concordance with the WR in function of the food item (Table 2).

**Fig 4:**
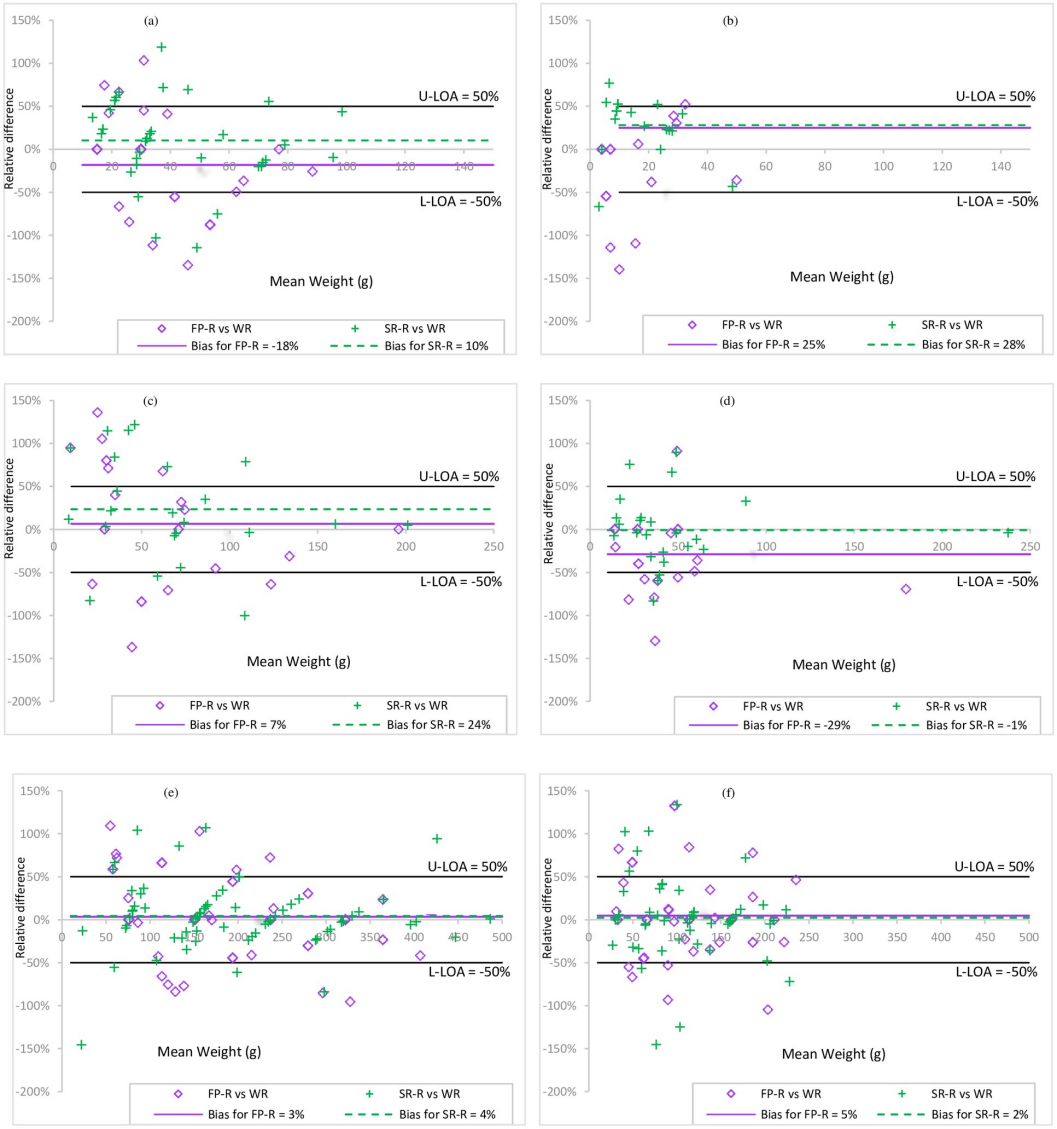
Comparison of the portion sizes estimated with the two methods (FP-R and SR-R) with the consumed portion size (WR) using Bland Altman plots. (a) Stiff cereal porridge eaten by 6-11-month-old children; (b) Groundnut paste sauce eaten by 6-11-month-old children; (c) Stiff cereal porridge eaten by 12-23-month-old children; (d) Groundnut paste sauce eaten by 12-23-month-old children; (e) Stiff cereal porridge eaten by women; (f) Groundnut paste sauce eaten by women.

The Bland and Altman plots of FP-R-based estimates and WR showed biases that ranged from -4 to -28%, with the largest bias for spaghetti (-28%) (Table 2); depending on the food item, 46 to 78% of the estimates were between the upper and lower limits of ± 50%. The biases of SR-R versus WR were smaller for most food items (from 2 to 18%) but were higher for the groundnut paste sauce (28%) and rice (36%). The percentage of estimates between the upper and lower limits of ± 50% ranged from 59% to 89% (Fig 4a and 4b; Table 2).

### For 12-23-month-old children

The Spearman’s correlation coefficients between FP-R and WR were low for several food items (meat, r = 0.4, p <0.05) and sometimes not significant (leafy vegetable dish, r = 0.3, p >0.05; fish, 0.4, p >0.05). For other food items they were positive and significant (r = 0.5 to 0.8, p <0.05) (Table 3), but mostly lower than those obtained by SR-R. The mean crude differences between the quantities estimated by FP-R and those actually consumed (WR) ranged from -28 to +0.1 g, and were mostly due to underestimation, as observed for the 6–11 months group. Between the quantities estimated by SR-R and the WR, it ranged from -15 to +9 g (Table 3). Similarly, the means of absolute differences varied more widely (from 5 to 47 g and from 6 to 28 g for the estimates by FP-R and by SR-R, respectively). In agreement, the mean errors varied from 37 to 65% (FP-R) and from 21 to 58% (SR-R). The FP-R method showed a tendency to underestimate all food items except fish, while the SR-R-based estimates were almost equally distributed below and above the reference values.

**Table 3:** Comparison between the actual quantity consumed (weighed record -WR) and the consumed portion sizes estimated by 24-hour recall using the food photography atlas (FP-R) or salted replicas (SR-R) for 12-23-month-old children.

	12-23-month-old children																	
	n	WR^b^ (g) Mean (SD)	FP-R (g) Mean (SD)	SR-R (g) Mean (SD)	Lin’s concordance coefficient^c^		Spearman’s correlation *r*		Mean difference^d^ (g)		Mean |difference|^e^ (g)		|Mean error|^f^ (%)		Bland Altman Analysis^g^			
															Bias (%)		Measurements between -50% and +50% limits (%)	
					FP-R	SR-R	FP-R	SR-R	FP-R	SR-R	FP-R	SR-R	FP-R	SR-R	FP-R	SR-R	FP-R	SR-R
Stiff corn porridge	23	63 (52.1)	57 (40.4)	71 (49.0)	0.7	0.7	0.5^a^	0.6^a^	-6	+9	26	26	42	42	7	24	44	57
Rice, boiled	46	54 (39.4)	44 (27.8)	56 (38.2)	0.4	0.5	0.6^a^	0.8^a^	-9	+3	24	23	44	43	-7	13	54	67
Spaghetti	22	71 (39.1)	55 (32.8)	62 (37.6)	0.5	0.8	0.6^a^	0.7^a^	-15	-9	28	17	39	24	-22	-15	59	73
Cowpea with rice	18	62 (45.5)	50 (27.4)	60 (28.9)	0.3	0.5	0.6^a^	0.5^a^	-12	-2	28	28	55	46	-9	8	56	61
Fermented millet porridge	25	105 (78.4)	99 (80.0)	95 (67.1)	0.7	0.8	0.8^a^	0.8^a^	-6	-10	47	24	44	23	-3	-3	48	80
Dish, leafy vegetables	22	84 (66.5)	56 (34.4)	69 (42.8)	0.1	0.6	0.3	0.8^a^	-28	-15	46	26	55	31	-24	-13	41	86
Sauce, groundnut paste	24	47 (45.4)	33 (23.8)	46 (44.7)	0.6	0.9	0.6^a^	0.6^a^	-15	-1	18	13	39	27	-29	-1	63	75
Sauce, vegetables	26	52 (32.0)	34 (19.1)	48 (24.2)	0.4	0.7	0.5^a^	0.7^a^	-17	-3	22	15	42	28	-32	2	62	81
Sauce, dry okra	18	46(25.4)	33 (22.1)	48 (20.1)	0.5	0.9	0.5^a^	0.9^a^	-13	+2	17	10	37	21	-27	17	56	78
Pieces of beef, stewed	32	9 (5.7)	5 (2.3)	13(7.5)	0.2	0.4	0.4^a^	0.5^a^	-4	+3	5	6	52	58	-43	28	41	56
Fish	18	12 (7.2)	12 (8.1)	13 (9.0)	0.2	0.4	0.4	0.7^a^	+0.1	+2	8	6	65	47	3.2	15	44	61

^a^p <0.05

^b^WR (g) = Mean difference between served portions and leftovers

^c^Lin’s agreement coefficient measures the agreement between the estimated weight and the actual weight. It ranges from -1 to +1 where the values of -1, 0 and +1 indicate perfect discordance, zero concordance, and perfect concordance, respectively. According to Landis and Koch [28], the thresholds for interpretation of the coefficient values are: < 0 "Poor", 0–0.2 "Slight", 0.21–0.4 "Fair", 0.41–0.60 "Moderate", 0.61–0.80 "Substantial", 0.81–1.00 "Almost perfect".

^d^Mean difference (g) = Mean difference between FP-R and WR or Mean difference between SR-R and WR

^e^Mean absolute difference (g) = Mean (|FP-R—WR|) / / Mean (|SR-R–WR|).

^f^Mean error = (Mean absolute difference (g) / mean WR) *100

^g^Bland Altman’s graphical analysis by relative difference: X (Mean Weight) = (FP-R + WR)/2 // (SR-R + WR)/2; Y (relative difference) = (FP-R—WR)/ Mean Weight // (SR-R—WR)/ Mean Weight.

The concordance between FP-R estimates and WR was poor for the leafy vegetable dish, meat, and fish (Lin’s coefficients ≤ 0.2), fair for cowpea with rice, rice and vegetable sauce (Lin’s coefficients = 0.3 to 0.4), and moderate and substantial for the other food items (Lin’s coefficients = 0.5 to 0.7). The concordance between SR-R estimates and WR ranged from moderate, to almost perfect (Lin’s coefficients = 0.5 to 0.9), as observed in the 6–11 months group, except for meat and fish (Lin’s coefficients = 0.4, fair) (Table 3).

The mean biases (Bland and Altman analysis) were higher between FP-R and WR than between SR-R and WR, ranging from -43% (for meat) to +7% and from -13% to +28%, respectively; the percentage of observations between the limits of ± 50% varied from 41% to 63% and from 56% to 86%, respectively (Fig 4b and 4c and Table 3).

### For women of childbearing age

Overall, mothers estimated their own consumed portions more accurately than those of their children. FP-R and SR-R-based estimates were positively and significantly correlated with WR (r = 0.5 to 0.8, p <0.05; r = 0.6 to 0.9, p<0.05) (Table 4). The mean crude differences between FP-R and WR and between SR-R and WR ranged from -30 to +44 g and from -32 to +9 g, respectively. The mean absolute differences varied from 14 to 100g for FP-R and from 7 to 68g for SR-R. No clear trend (under/overestimation) appeared for women concerning the FP-R-based estimates, unlike in children (underestimation) (Table 4). Conversely, SR-R tended to underestimate the portions by 17–35% (for seven of the eleven food items). The concordance between estimates (FP-R and SR-R) and reference were moderate, substantial and almost perfect for all food items (Lin’s coefficients = 0.6 to 0.9), except for meat portions estimated by FP-R (Lin’s coefficient = 0.4, fair) (Table 4). The Bland and Altman plots between FP-R and SR-R and WR showed mean biases from -26% to +31% (with higher biases for meat and fish) and from -12% to +15%, respectively (Table 4). Moreover, 63% to 79% and 75% to 91% of FP-R- and SR-R-based estimates, respectively, were within the ± 50% limits, except for meat by FP-R (only 46% of estimates) and fish by SR-R (58% of estimates) (Fig 4e and 4f).

**Table 4:** Comparison between the actual quantity consumed (weighed record -WR) and the consumed portion sizes estimated by 24-hour recall using the food photography atlas (FP-R) or salted replicas (SR-R) for women of childbearing age.

	Women of childbearing age																	
	n	WR^b^ (g) Mean (SD)	FP-R (g) Mean (SD)	SR-R (g) Mean (SD)	Lin’s concordance coefficient^c^		Spearman’s correlation *r*		Mean difference^d^ (g)		Mean |difference|^e^ (g)		|Mean error|^f^ (%)		Bland Altman Analysis^g^			
															Bias (%)		Measurements between -50% and +50% limits (%)	
					FP-R	SR-R	FP-R	SR-R	FP-R	SR-R	FP-R	SR-R	FP-R	SR-R	FP-R	SR-R	FP-R	SR-R
Stiff corn porridge	68	195 (113)	189 (92.4)	204 (117)	0.7	0.8	0.7^a^	0.8^a^	-6	+9	51	44	26	23	3	4	74	84
Rice, boiled	113	235 (110)	253 (115)	209 (104)	0.6	0.7	0.6^a^	0.7^a^	+19	-25	71	59	30	25	7	-12	70	77
Spaghetti	48	181 (158)	162 (148)	171 (124)	0.9	0.9	0.8^a^	0.9^a^	-20	-10	52	43	29	24	-13	15	67	81
Cowpea with rice	51	175 (118)	155 (89.6)	150 (87.8)	0.6	0.7	0.7^a^	0.7^a^	-19	-25	61	61	35	35	-2	-8	71	79
Fermented millet porridge	58	377 (171)	420 (173)	345 (166)	0.7	0.8	0.7^a^	0.8^a^	+44	-32	100	64	27	17	13	-10	71	91
Dish, leafy vegetables	48	291 (202)	261 (159)	260 (161)	0.8	0.8	0.7^a^	0.7^a^	-30	-32	70	68	24	23	-8	-11	79	79
Sauce, groundnut paste	49	111 (65.4)	113 (61.7)	110 (58.3)	0.6	0.7	0.6^a^	0.7^a^	+2	-1	34	31	30	28	5	-2	74	80
Sauce, vegetables	63	125 (66.3)	133 (64.0)	128 (55.9)	0.8	0.7	0.6^a^	0.7^a^	+8	+3	32	33	26	27	9	6	76	76
Sauce, dry okra	52	71 (40.6)	74 (44.0)	73 (43.7)	0.6	0.6	0.6^a^	0.6^a^	+2	+1	22	24	30	33	6	3	71	75
Pieces of beef, stewed	65	26 (21.7)	18 (13.8)	26 (18.4)	0.4	0.8	0.5^a^	0.8^a^	-8	0	14	8	53	31	-26	8	46	75
Fish	24	48 (30.8)	70 (48.8)	43 (30.0)	0.7	0.7	0.7^a^	0.6 ^a^	+22	-5	27	7	56	35	31	-12	63	58

^a^p <0.05

^b^WR (g) = Mean difference between served portions and leftovers

^c^Lin’s agreement coefficient measures the agreement between the estimated weight and the actual weight. It ranges from -1 to +1 where the values of -1, 0 and +1 indicate perfect discordance, zero concordance, and perfect concordance. According to Landis and Koch [28], the thresholds for interpretation of the coefficient values are: < 0 "Poor", 0–0.2 "Slight", 0.21–0.4 "Fair", 0.41–0.60 "Moderate", 0.61–0.80 "Substantial", 0.81–1.00 "Almost perfect".

^d^Mean difference (g) = Mean difference between FP-R and WR or Mean difference between SR-R and WR

^e^Mean absolute difference (g) = Mean (|FP-R—WR|) / / Mean (|SR-R–WR|).

^f^Mean error = (Mean absolute difference (g) / mean WR) *100

^g^Bland Altman’s graphical analysis by relative difference: X (Mean Weight) = (FP-R + WR)/2 // (SR-R + WR)/2; Y (relative difference) = (FP-R—WR)/ Mean Weight // (SR-R—WR)/ Mean Weight.

### Percentage of the recommended nutrient daily intake corresponding to the mean differences

To better understand the results and to assess the importance of the portion differences obtained with the two estimation methods relative to WR, we converted the mean absolute differences into percentages of the recommended intakes of energy and of five nutrients (protein, iron, zinc and vitamin A and vitamin B9) for each age group [31, 32]. The obtained percentages of recommended energy intakes were low (<5%) for all foods, but for spaghetti (7% for the FP-R method) (Tables 5–7). For 6-11-month-old children and for women, the mean differences with both methods represented <5% of the recommended intakes of the five nutrients for eight food items. However, they were >5% and could reach 17% for cowpea with rice (for protein, zinc and vitamin B9), spaghetti (for energy and protein), and leafy vegetables dish (for vitamin A). For the 12–23 months group, where we observed higher differences with both estimation methods, the percentages of required nutrients were often >5%, mainly for protein, but also iron and vitamins A and B9.

**Table 5:** Percentages of the recommended energy and nutrient intakes corresponding to the mean absolute differences between estimation and reference methods for 6-11-month-old children.

	FP-R vs. WR						
	Mean |difference|^a^ (g)	% of RNI^b^					
		Energy	Protein	Iron^c^	Zinc^c^	Vit A	Folate
Stiff corn porridge	16	1.3	2.4	0.5	0.8	0.0	2.1
Rice, boiled	10	1.6	2.0	0.2	0.5	0.0	0.4
Spaghetti, cooked in sauce	24	**6.7**	**8.9**	0.8	1.3	2.1	2.7
Cowpea with rice	12	2.0	**6.4**	1.4	1.4	0.0	**9.0**
Fermented millet porridge	17	1.0	1.5	1.6	0.6	0.0	3.1
Dish, leafy vegetables	13	1.2	3.6	1.6	0.7	4.1	3.7
Sauce, groundnut paste	6	0.9	2.2	0.3	0.4	0.3	0.8
Sauce, vegetables with meat	11	1.4	2.3	0.8	0.6	0.4	0.4
Sauce, dry okra	9	1.7	3.6	0.7	0.8	0.4	2.9
	SR-R vs. WR						
	Mean |difference|^a^ (g)	% of RNI^b^					
		Energy	Protein	Iron^c^	Zinc^c^	Vit A	Folate
Stiff corn porridge	15	1.3	2.3	0.5	0.7	0.0	1.9
Rice, boiled	13	2.0	2.6	0.3	0.7	0.0	0.5
Spaghetti, cooked in sauce	14	3.9	**5.2**	0.5	0.7	1.2	1.6
Cowpea with rice	18	3.0	**9.6**	2.0	2.2	0.0	**13.5**
Fermented millet porridge	14	0.8	1.3	1.3	0.5	0.0	2.5
Dish, leafy vegetables	15	1.4	4.1	1.9	0.8	4.8	4.3
Sauce, groundnut paste	6	0.9	2.2	0.3	0.4	0.3	0.8
Sauce, vegetables with meat	13	1.6	2.8	1.0	0.7	0.4	0.4
Sauce, dry okra	10	1.9	4.1	0.8	0.9	0.4	3.3

**in bold**: percentages >5.0% of the RNI

^a^Mean absolute difference (g) = Mean (|FP-R—WR|) / / Mean (|SR-R–WR|).

^b^Recommended Nutrient Intake [31, 32]

^c^Low bioavailability: 5% for iron and 15% for zinc.

**Table 6:** Percentages of the recommended energy and nutrient intakes corresponding to the mean absolute differences between estimation and reference methods for 12-23-month-old children.

	FP-R vs. WR						
	Mean |difference|^a^ (g)	% of RNI^b^					
		Energy	Protein	Iron^c^	Zinc^c^	Vit A	Folate
Stiff corn porridge	26	1.5	3.9	1.3	1.3	0.0	1.8
Rice, boiled	24	2.6	4.6	0.8	1.2	0.0	0.5
Spaghetti, cooked in sauce	28	**5.4**	**10.1**	1.4	1.5	2.5	1.7
Cowpea with rice	28	3.2	**14.6**	**5.1**	3.4	0.1	**11.2**
Fermented millet porridge	47	1.9	4.2	**7.0**	1.6	0.1	4.5
Dish, leafy vegetables	46	2.9	**12.4**	**9.1**	2.6	**14.6**	**7.1**
Sauce, groundnut paste	18	1.9	**6.5**	1.5	1.2	0.8	1.3
Sauce, vegetables with meat	22	1.9	4.5	2.7	1.1	0.8	0.4
Sauce, dry okra	17	2.3	**6.7**	2.2	1.6	0.7	2.9
Pieces of beef, stew	5	2.1	**10.3**	0.6	2.6	0.4	0.4
Fish, boiled	8	1.3	**17.9**	1.0	0.6	0.1	0.1
	SR-R vs. WR						
	Mean |difference|^a^ (g)	% of RNI^b^					
		Energy	Protein	Iron^c^	Zinc^c^	Vit A	Folate
Stiff corn porridge	26	1.5	3.9	1.3	1.3	0.0	1.8
Rice, boiled	24	0.9	2.1	3.6	0.8	0.0	2.3
Spaghetti, cooked in sauce	28	3.2	**14.6**	**5.1**	3.4	0.1	**11.2**
Cowpea with rice	23	2.5	4.4	0.8	1.2	0.0	0.5
Fermented millet porridge	17	3.3	**6.1**	0.9	0.9	1.5	1.0
Dish, leafy vegetables	26	1.6	**7.0**	**5.2**	1.5	8.3	4.0
Sauce, groundnut paste	13	1.4	4.7	1.1	0.8	0.6	0.9
Sauce, vegetables with meat	15	1.3	3.1	1.8	0.8	0.5	0.3
Sauce, dry okra	10	1.3	3.9	1.3	0.9	0.4	1.7
Pieces of beef, stew	6	2.5	**12.4**	0.8	3.1	0.5	0.5
Fish	6	1.0	**13.4**	0.8	0.4	0.1	0.1

**in bold**: percentages >5.0% of the RNI

^a^Mean absolute differences (g) = Mean (|FP-R—WR|) / / Mean (|SR-R–WR|).

^b^Recommended Nutrient Intake [31, 32].

^c^Low bioavailability: 5% for iron and 15% for zinc.

**Table 7:** Percentages of the recommended energy and nutrient intakes corresponding to the mean absolute differences between estimation and reference methods for women of childbearing age.

	FP-R vs. WR						
	Mean |difference|^a^ (g)	% of RNI^b^					
		Energy	Protein	Iron^c^	Zinc^c^	Vit A	Folate
Stiff corn porridge	44	1.5	1.7	0.4	1.8	0.0	1.1
Rice, boiled	59	3.7	3.0	0.4	2.5	0.0	0.4
Spaghetti, cooked in sauce	43	4.8	4.0	0.4	1.9	3.0	1.0
Cowpea with rice	61	4.0	**8.2**	2.2	**6.3**	0.1	**9.2**
Fermented millet porridge	64	1.4	1.5	1.9	1.9	0.1	2.3
Dish, leafy vegetables	31	1.9	2.9	0.5	1.7	1.1	0.8
Sauce, groundnut paste	33	1.6	1.8	0.8	1.5	0.9	0.2
Sauce, vegetables with meat	24	1.8	2.5	0.6	1.9	0.8	1.6
Sauce, dry okra	68	2.5	4.7	2.7	3.3	**17.3**	3.9
Pieces of beef, stew	8	1.9	4.3	0.2	3.6	0.5	0.2
Fish	7	0.6	4.0	0.2	0.4	0.1	0.0
	SR-R vs. WR						
	Mean |difference|^a^ (g)	% of RNI^b^					
		Energy	Protein	Iron^c^	Zinc^c^	Vit A	Folate
Stiff corn porridge	51	1.7	2.0	0.5	2.1	0.0	1.3
Rice, boiled	71	4.4	3.6	0.5	3.0	0.0	0.5
Spaghetti, cooked in sauce	52	**5.7**	4.9	0.5	2.3	3.6	1.2
Cowpea with rice	61	4.0	**8.2**	2.2	**6.3**	0.1	**9.2**
Fermented millet porridge	100	2.3	2.3	2.9	3.0	0.1	3.6
Dish, leafy vegetables	34	2.0	3.2	0.5	1.9	1.2	0.9
Sauce, groundnut paste	32	1.6	1.7	0.8	1.4	0.9	0.2
Sauce, vegetables with meat	22	1.7	2.2	0.6	1.8	0.7	1.4
Sauce, dry okra	70	2.5	4.9	2.7	3.4	**17.8**	4.0
Pieces of beef, stew	14	3.3	**7.5**	0.4	**6.2**	0.9	0.4
Fish	27	2.5	**15.6**	0.7	1.6	0.3	0.1

**in bold**: percentages >5.0% of the RNI

^a^Mean absolute difference (g) = Mean (|FP-R—WR|) / / Mean (|SR-R–WR|).

^b^Recommended Nutrient Intake [31, 32].

^c^Low bioavailability: 5% for iron and 15% for zinc.

## Discussion

In this recall survey, we assessed the accuracy and precision of estimation methods based on the use of a FP atlas and SRs compared with WR, considered as the reference. We used different, complementary statistical methods to analyze the results and to interpret our findings. For most of the eleven food items tested, the portions estimated with the FP atlas and SRs were positively correlated and showed relatively high concordances with the reference method. However, the correlations with WR for the portions of stiff corn porridge and spaghetti consumed by 6-11-month-old children and of leafy vegetable dish, meat and fish consumed by 12-23-month-old children, estimated using the FP atlas by their mothers, were insufficient (Refer to Tables 2–4). Moreover, the Lin’s coefficients revealed weak concordances for cowpea with rice, rice, and vegetable sauce in the 12–23 months group. The mean crude differences (<30g for children and <45g for women) were low, compared with findings from similar studies [16, 25, 34]. However, when we used the absolute values to eliminate the compensation biases associated with the sign of the difference between estimated and reference values, the mean errors were higher and reached 65% and 69% of the WR with the FP-R and the SR-R methods, respectively. Nevertheless, the crude differences give information about possible under- or over- estimations. The Bland and Altman plots clearly showed that the SR-R allowed estimating the consumed portions with fewer errors than the FP-R (56 to 91% vs 46 to 79% of estimations between the limits of ± 50%, respectively) (Refer to Fig 4).

Perception and conceptualization of food and memory of the food quantity consumed strongly influence the accuracy and precision of food portion size estimation when using a FP atlas [23, 24, 35]. Perception and conceptualization biases depend on the responder’s capacity to mentally construct the food quantities consumed or seen in their plates and to transform them into portion sizes represented in photos. The memory bias corresponds to the responder’s capacity to remember a portion of food consumed or seen in their plates. This bias is not intrinsically linked to the use of FP atlases, and must be considered also in the SR-R. To improve the result quality, we followed some suggestions proposed by other authors [16, 36]. Specifically, we informed participants about the study purpose before the measurements and served them the food portions to be estimated in the same plates and bowls used for the photographs. We also asked mothers to estimate the portions that they had visualized by estimating first the served portion and then the leftovers, if applicable, in order to calculate the portion consumed. Therefore, leftovers, when there were any, were estimated using the same estimation methods. In some studies, other methods were used to estimate leftovers (smaller portion size photos, household measurements) [17, 19, 37]. Vossenaar et al. suggested to directly estimate the quantities consumed [10]. Other parameters can affect the estimate accuracy, such as the food nature and texture (sauces, porridge, solid foods), the photograph format (framing, size, color and photo arrangement in catalogs), or the portion representation (portions in photographs or virtual). Nelson and Haraldsdottir validated an atlas based on photographs of 7.5x10cm in size [35]. Another study obtained more accurate estimates when eight (numerical) portions were presented simultaneously compared with four [38]. Our FP atlas had color photographs of 7x9cm in size and allowed participants to concomitantly visualize all nine portions (four photograph portions and five virtual portions). Moreover, photographs for only four portions can limit the bias related to the choice of the middle portion when an odd number of portion photographs is presented. In young children who are actively growing, the quantities consumed are strongly linked to the age. For this reason, we used two portion size ranges adapted to the two age groups to improve the measurement accuracy, as done in previous studies [17, 39].

Although differences in the design among studies do not facilitate comparisons, our results can be compared with some studies on FP atlas validation in adults. Depending on the food items, the mean differences ranged from an underestimation of 40g to an overestimation of 19g [16, 25, 34]. Some studies recorded lower errors (from -35 to +40%) with an FP atlas [16, 25, 40–43], but their data were not expressed in absolute values. In our study, errors in the estimated portion sizes increased with the average portion size consumed and were smaller for 6-11-month-old than for 12-23-month-old children. Few studies reported the validation of a FP atlas developed for <2-year-old children. Three studies in children and adolescents with recalls immediately after consumption found mean differences between -40 and 49g (with errors between -24 to +20%), depending on the food item [19, 21, 44]. Using an age-appropriate FP atlas, Foster et al. concluded that parents underestimate their pre-school children’s served portions by 5%, on average, and overestimated their consumed portions by 7% (with agreement limits from -75 to +357%) [37].

In our study, the accuracy and precision of the estimates varied strongly in function of the food item and age group. The eleven food items selected for assessment had various shapes and textures. When using the FP atlas to estimate portions in children, stiff corn porridge and spaghetti, in the 6–11 months group, and cowpea with rice, leafy vegetable dish, meat, and fish, in the 12–23 months group, displayed the least satisfactory results. Most of these food items are amorphous (without fixed form). Some studies reported that portions are more correctly estimated for solid than amorphous food items [12, 14]. Huybregts et al. [16] also found the highest differences between photograph-based estimates and weighed records for amorphous food items, such as rice and millet couscous. Visual cues (e.g. the food geometric shape and the plate surface coverage) may also influence the accuracy of portion size estimates when using photographs [40]. The estimation of the stiff corn porridge portion size may have been complicated by the fact that it was served in various forms (ball or other), as traditionally done, that sometimes differed from those shown in the FP atlas. Similarly, for fish, different parts (head, tail, body) could be served, thus complicating the estimation. Moreover, the bones included in the meat or fish portions influenced the participants’ capacity to estimate the actual consumed quantities. On the other hand, we cannot clearly explain the high differences observed for spaghetti, an amorphous food served during the study.

Mothers estimated their own consumed portions with smaller errors than for their children. Indeed, the many leftovers and waste, observed during the children’s meal consumption, may have increased the difficulty of portion estimation during recalls. Moreover, the great mobility of 12-23-month-old children at mealtimes further increased waste and the estimation error risk. The importance of the relative differences was also accentuated by the smaller portion sizes consumed by young children. For children, mothers tended to underestimate the consumed portions with the FP atlas, and to overestimate them with the SR. Conversely, women tended to underestimate their own consumed portion with the SR, without any clear tendency with the FP atlas. This could be due to a desirability bias, which is inherent to recall methods. For example, underestimation is frequent in 24-hour recall surveys where participants under-report the number of foods consumed, the meal frequencies, and the consumed quantities [45].

Lastly, quantification of the nutritional impact of the mean differences of the estimates showed that for most foods, they would represent <5% of the requirements in energy, protein, and iron, zinc, vitamin A and vitamin B9 (i.e. the micronutrients most implicated in deficiencies) (Refer to Tables 5–7). In their food atlas validation study, Badari et al. found that the energy, protein and micronutrient intakes were estimated with relative errors of +8.1, +9.2%, and -2 to +3%, respectively [40]. Robson and Livingstone validated their food atlas with acceptable mean differences of ±10% for most nutrients [46]. Harris-Fry et al. found that their food atlas underestimated energy, protein, and iron intakes by 130 kcal, 4g, and 0.5 mg, respectively [42]. Thus, the FP atlas developed in the present study could be used to conduct food consumption surveys in Burkina Faso, but it must be kept in mind that errors could be accumulated in a complete 24-hour recall survey if some food items were eaten several times in the same day.

### Limitations of the study

For some of the tested food items, although we had enrolled seven additional mother-child pairs, the number of measurements was lower than expected, mainly due to refusal to eat them, and this may have affected the power of the statistical analyses. Furthermore, the choice of estimating served portions and also leftovers led to cumulate estimation errors.

## Conclusion

Several previous studies validated food portion size atlases for food consumption surveys based mainly on crude mean differences. Completing this indicator with other statistical analyses allowed us to highlight some limitations, by assessing more holistically the accuracy of methods based on FP atlases and SRs. Overall, our results showed a better estimation with the SR-based method. Whatever the estimation method, portions consumed by young children were estimated with less accuracy and precision than those consumed by women. This highlights the difficulty mothers have in perceiving the quantities of food actually consumed by their young children. The food atlas developed in this study could be partly used as a portion size assessment tool in food consumption surveys targeting young children and women in Ouagadougou. Indeed, only the food items for which portion estimation by FP-R was closest to the reference method could be presented, thus limiting the use of SRs to the least well estimated foods (spaghetti, meat, and fish). The estimation error could be reduced by developing a specific tool for leftover estimation. As more and more food consumption data are collected electronically, the use of digital FP atlases in this context should also be considered.

## Supporting information

S1 Checklist Inclusivity in global research. (DOCX)

S1 File Food photography atlas with portion weights. (PDF)
