# Supplementary material for: Estimation of food portion sizes in women of childbearing age and young children in Ouagadougou (Burkina Faso) using a food photography atlas and salted replicas: Comparison with weighed records
Source: PLoS One. 2023 Sep 18;18(9):e0291375. doi: 10.1371/journal.pone.0291375 (PMC10506719; doi:10.1371/journal.pone.0291375)
Supplement: S1 File — (PDF) [file pone.0291375.s002.pdf]

S2. Supporting Information: Food photography atlas and portion weights.

This atlas contains 279 portion sizes of 11 dishes or dishes components of commonly consumed in Ouagadougou, from which 124 in photos. It's subdivided into 3 parts: for 6-11-months-children, 12-23-months-children and for 15-49-years-women. It was produced as part of the evaluation of the Meriem project and is intended to dietary surveys.

Food photography atlas

September 2020

This food atlas is published in MERIEM project (www.meriem-nutrition.org) coordinated by GRET and HYSTRA

PART 1:

PORTION SIZES FOR 6-11-MONTH-OLD CHILDREN

1. Fermented millet porridge ---------------------------------- 3
2. Stiff corn porridge --- 3
3. Rice, boiled --- 5
4. Cowpea with rice --- 5
5. Spaghetti -- 7
6. Sauce, groundnut paste -- 7
7. Sauce, vegetables (djabadji)----------------------------------- 9
8. Sauce, dry okra --- 9
9. Dish, leafy vegetables (babenda) ------------------------ 11

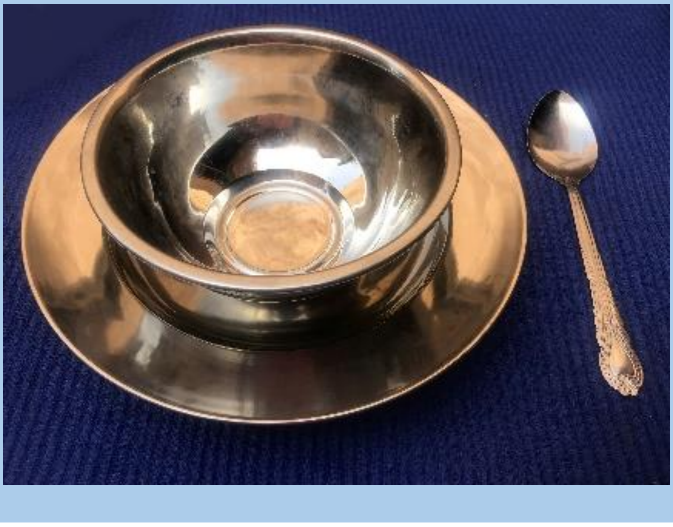

❶ 6-11-month – Fermented millet porridge

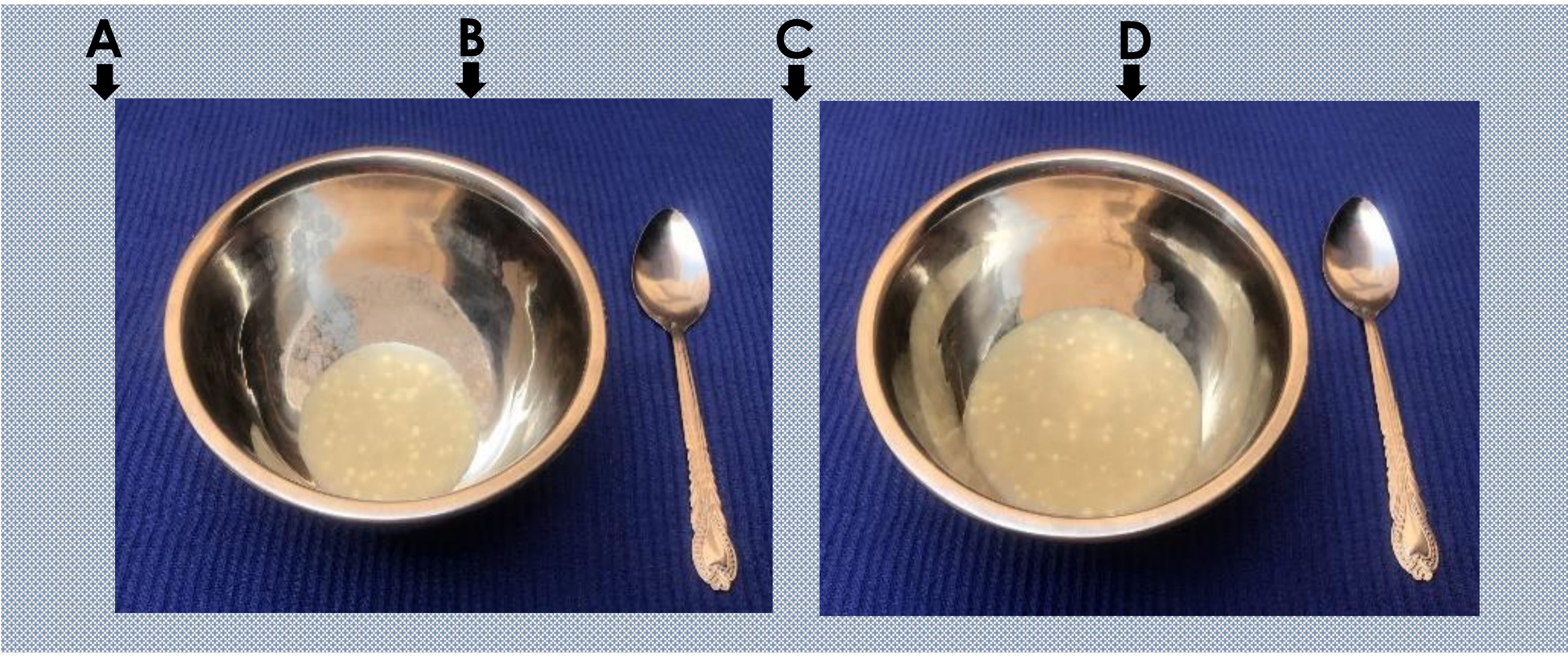

❷ 6-11-month – Stiff corn porridge

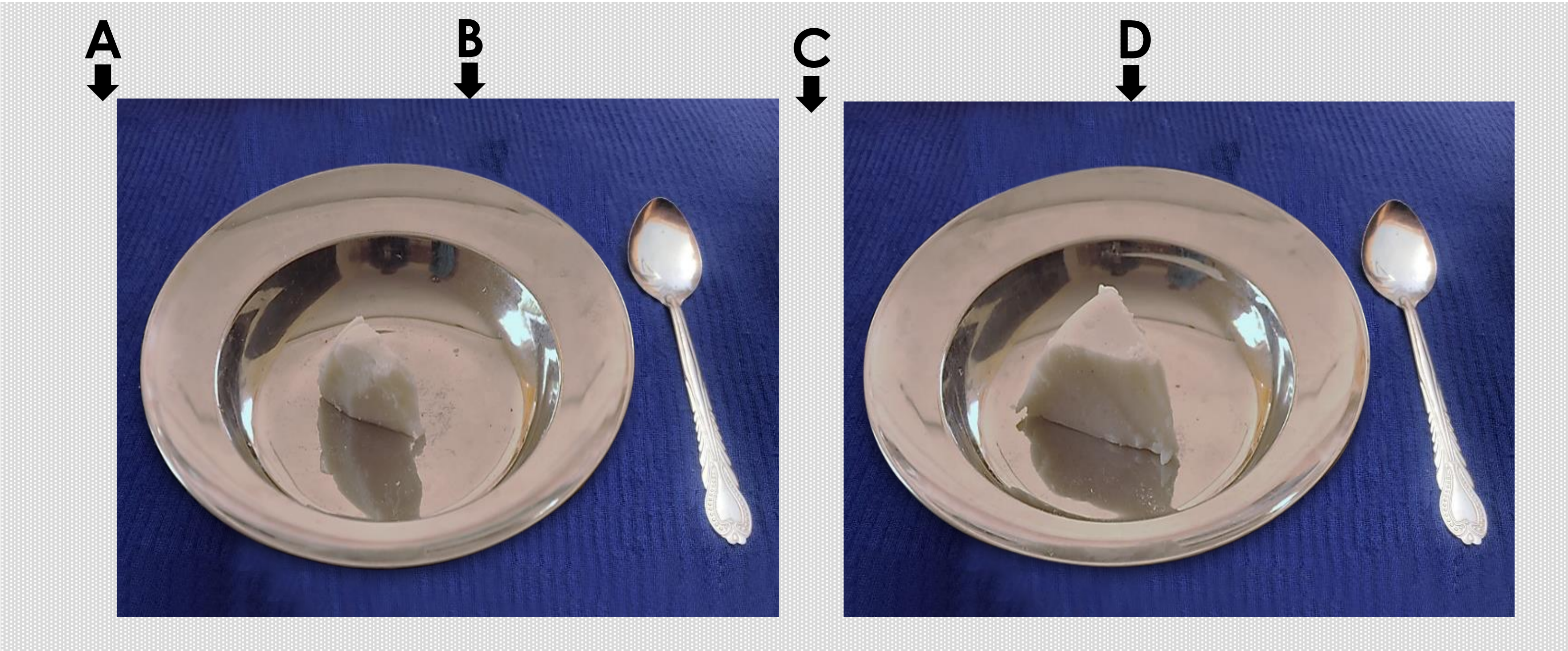

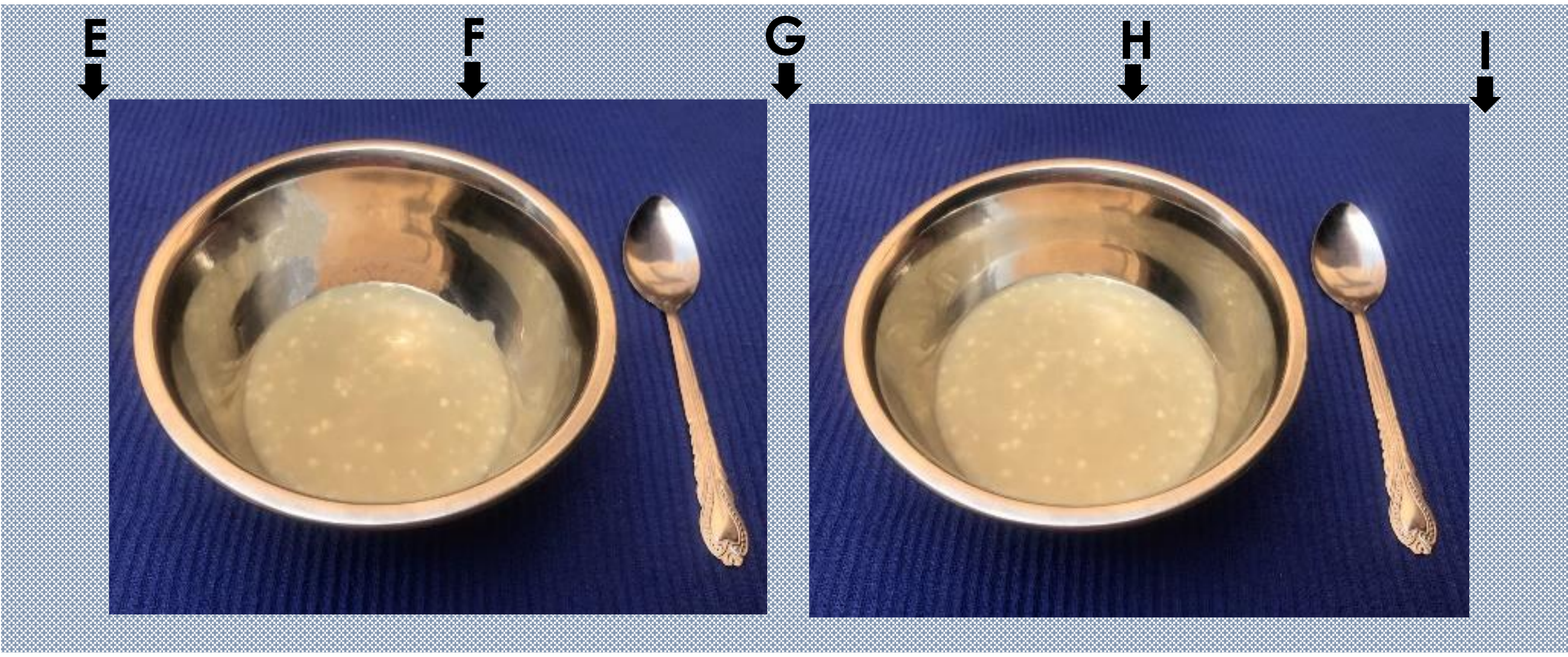
E
F
G
H
I

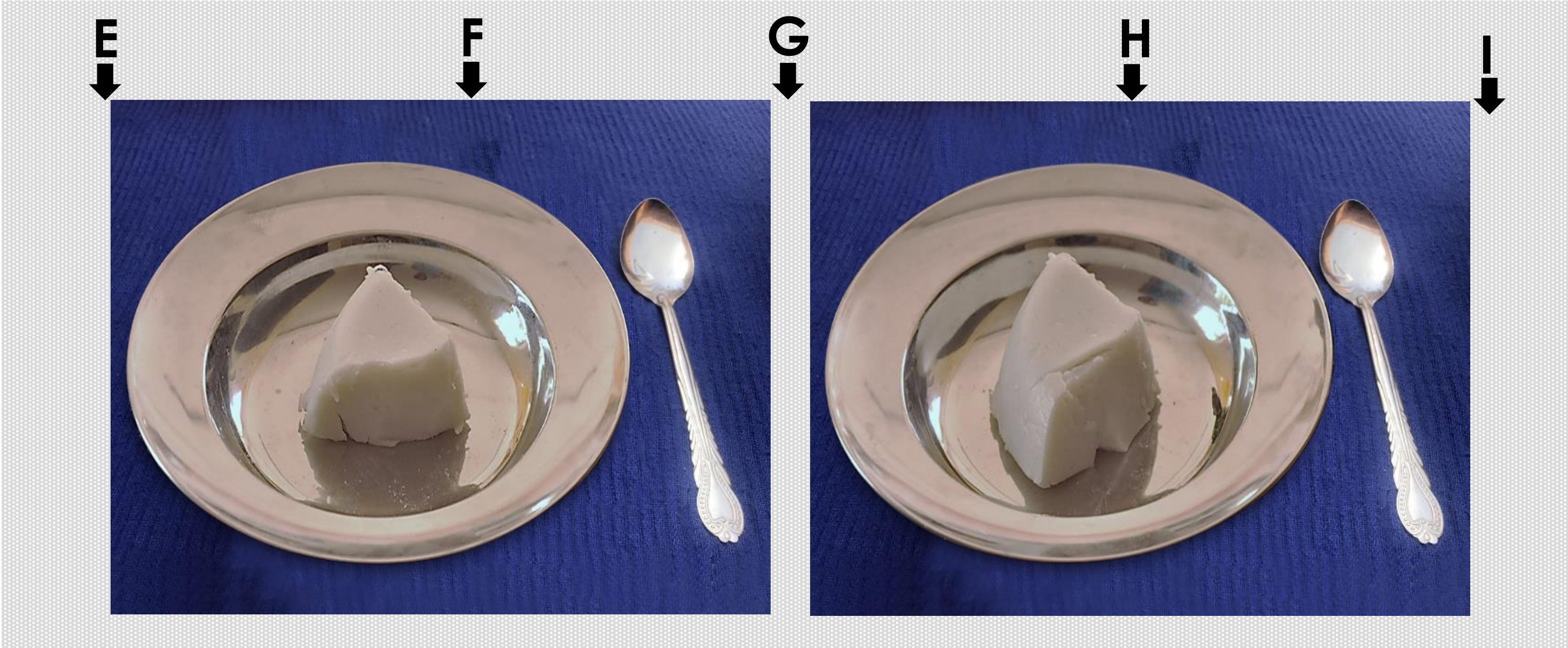
E
F
G
H
I

3 6-11-month – Rice, boiled

A B

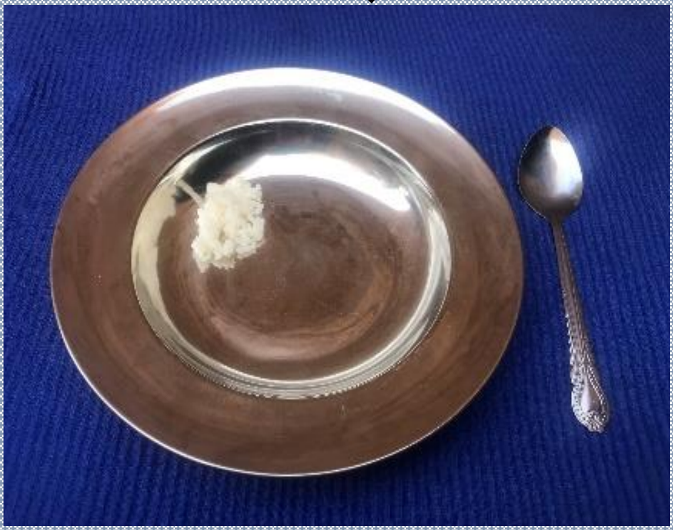

C D

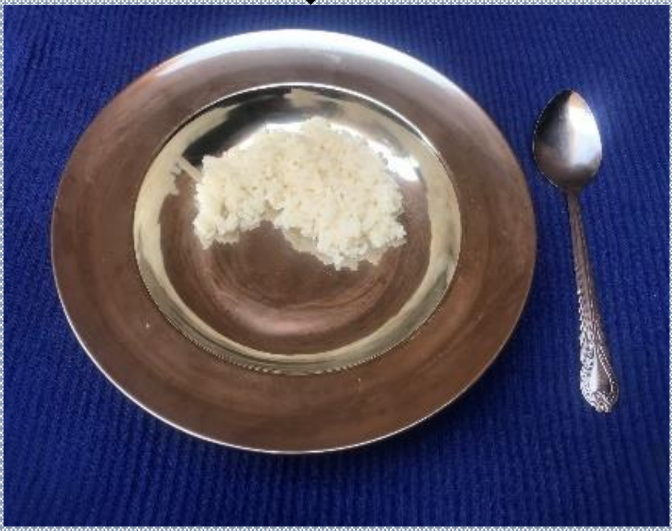

4 6-11-month – Cowpea with rice

A B

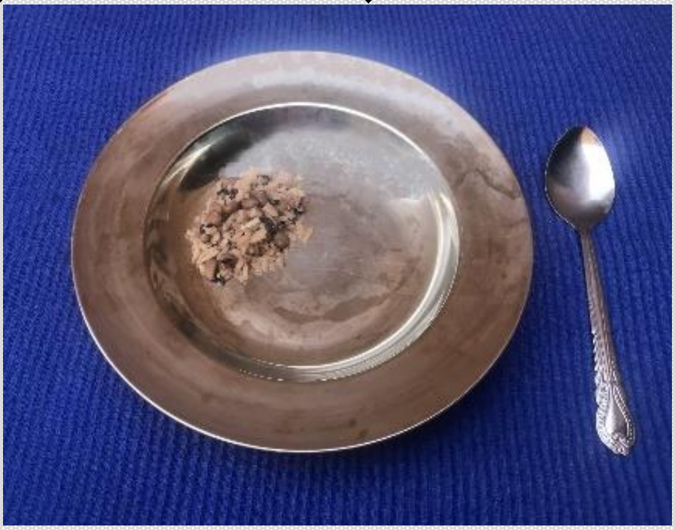

C D

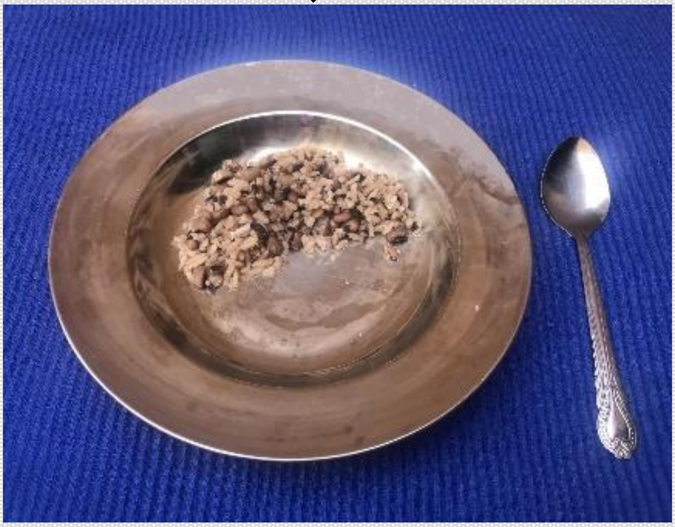

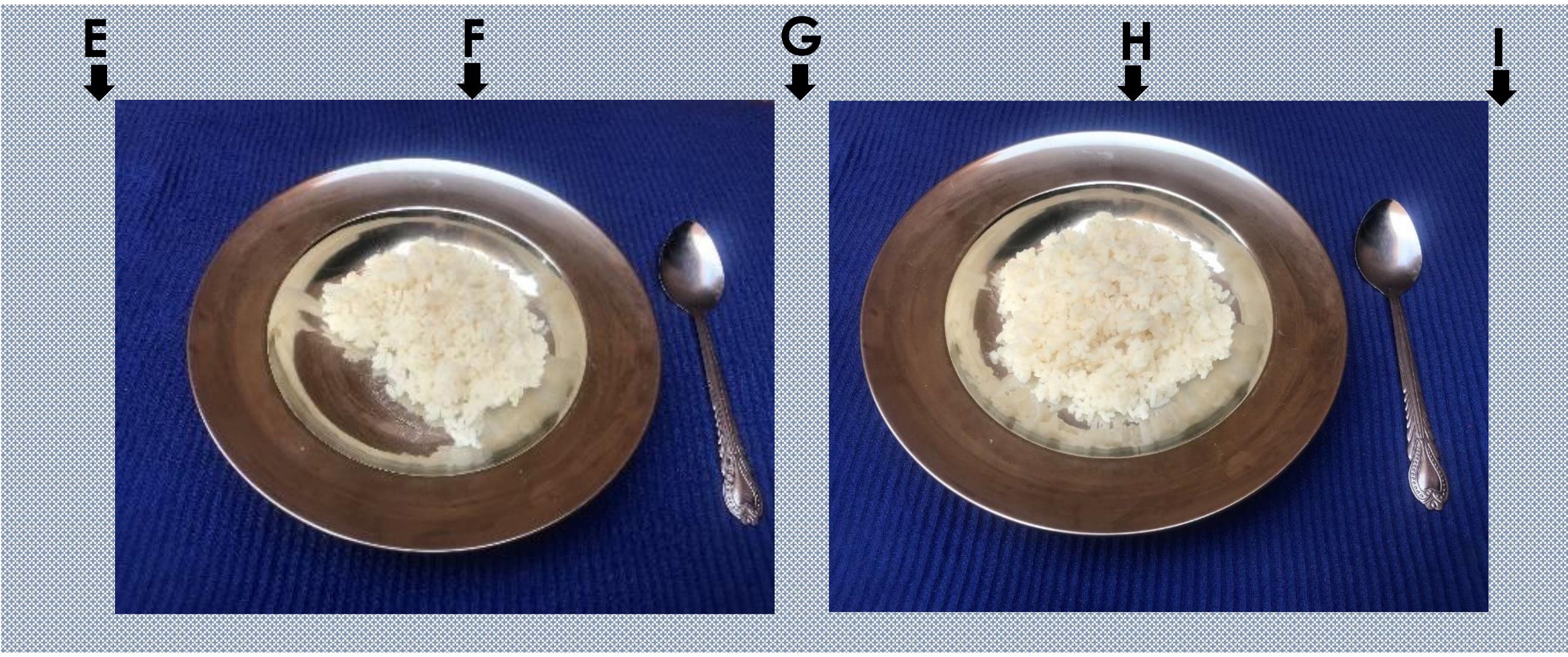
E
F
G
H
I

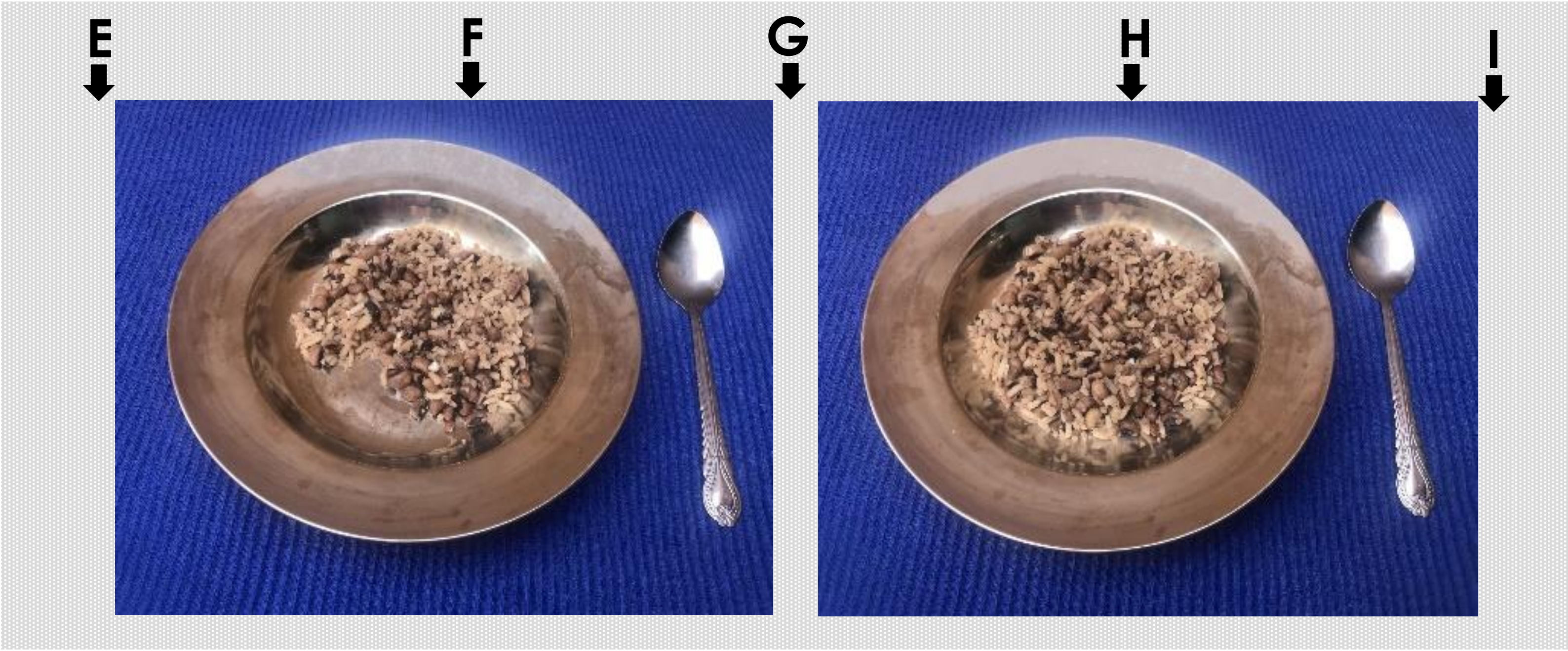
E
F
G
H
I

5 6-11-month – Spaghetti

A B

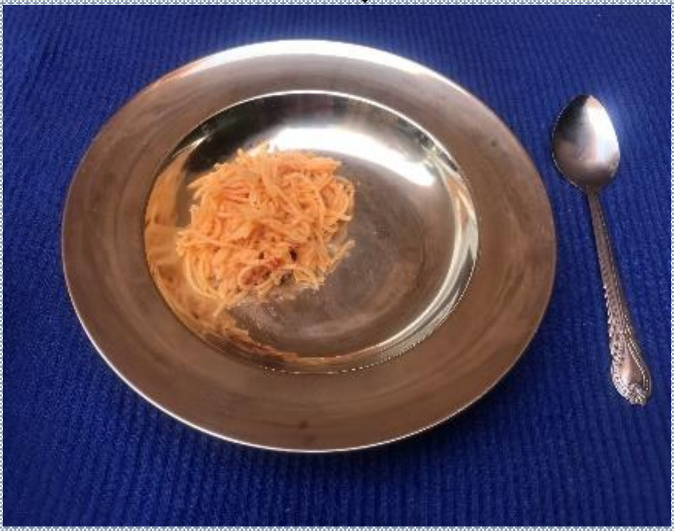

C D

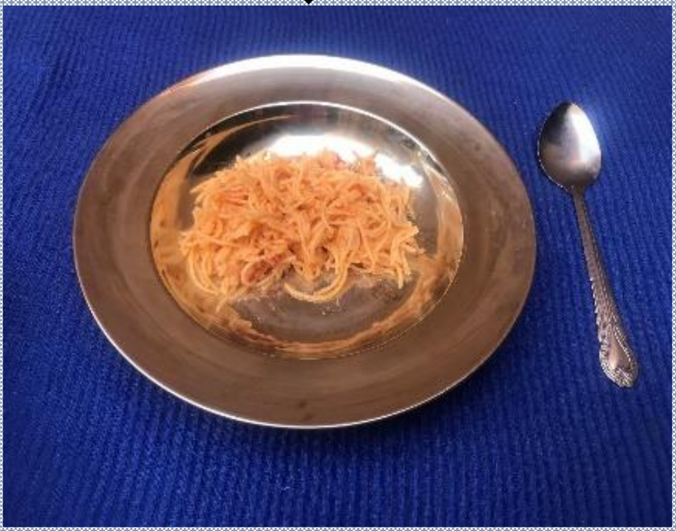

6 6-11-month - Sauce, groundnut paste

A B

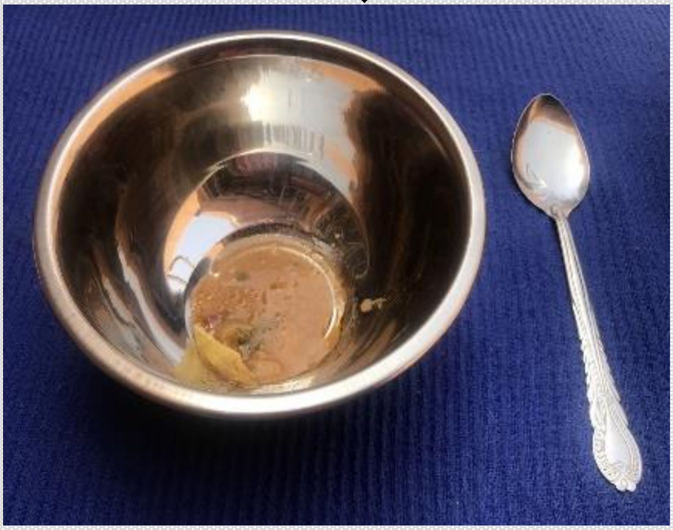

C D

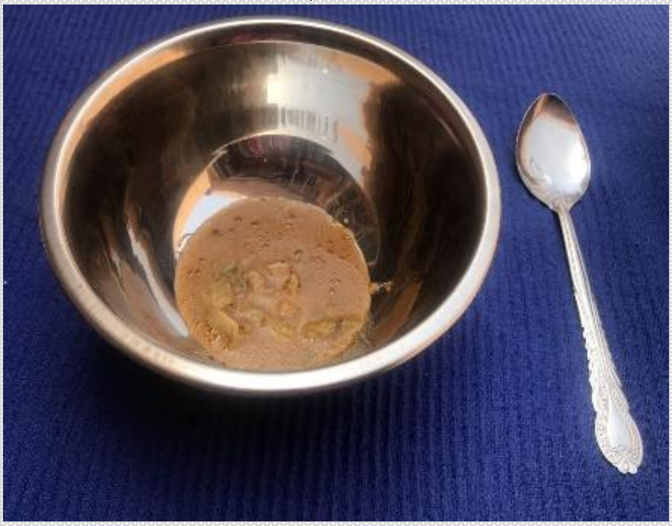

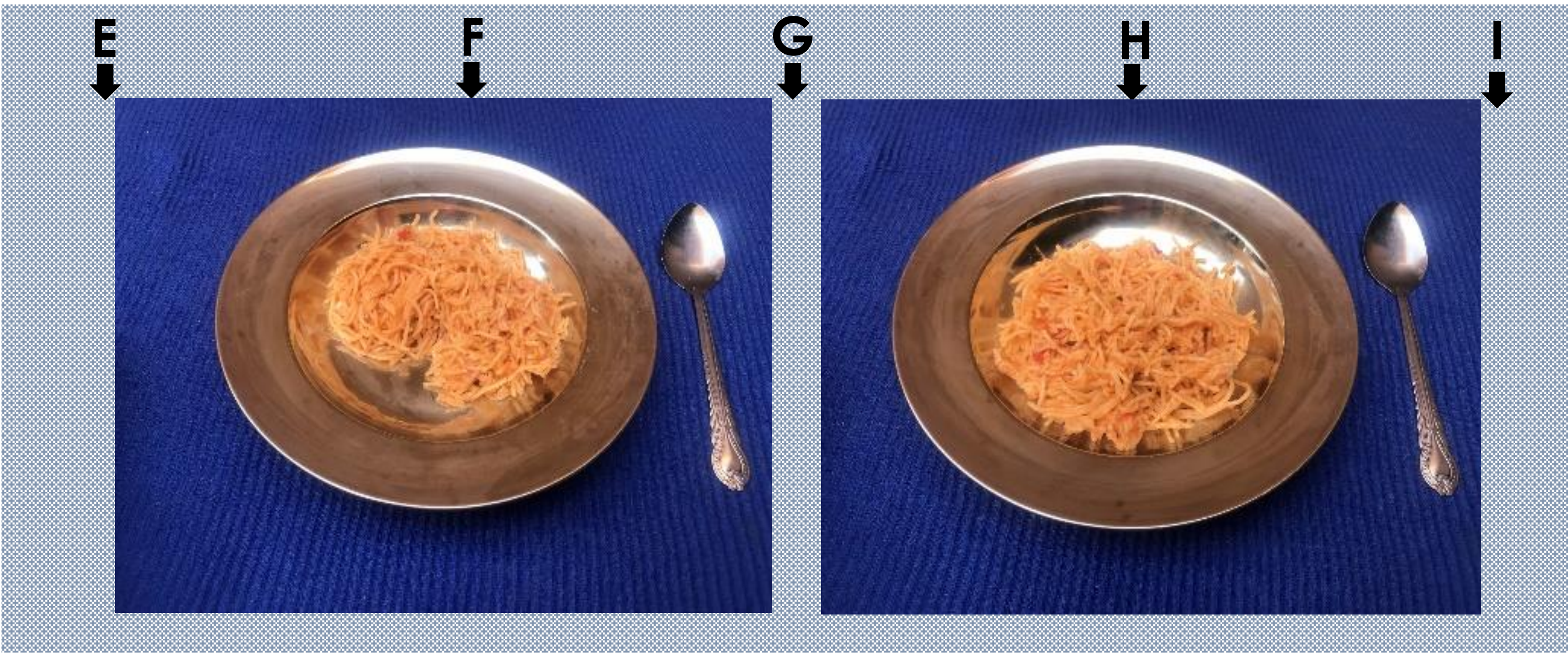
E
F
G
H
I

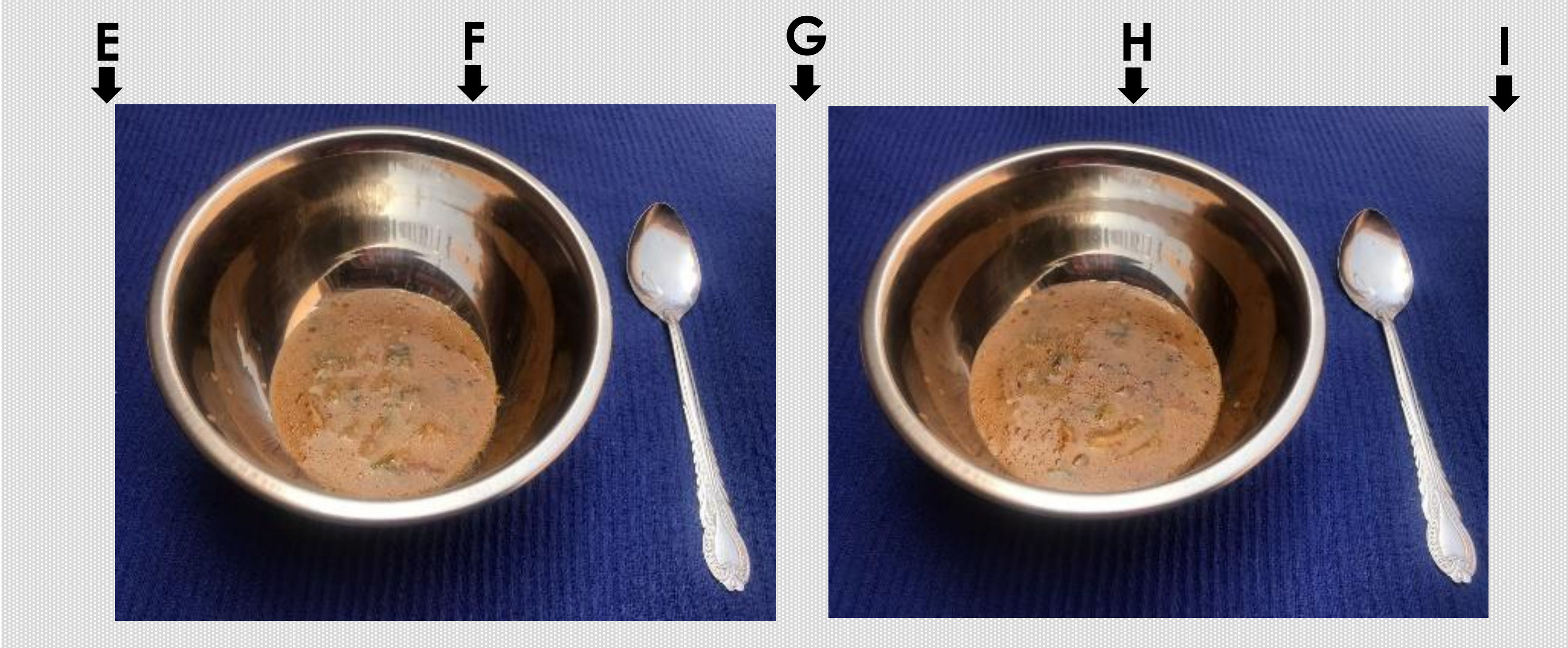
E
F
G
H
I

7 6-11-month - Sauce, vegetables (djabadji)

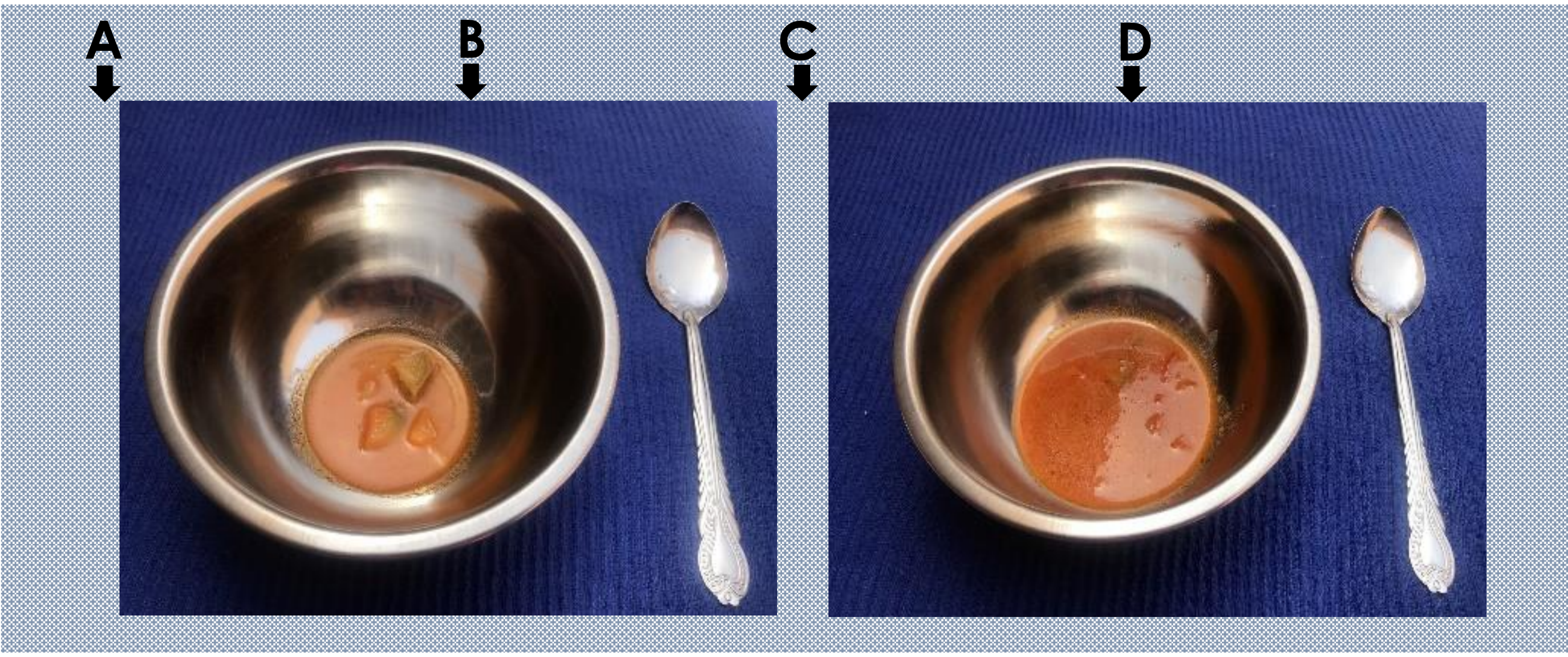

8 6-11-month - Sauce, dry okra

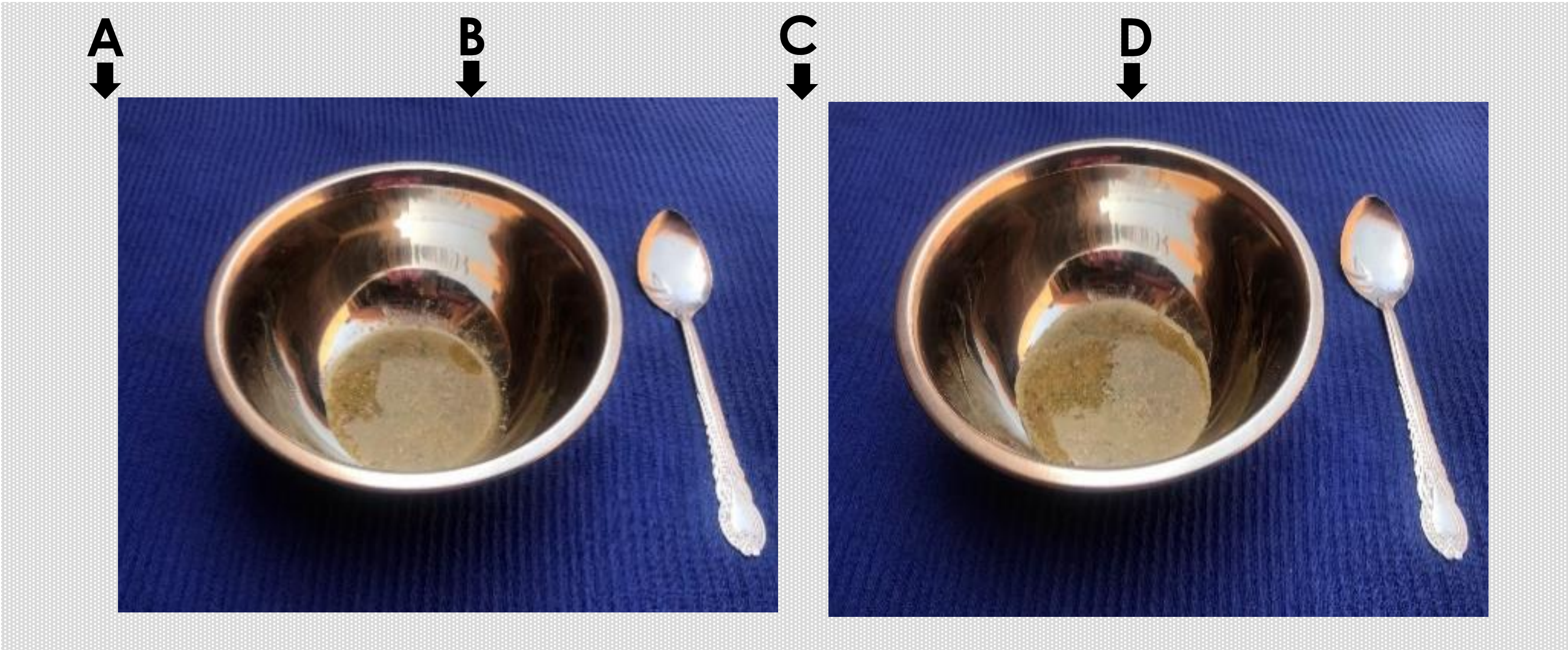

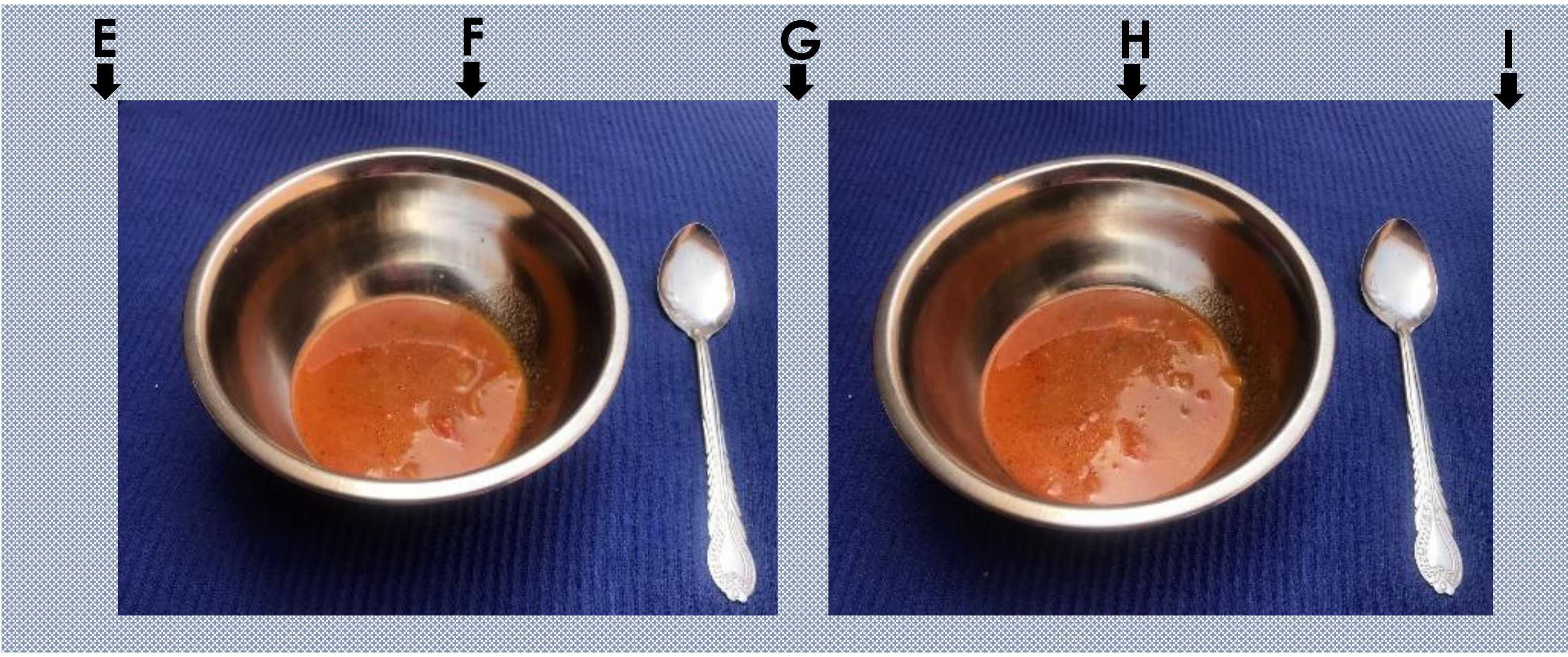
E
F
G
H
I

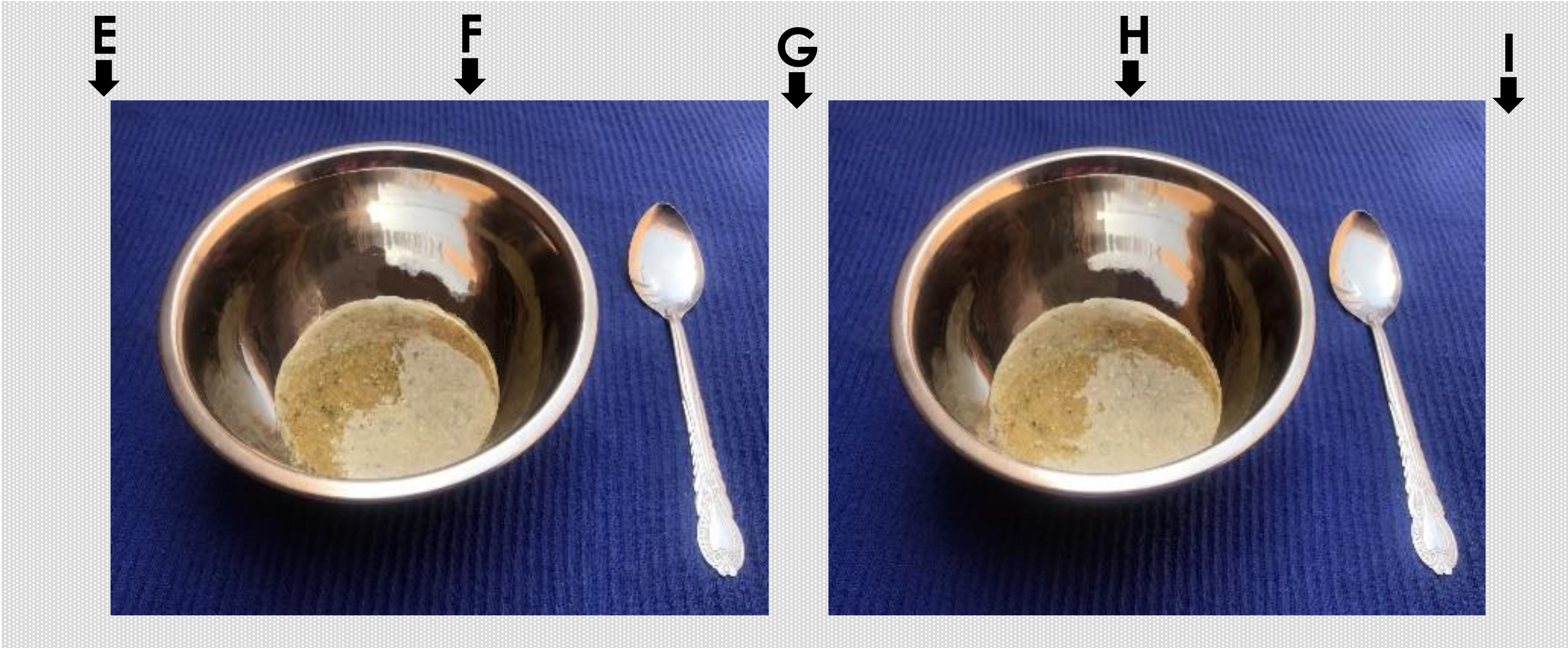
E
F
G
H
I

9 6-11-month - Dish, leafy vegetables

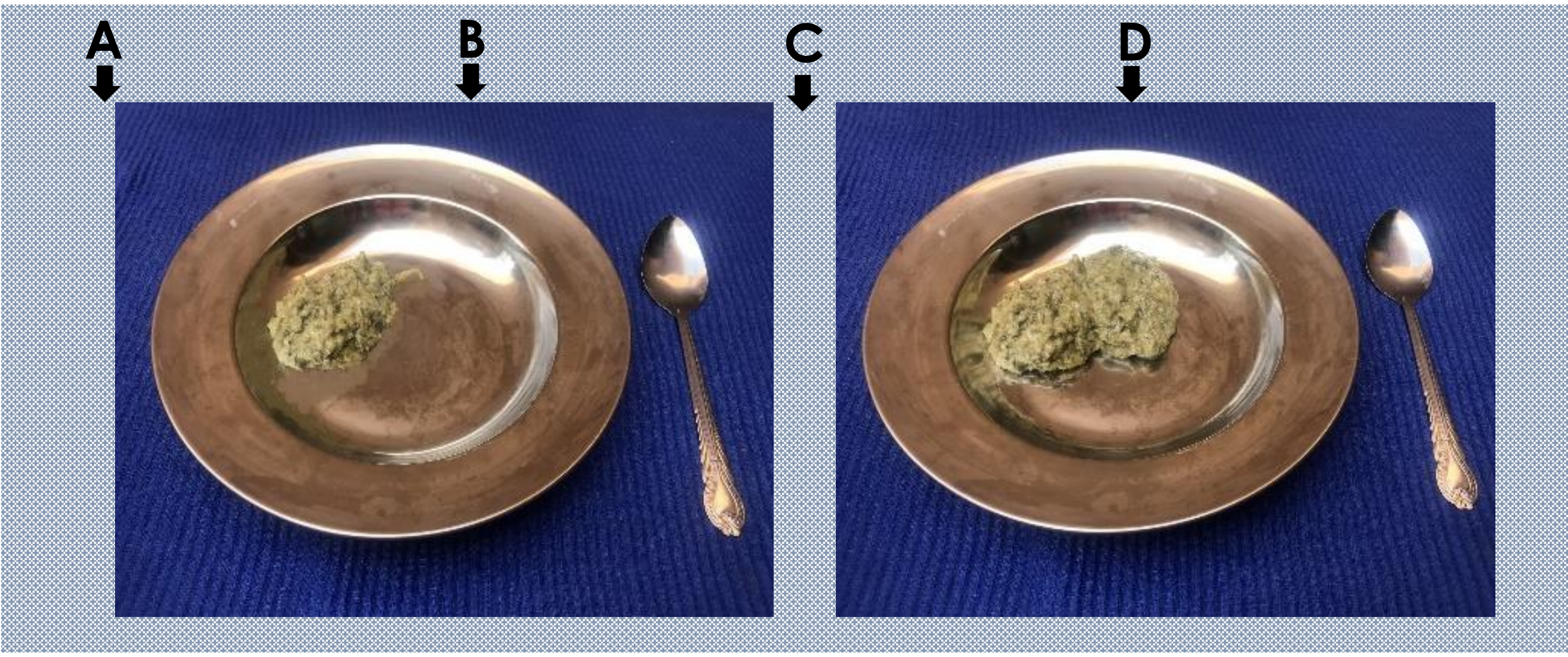

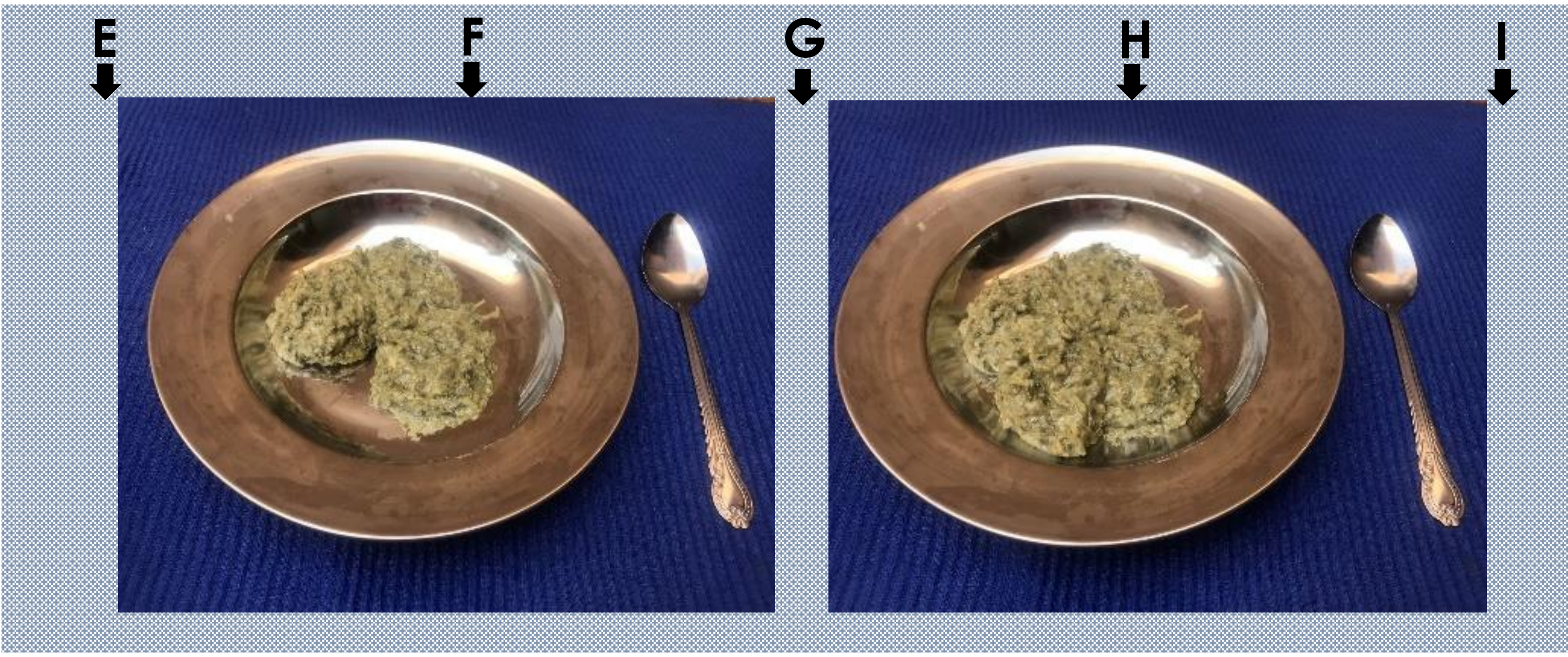
E
F
G
H
I

PART 2:

PORTION SIZES FOR 12-23-MONTH-OLD CHILDREN

10. Fermented millet porridge -------------------------------- 1
11. Stiff corn porridge-- 1
12. Rice, boiled -- 1
13. Cowpea with rice-- 1
14. Spaghetti -- 19
15. Sauce, groundnut paste ---------------------------------- 1
16. Sauce, vegetables (djabadji) ------------------------- 21
17. Sauce, dry okra--- 21
18. Dish, leafy vegetables (babenda) -------------------- 23
19. Pieces of meat ---23
20. Fish --25

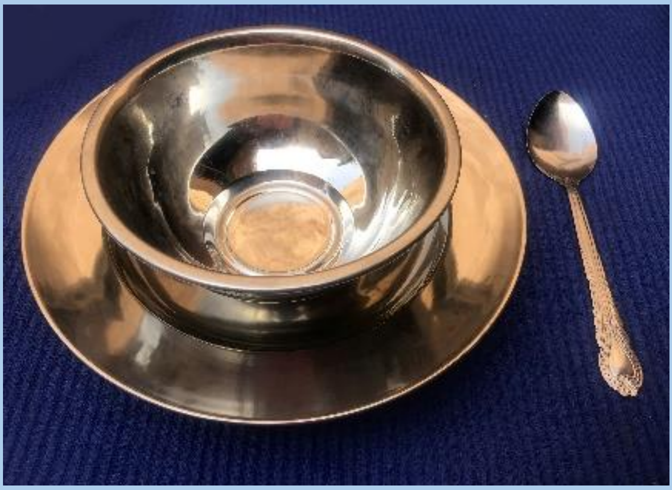

10 12-23-month – Fermented millet porridge

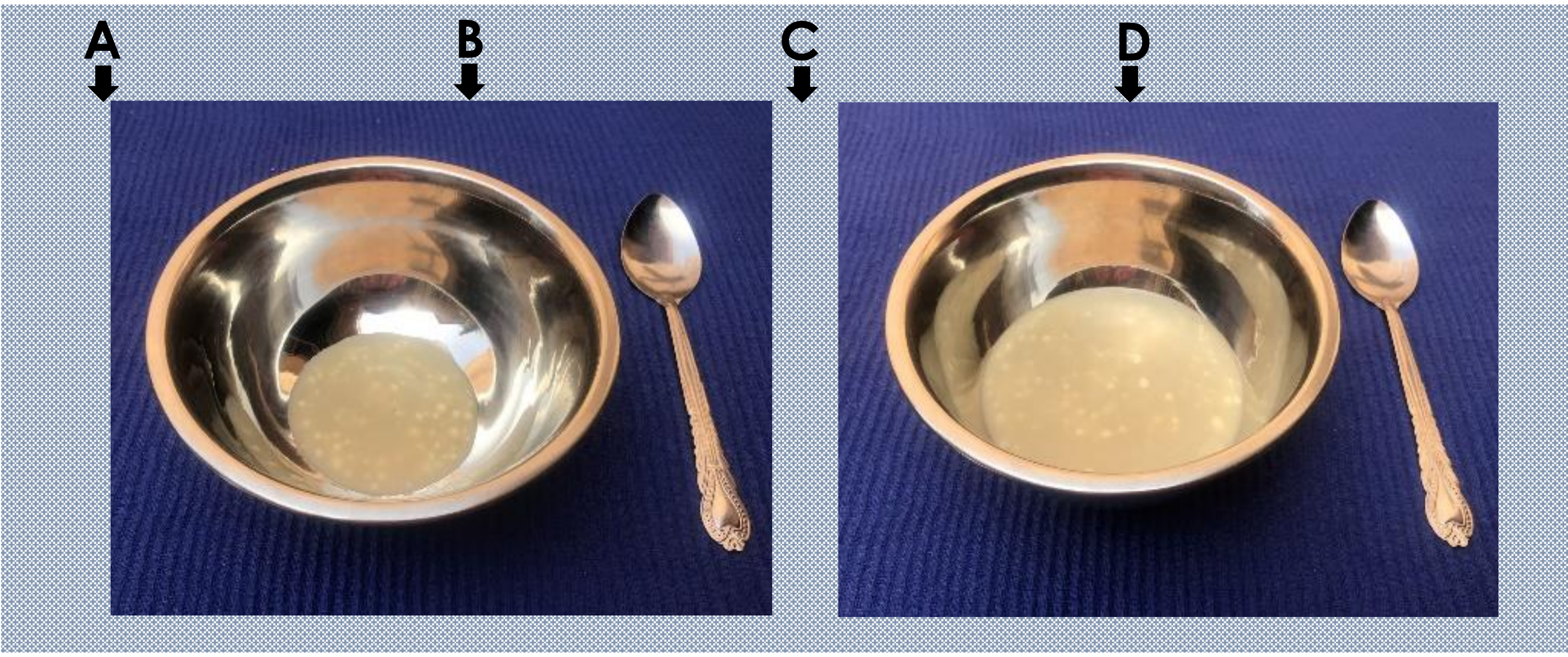

11 12-23-month – Stiff corn porridge

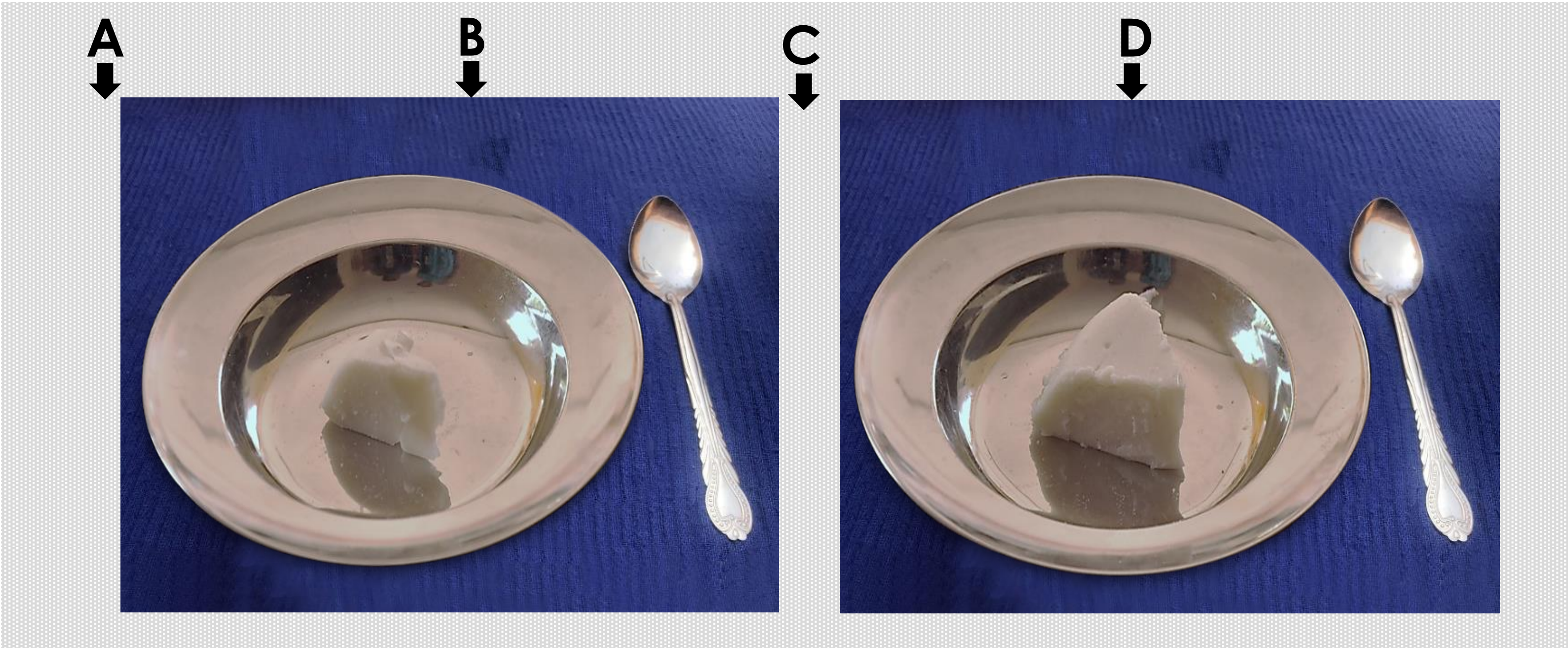

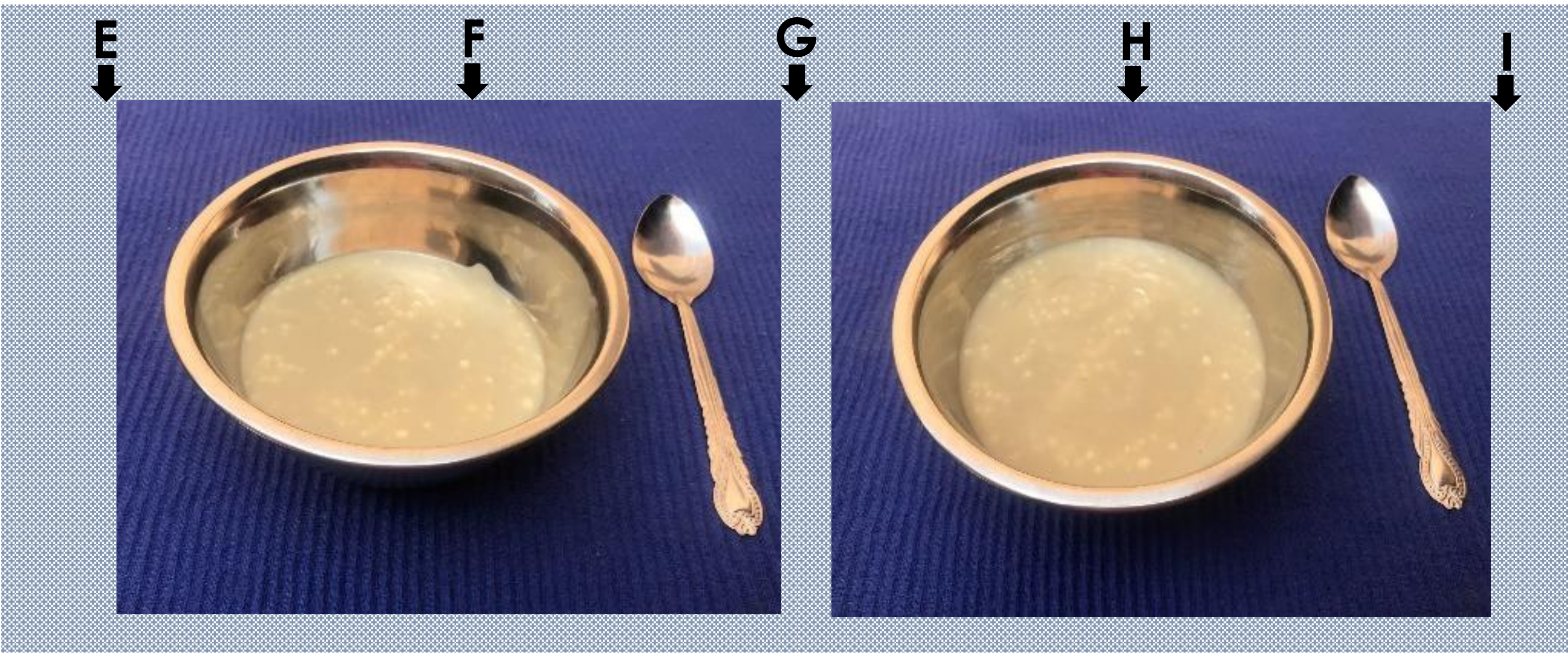
E
F
G
H
I

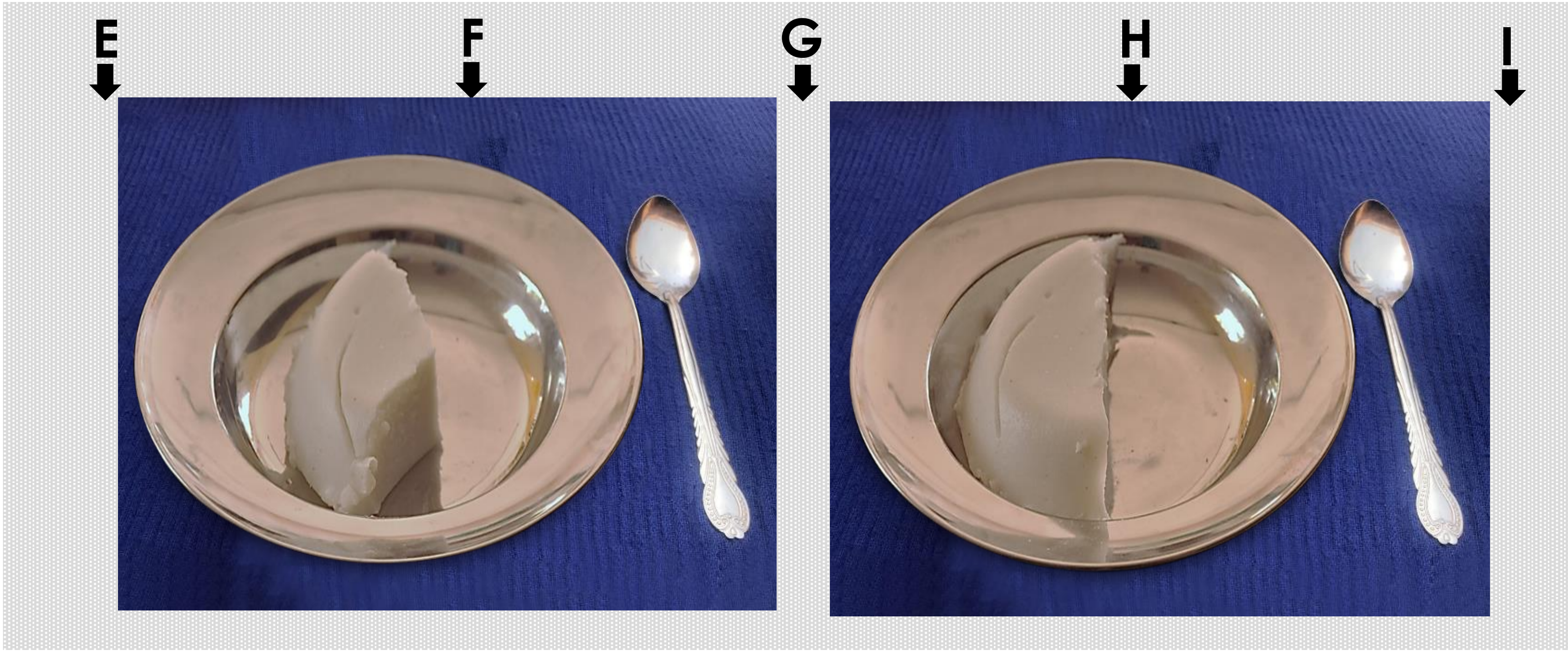
E
F
G
H
I

12 12-23-month – Rice, boiled

A B

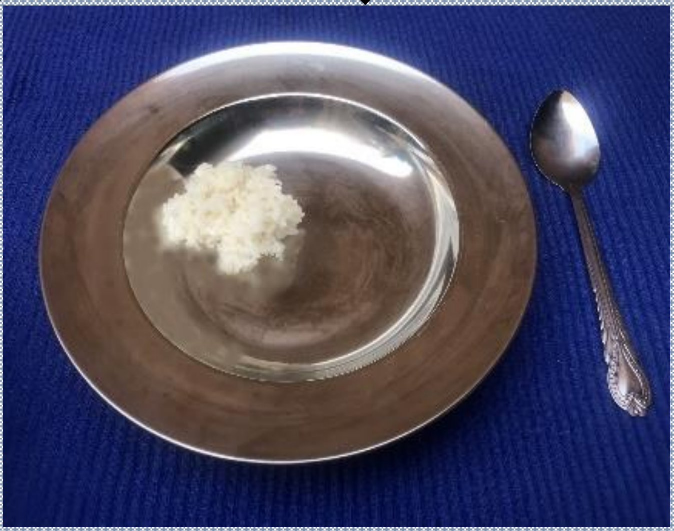

C D

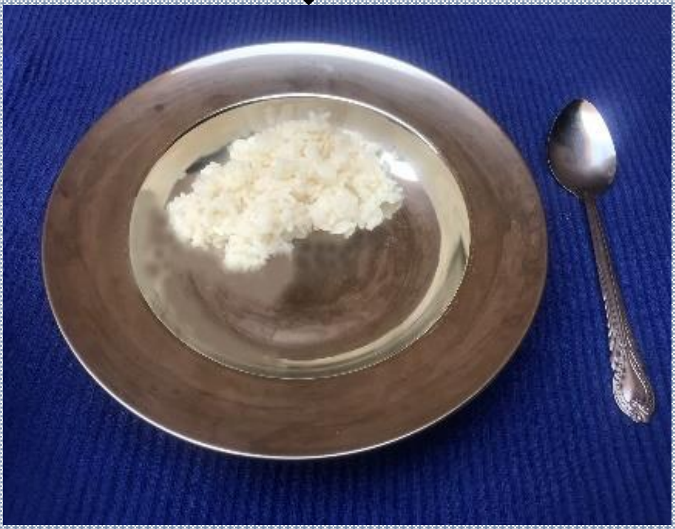

13 12-23-month – Cowpea with rice

A B

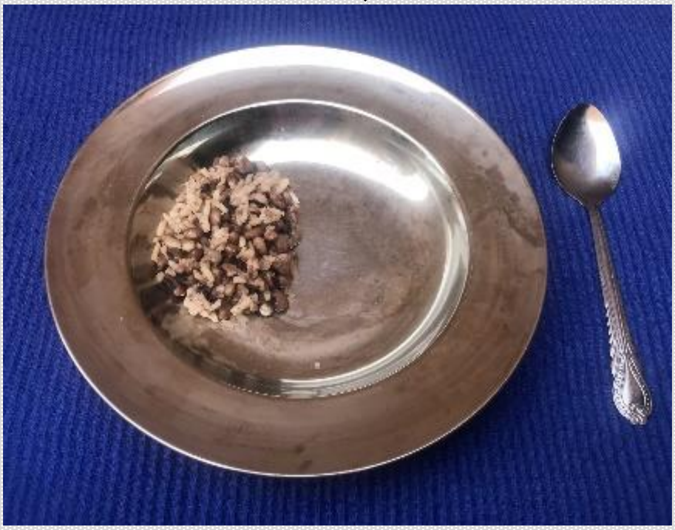

C D

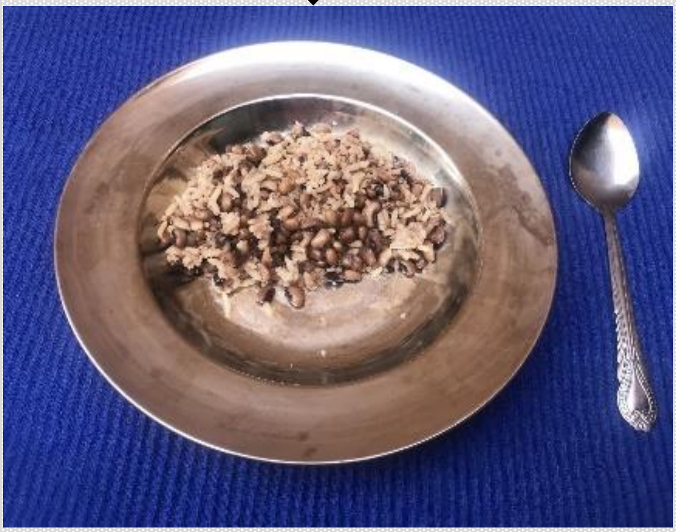

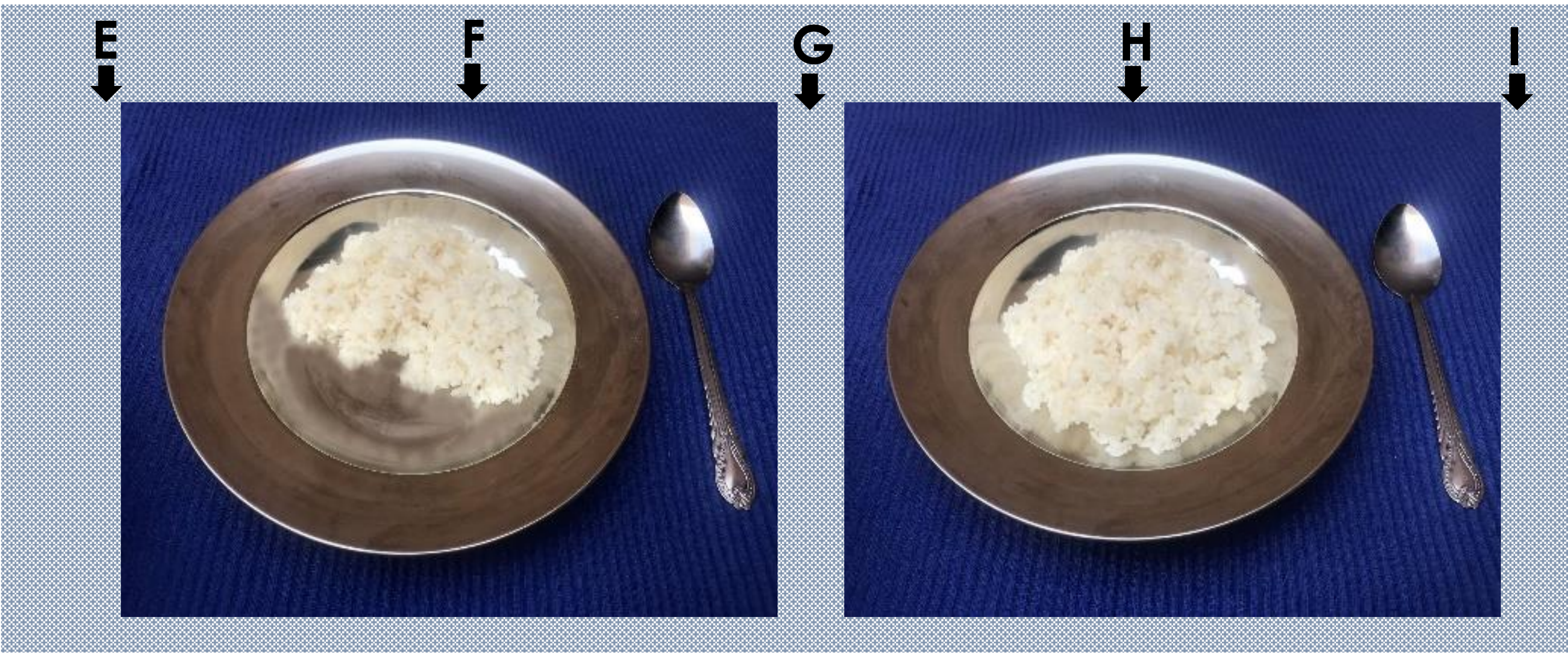
E
F
G
H
I

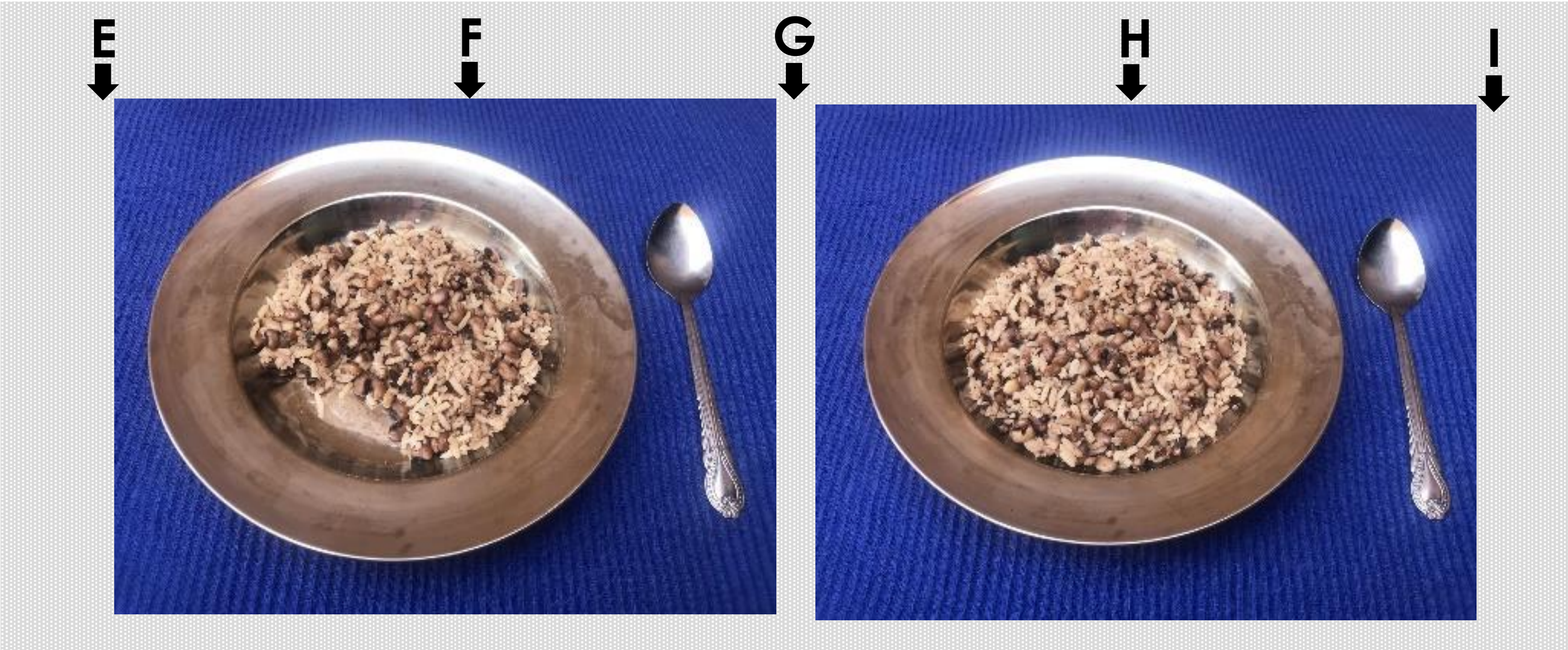
E
F
G
H
I

14 12-23-month – Spaghetti

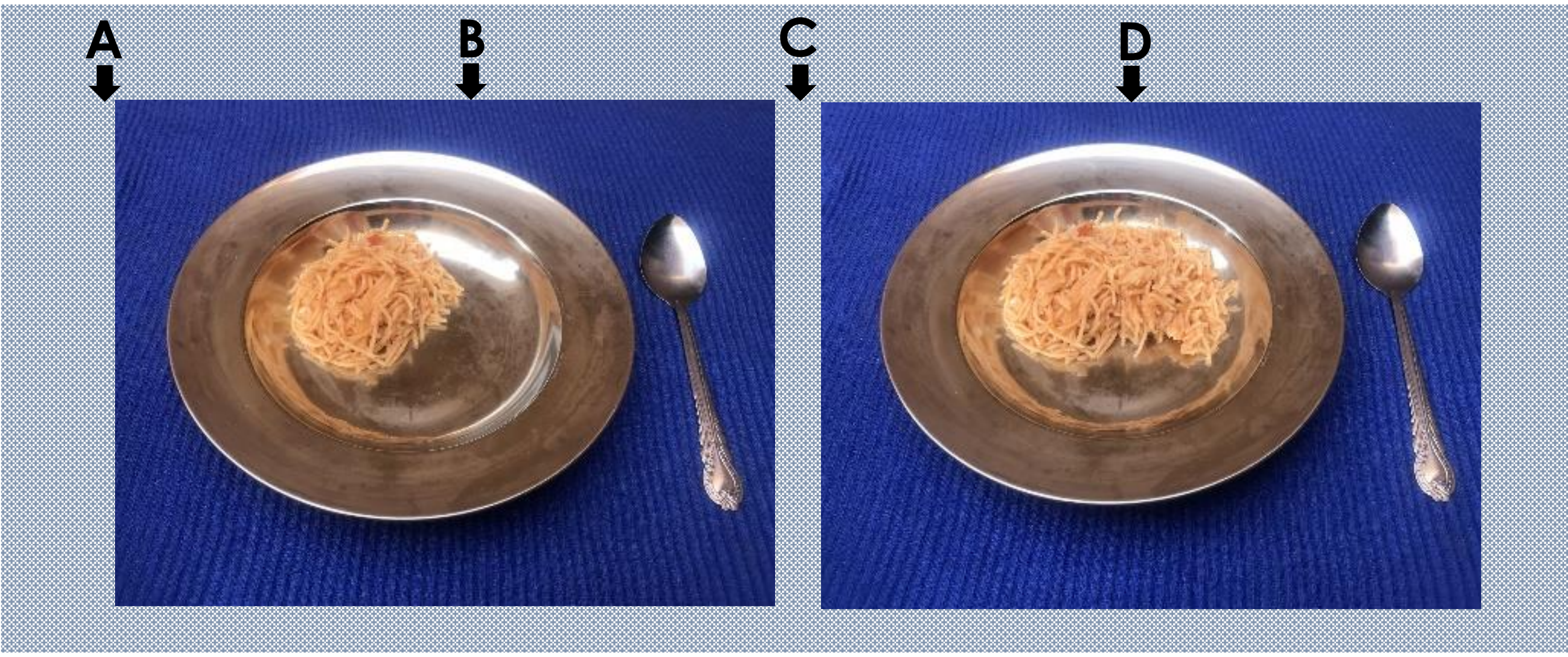

15 12-23-month - Sauce, groundnut paste

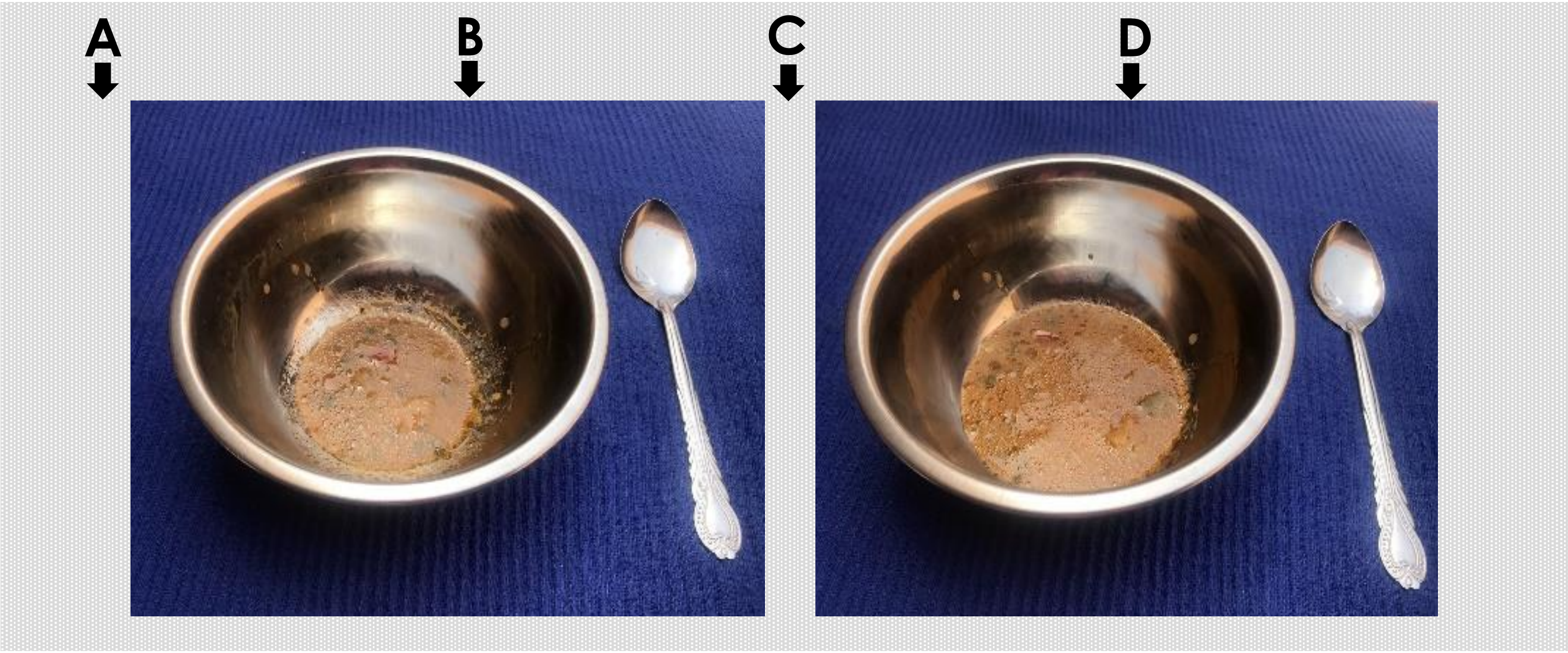

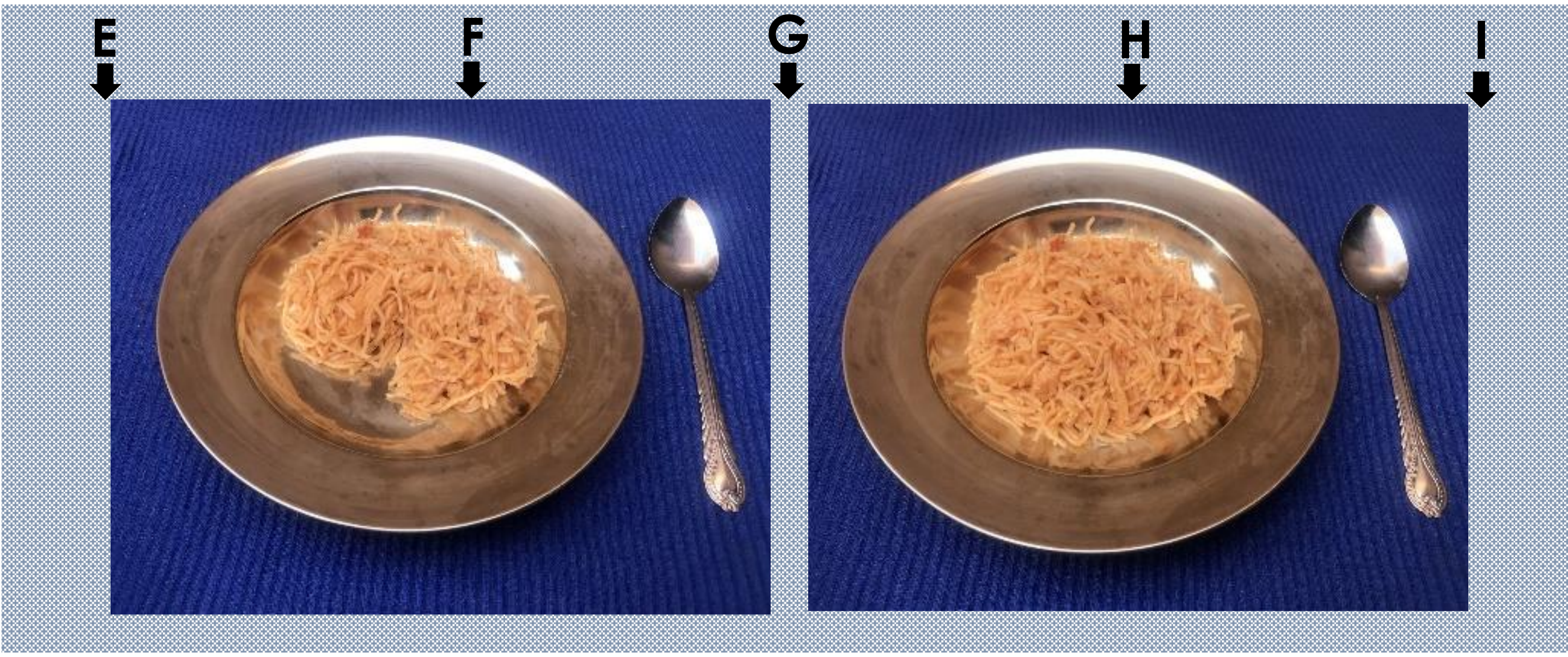
E
F
G
H
I

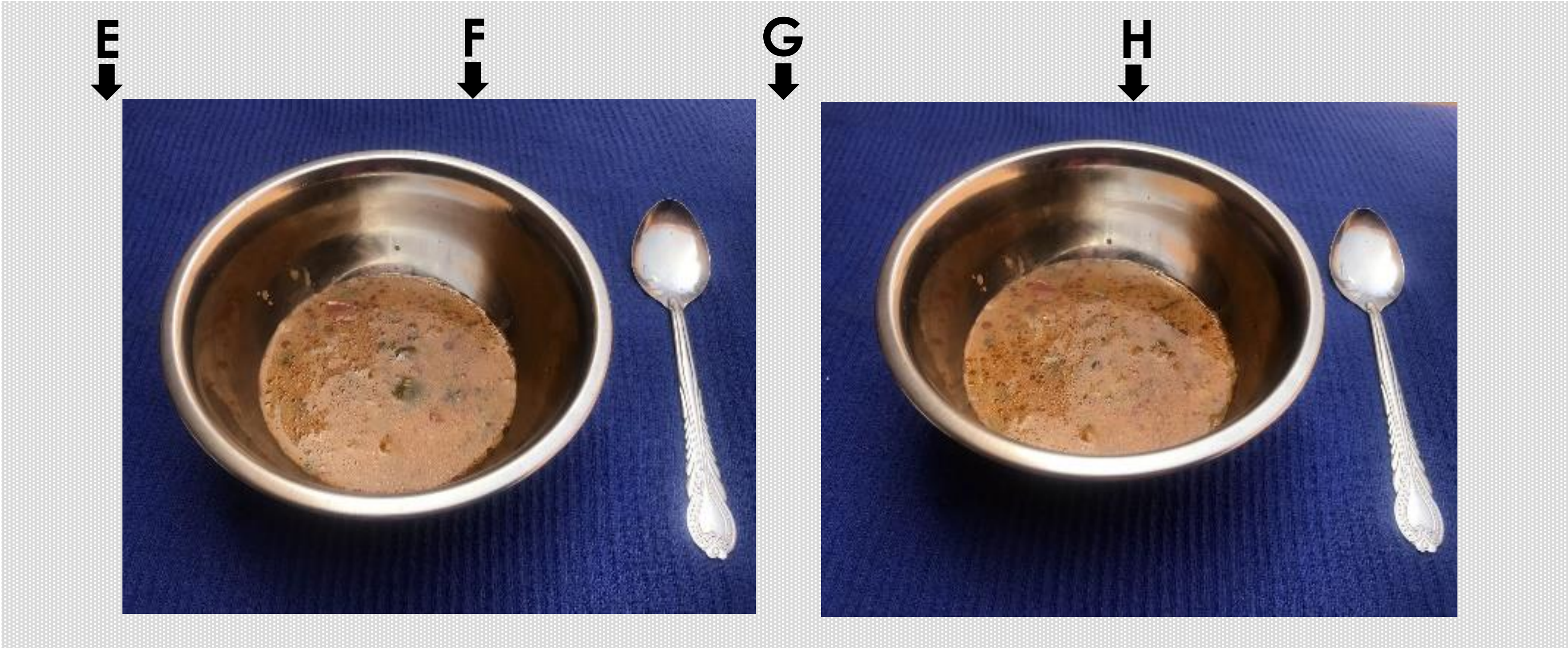
E
F
G
H

16 12-23-month - Sauce, vegetables (djabadji)

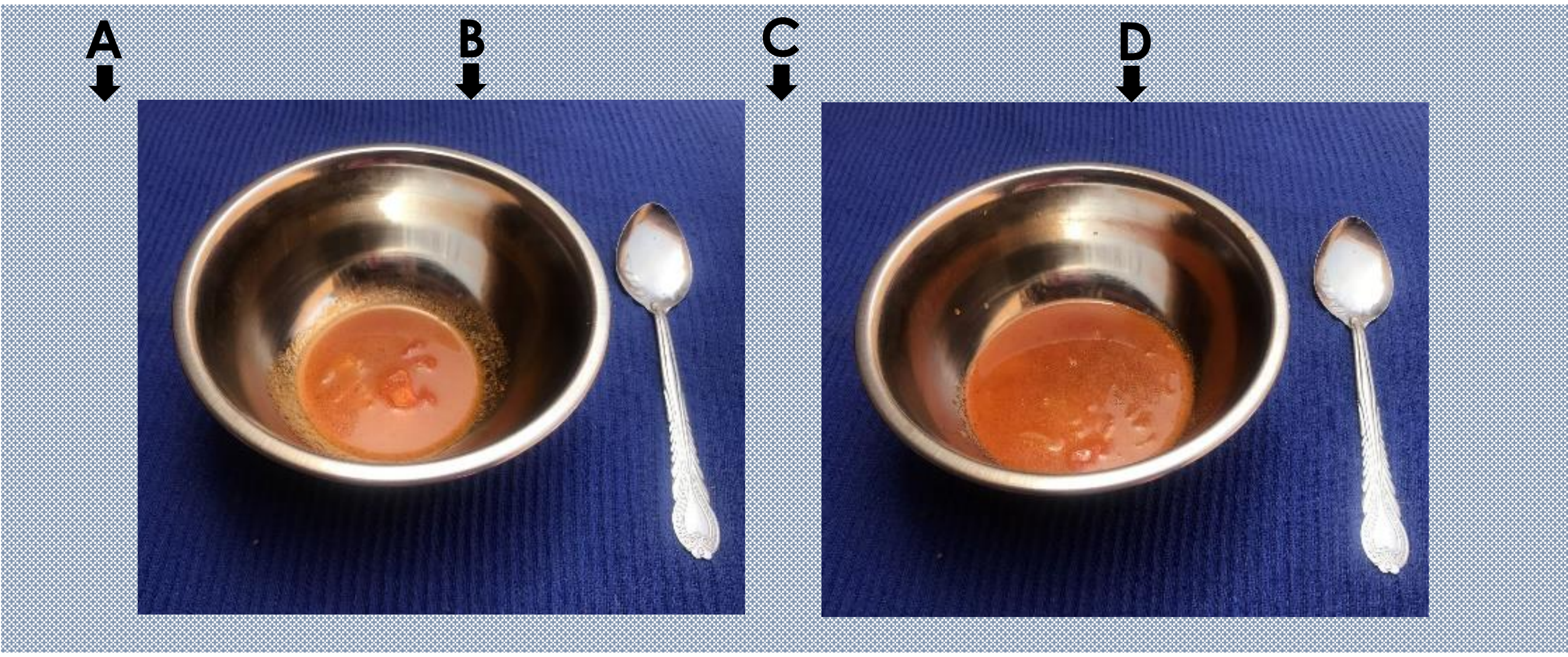

17 12-23-month - Sauce, dry okra

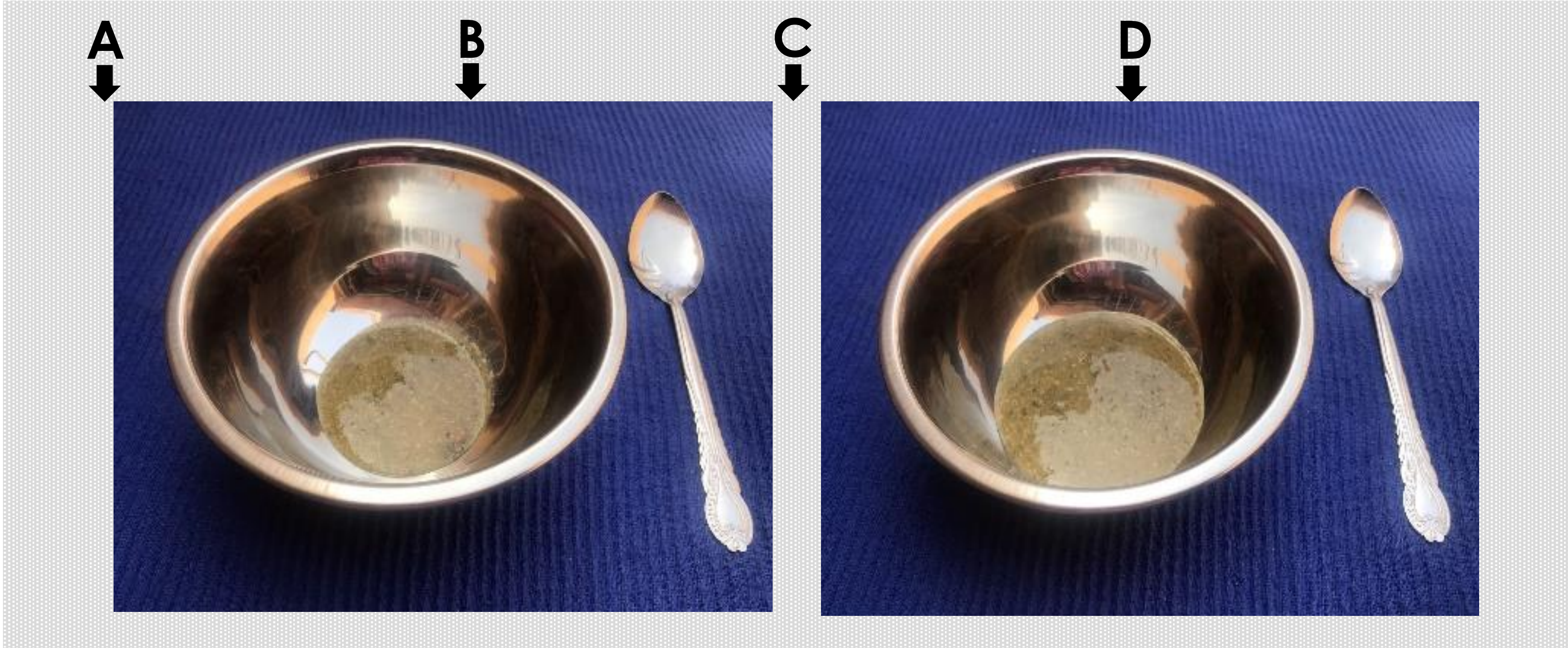

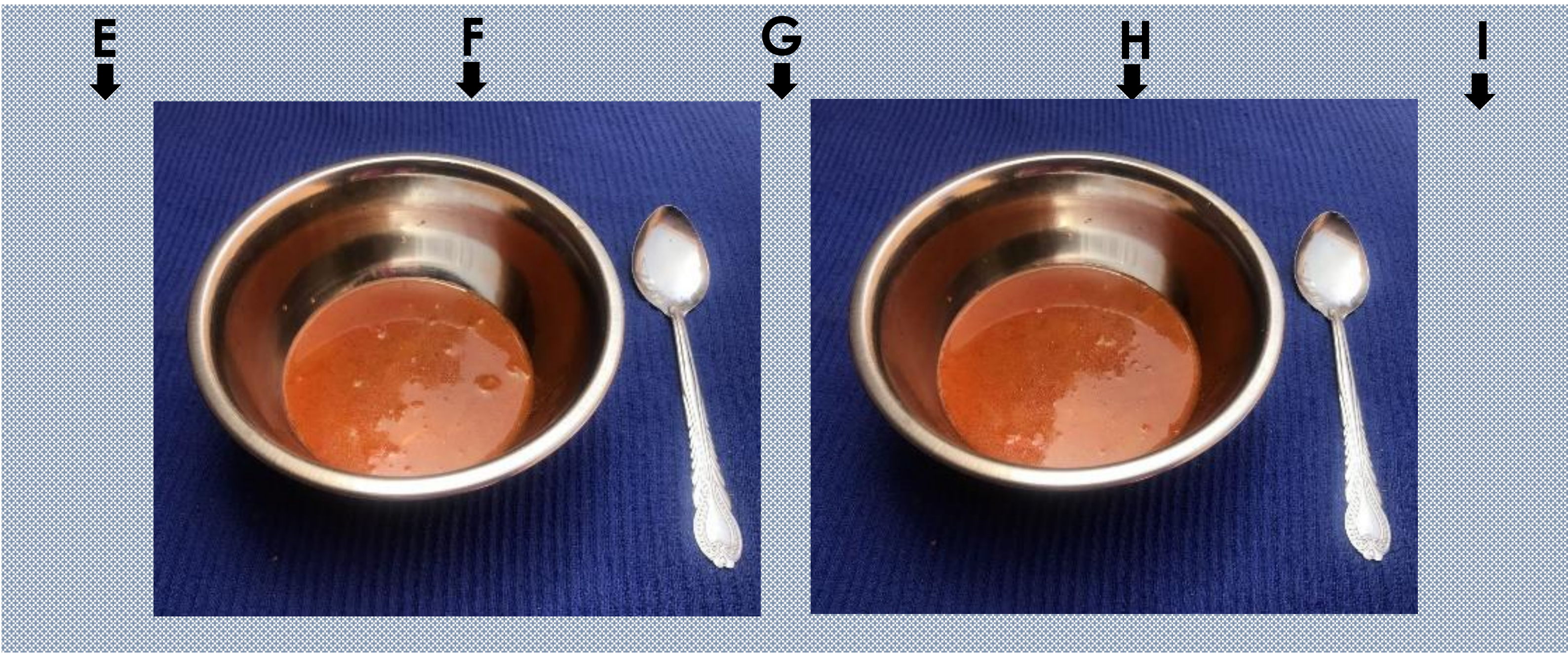
E
F
G
H
I

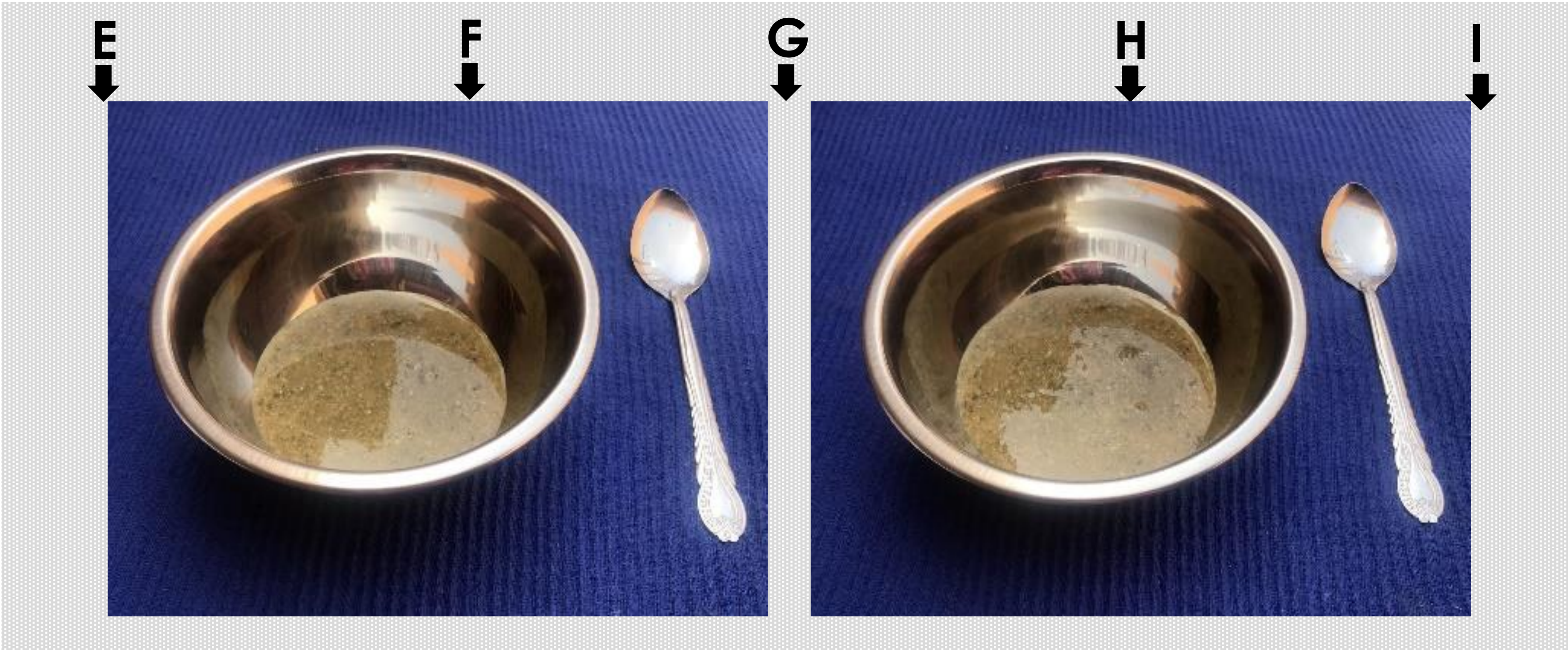
E
F
G
H
I

18 12-23-month - Dish, leafy vegetables

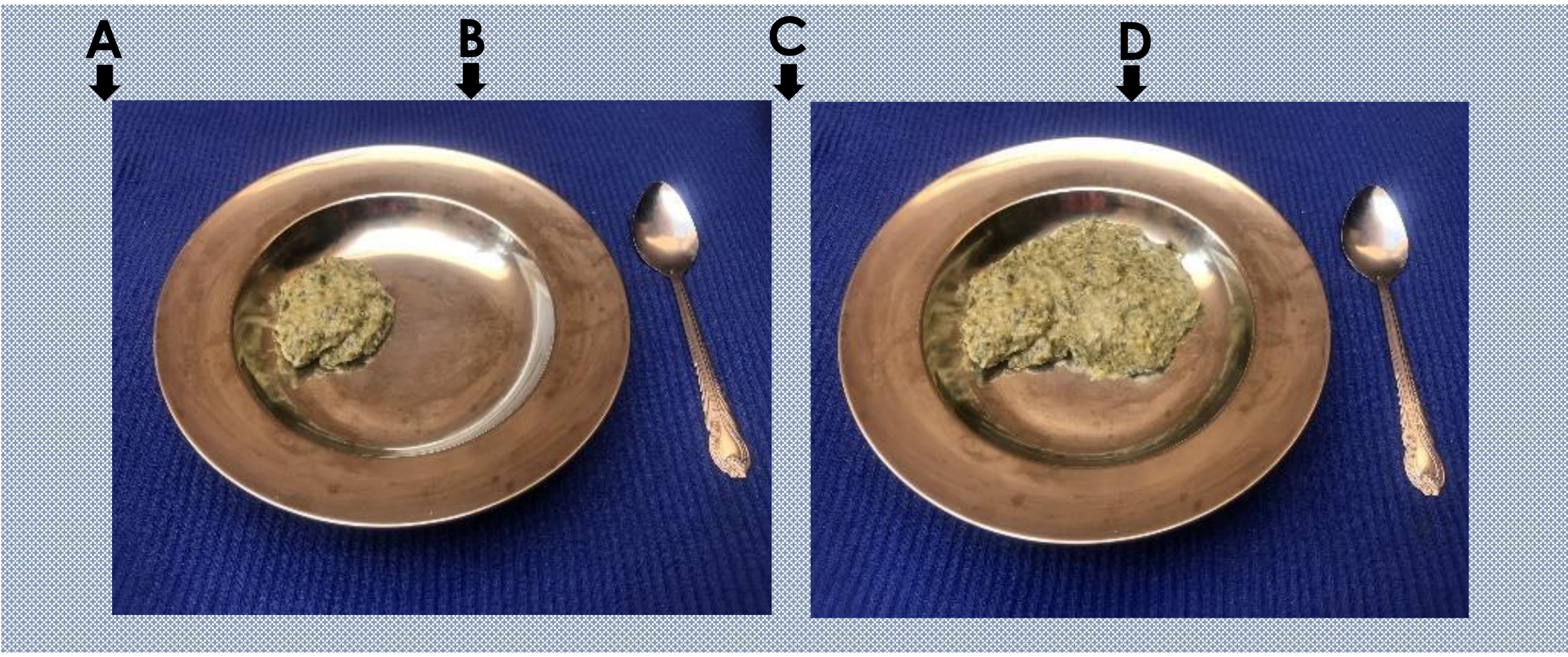

19 12-23-month – Pieces of meat

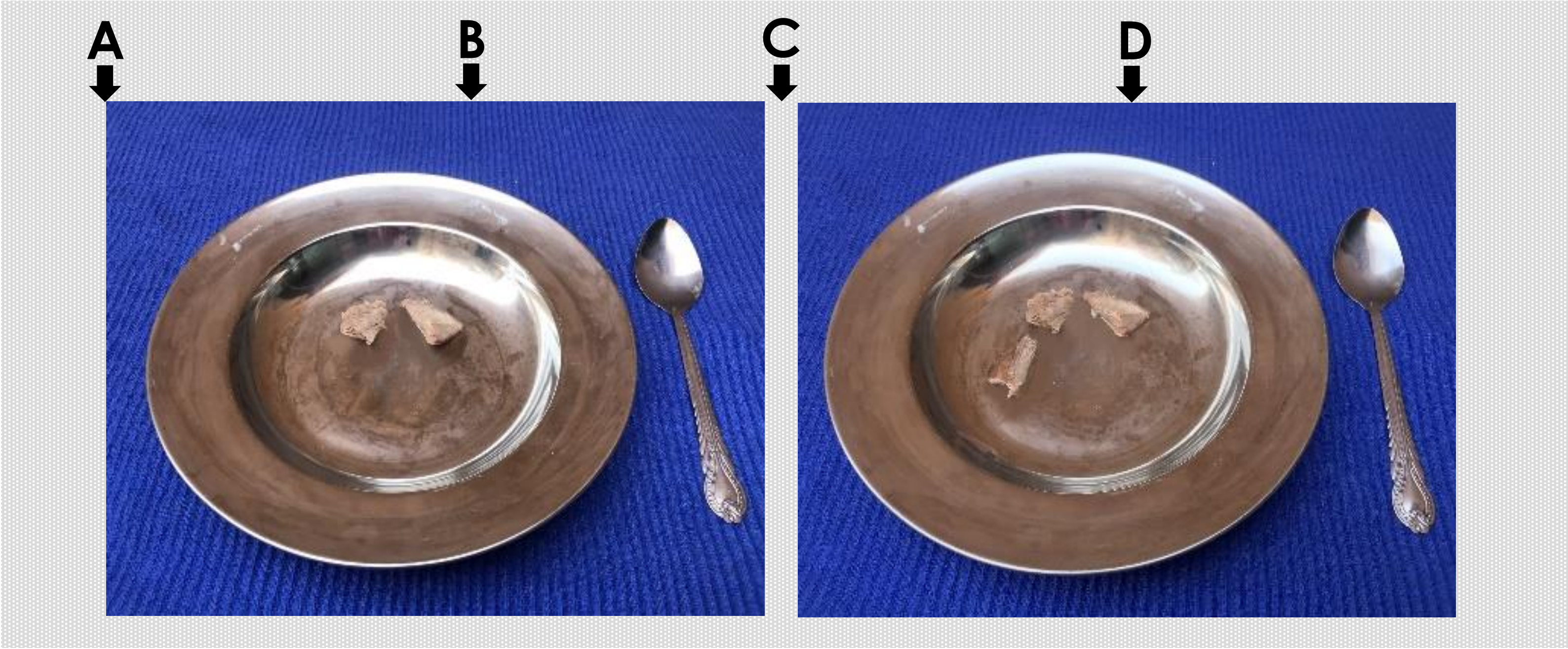

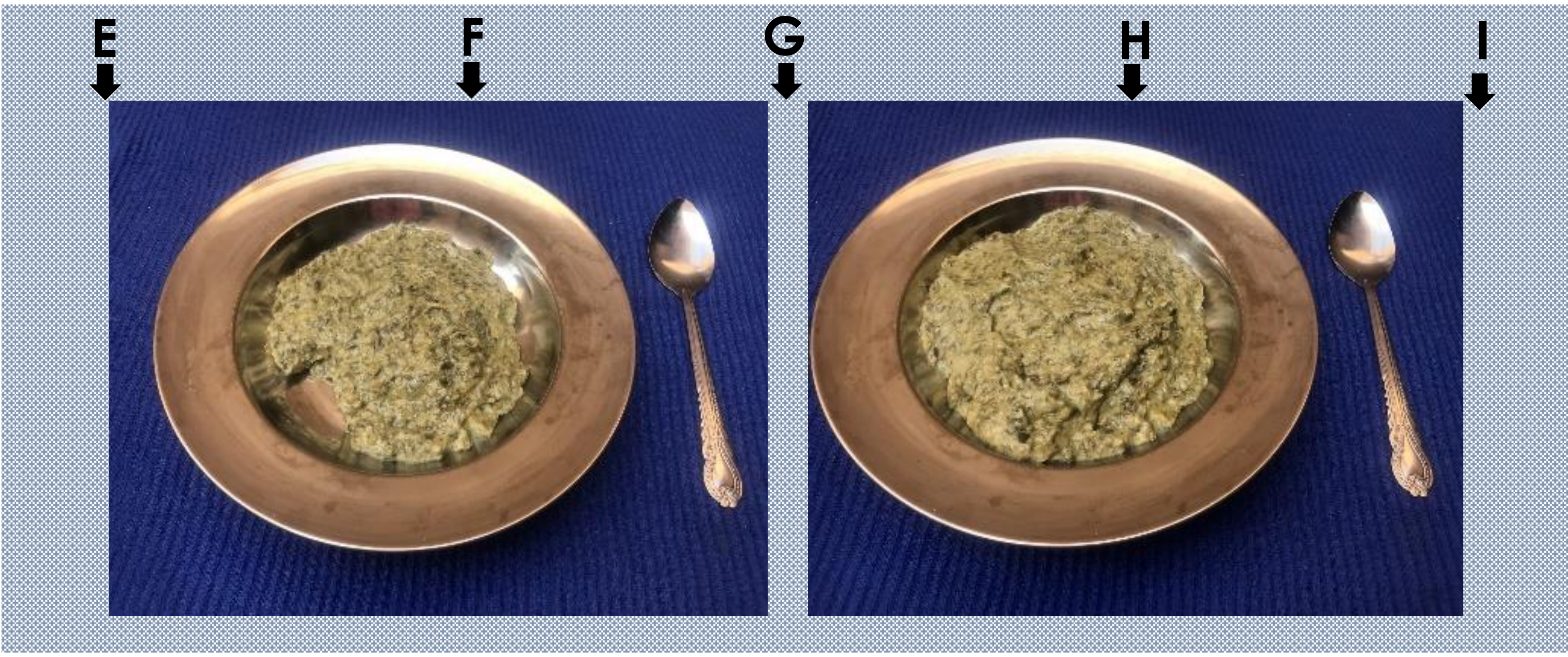
E
F
G
H
I

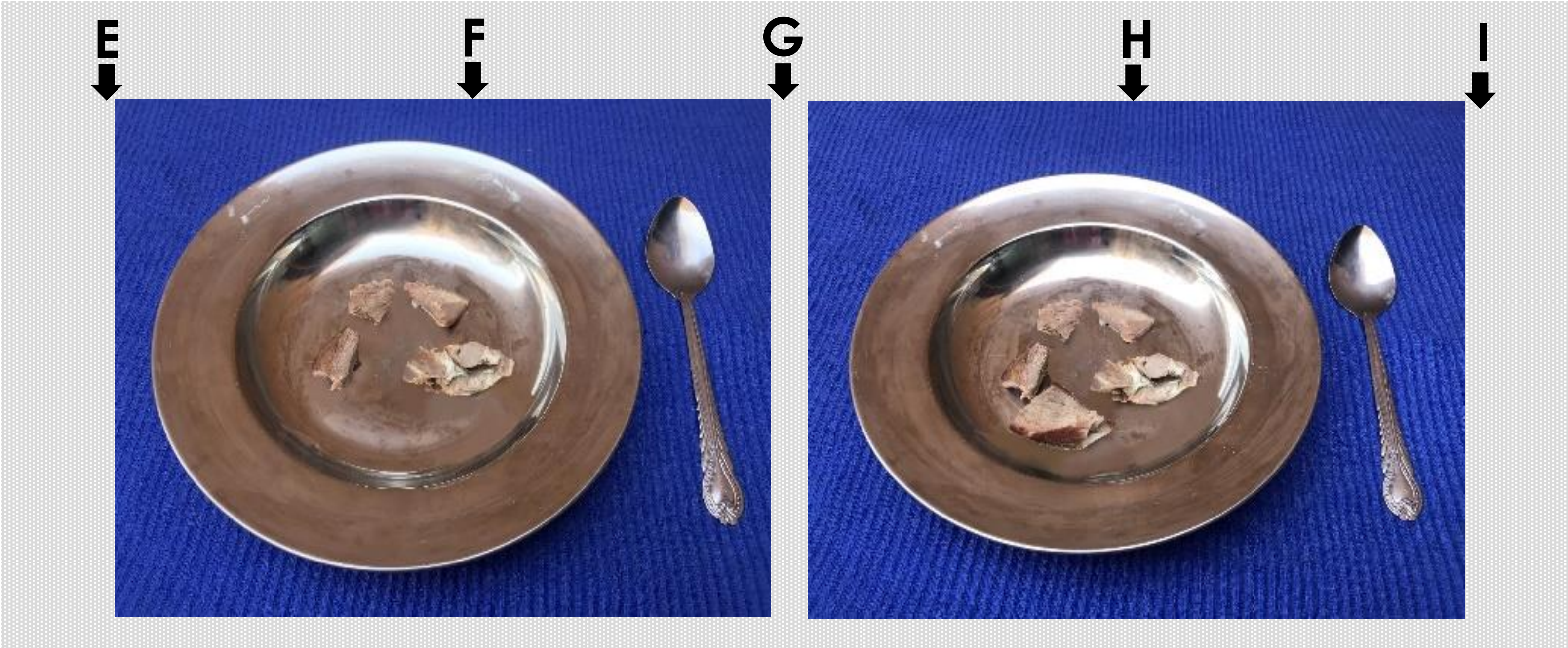
E
F
G
H
I

20 12-23-month – Fish

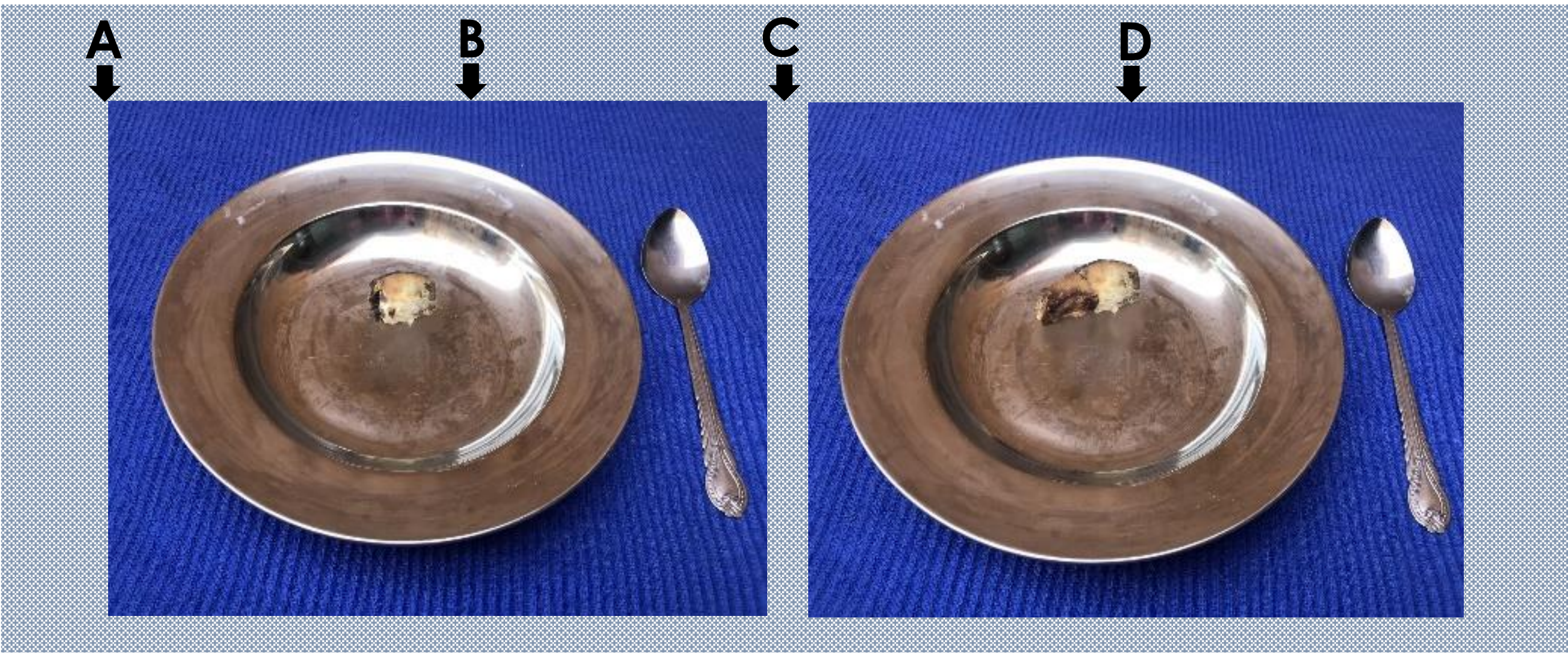

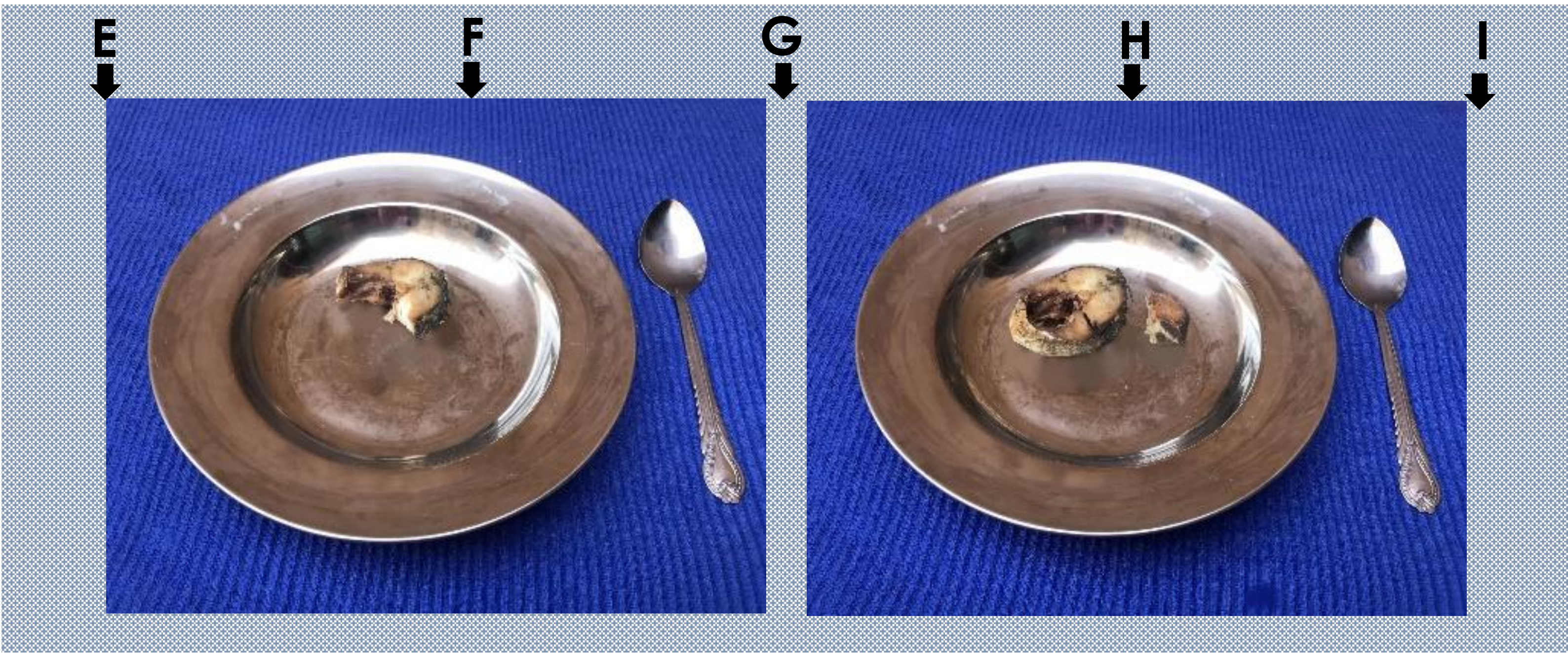
E
F
G
H
I

PART 3 :

PORTION SIZES FOR WOMEN OF CHILDBEARING AGE (WCA)

21. Fermented millet porridge--------------------------------29
22. Stiff corn porridge --29
23. Rice, boiled---31
24. Cowpea with rice ---31
25.Spaghetti --33
26. Sauce, groundnut paste----------------------------------33
27. Sauce, vegetables (djabadji) ---------------------------35
28. Sauce, dry okra ---35
29. Dish, leafy vegetables (babenda)----------------------37
30. Pieces of meat --37
31. Fish ---39

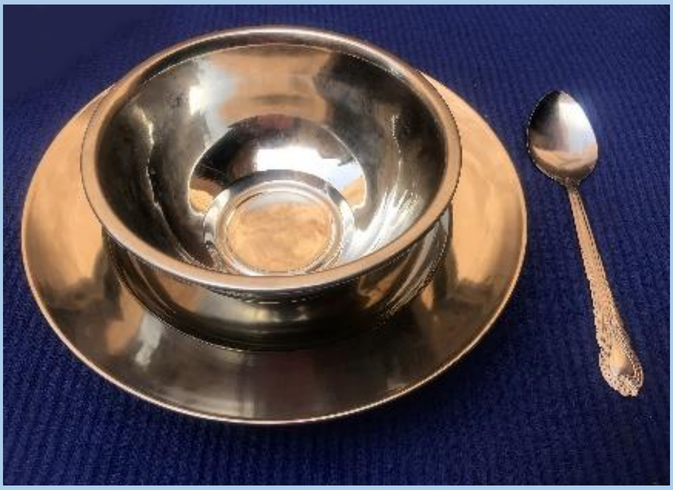

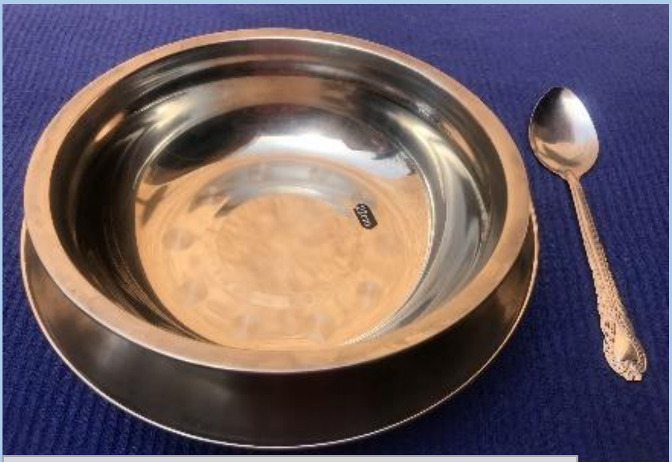

Bowl used for millet porridge

21 WCA – Fermented millet porridge

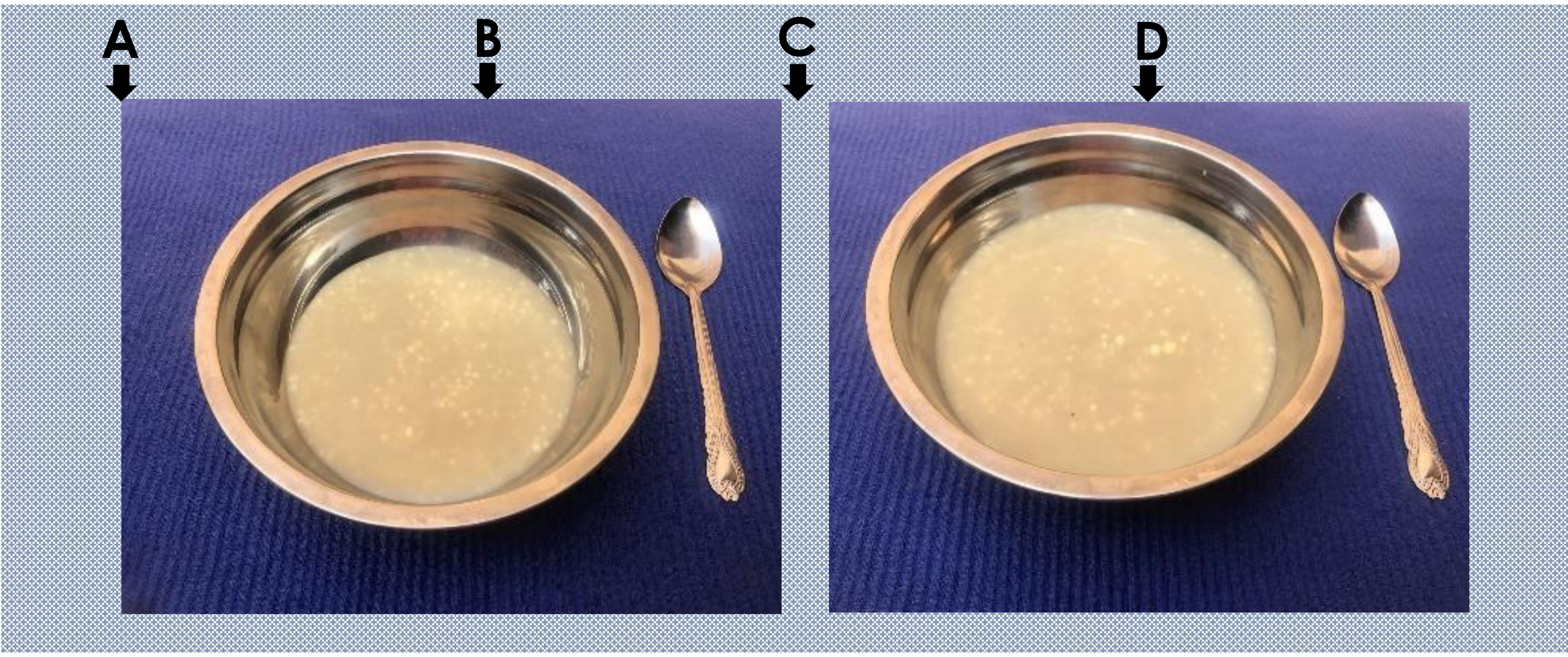

22 WCA– Stiff corn porridge

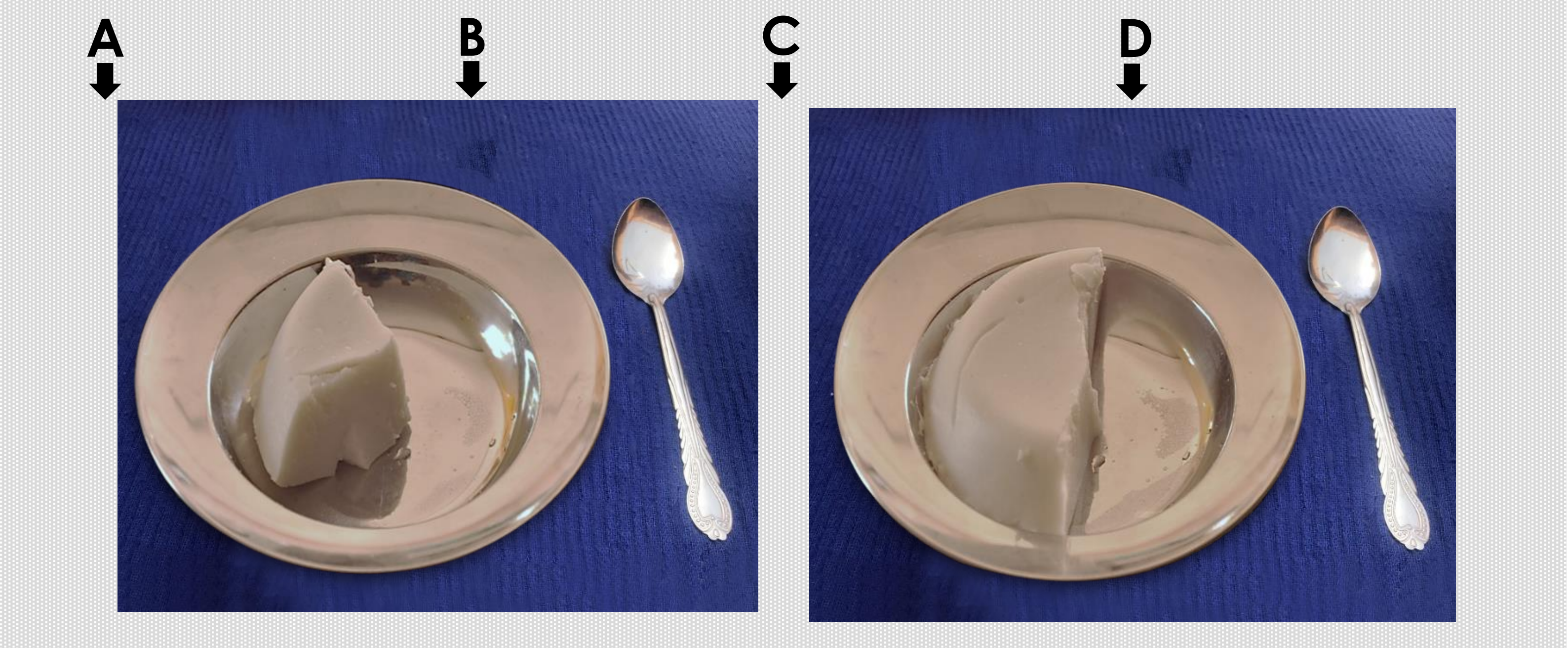

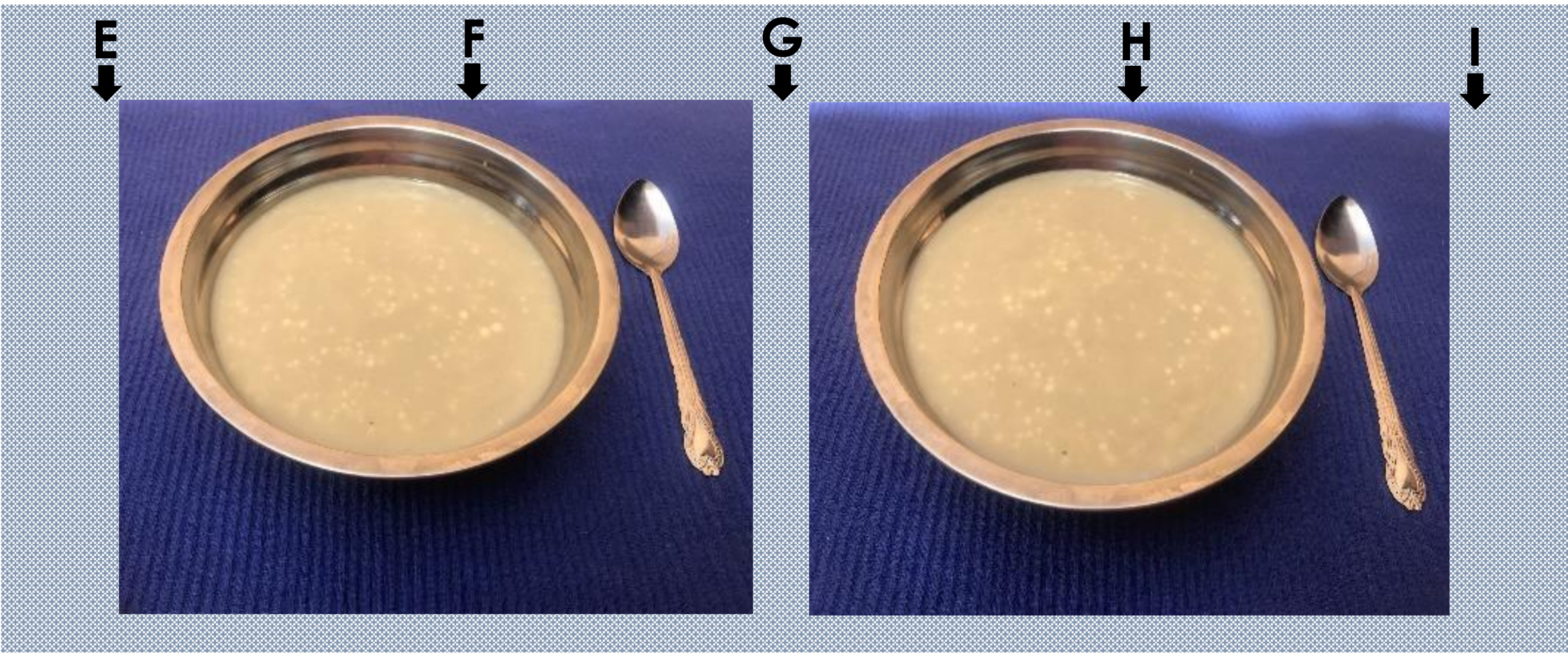
E
F
G
H
I

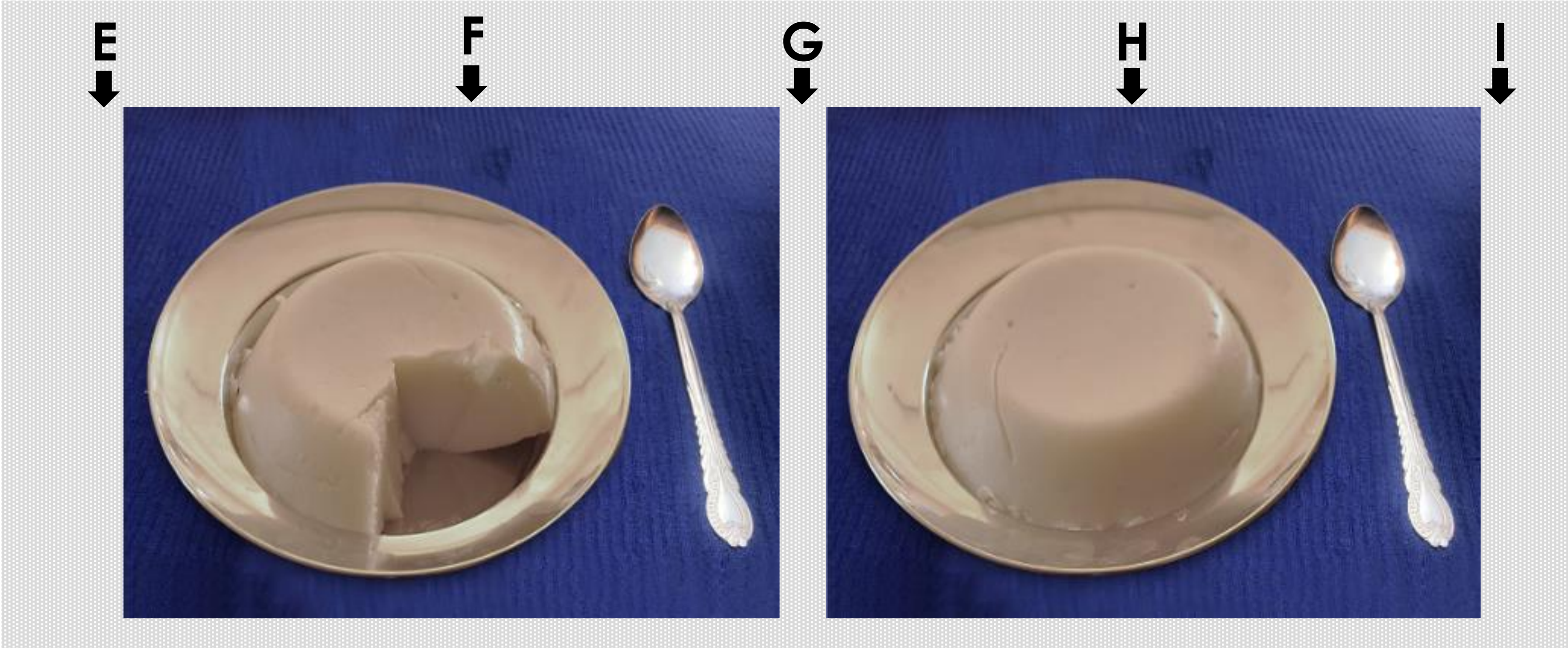
E
F
G
H
I

23 WCA – Rice, boiled

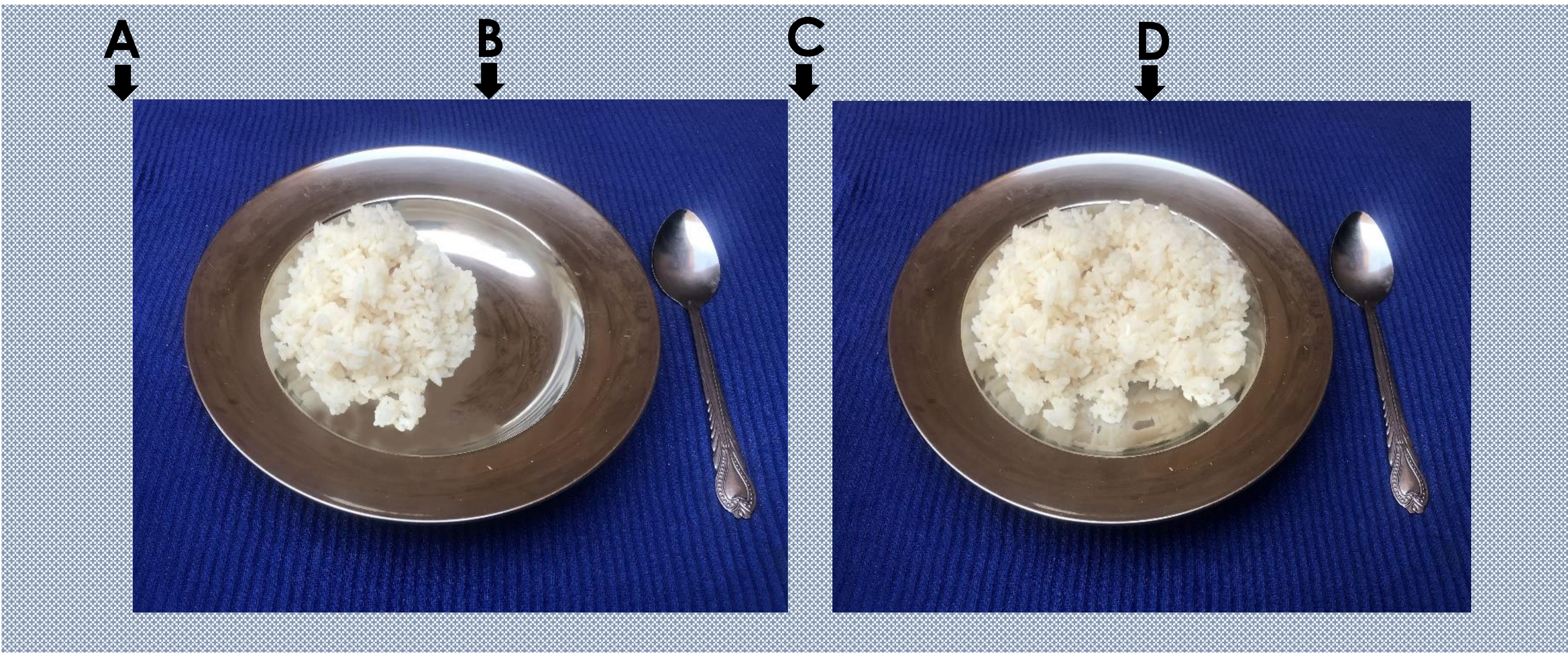

24 WCA – Cowpea with rice

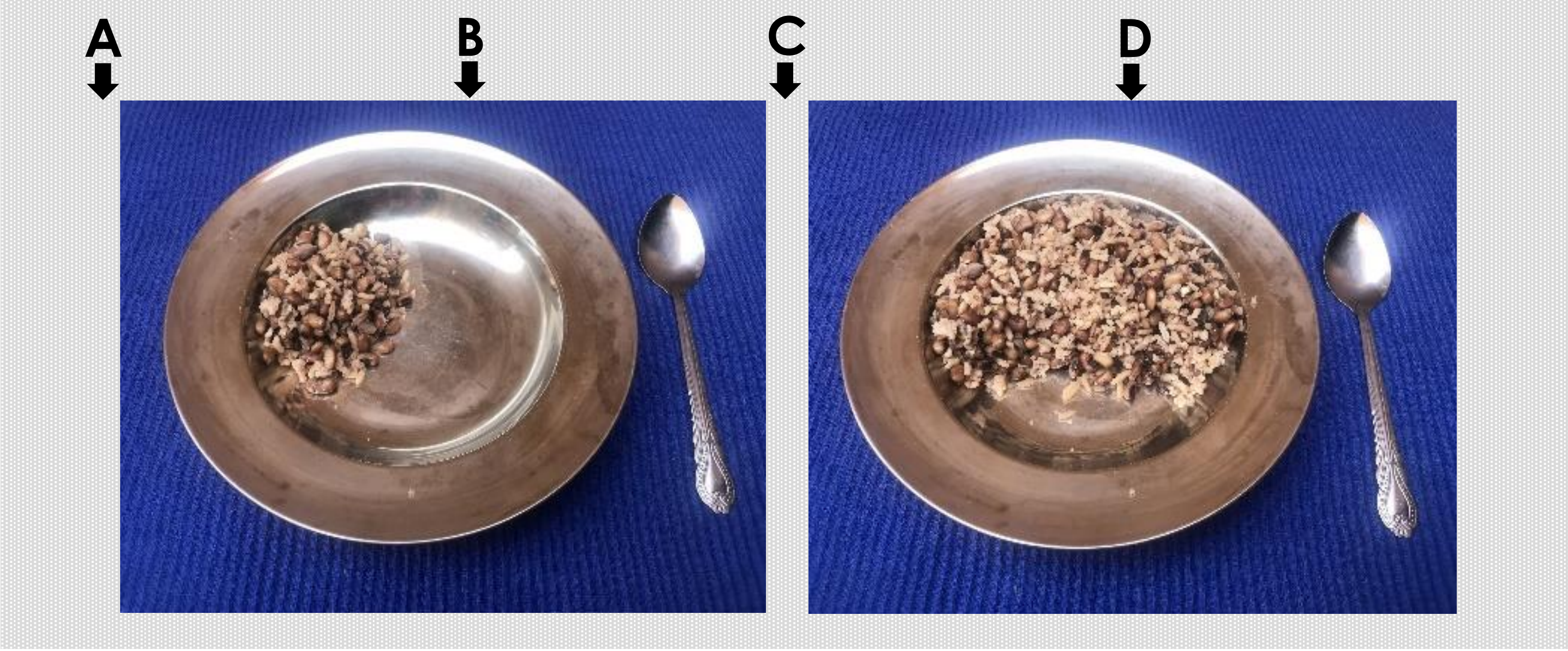

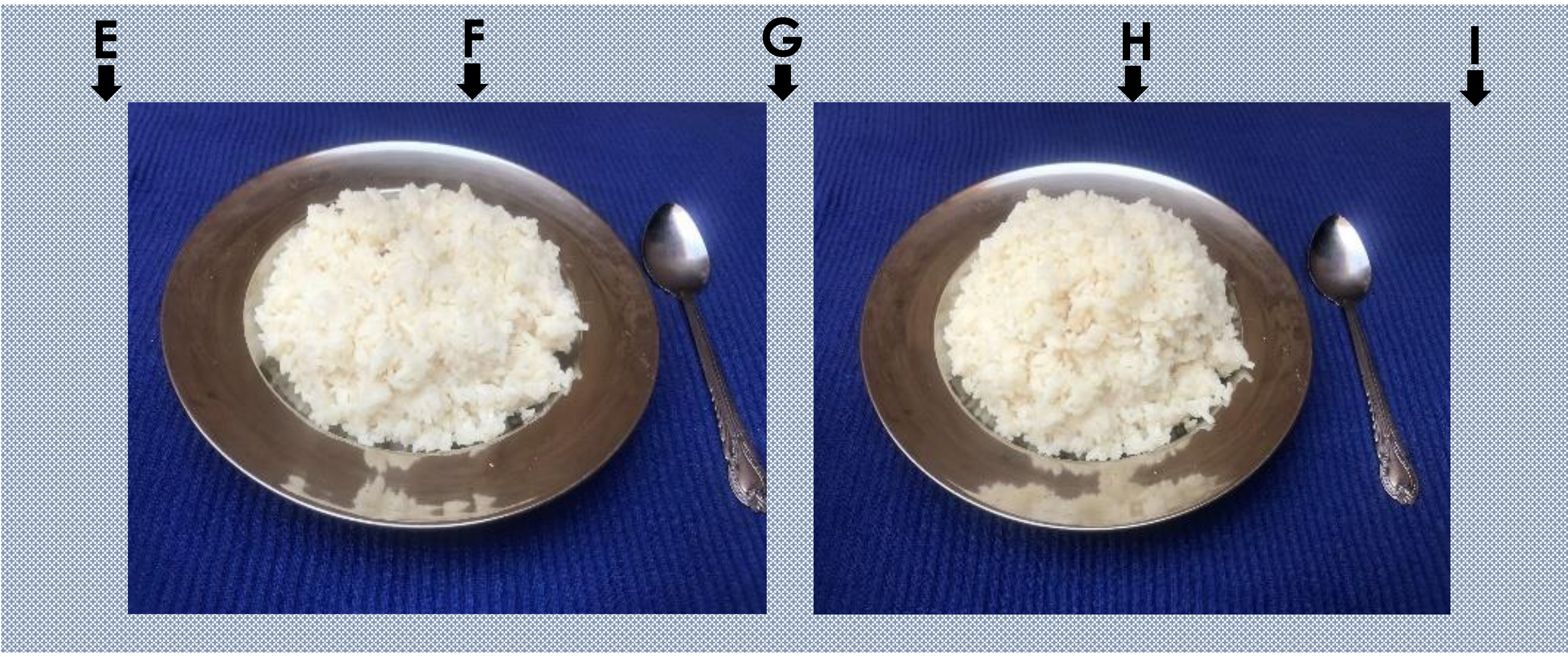
E
F
G
H
I

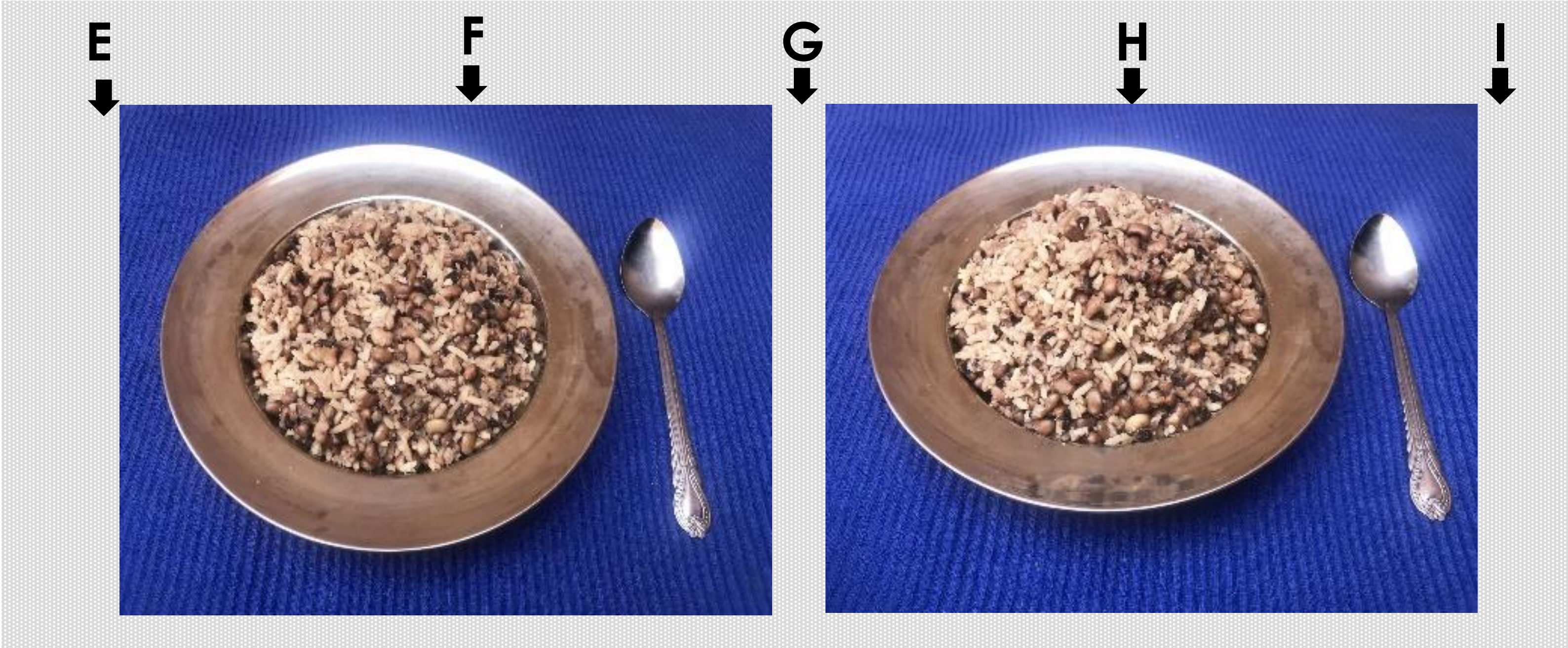
E
F
G
H
I

25 WCA – Spaghetti

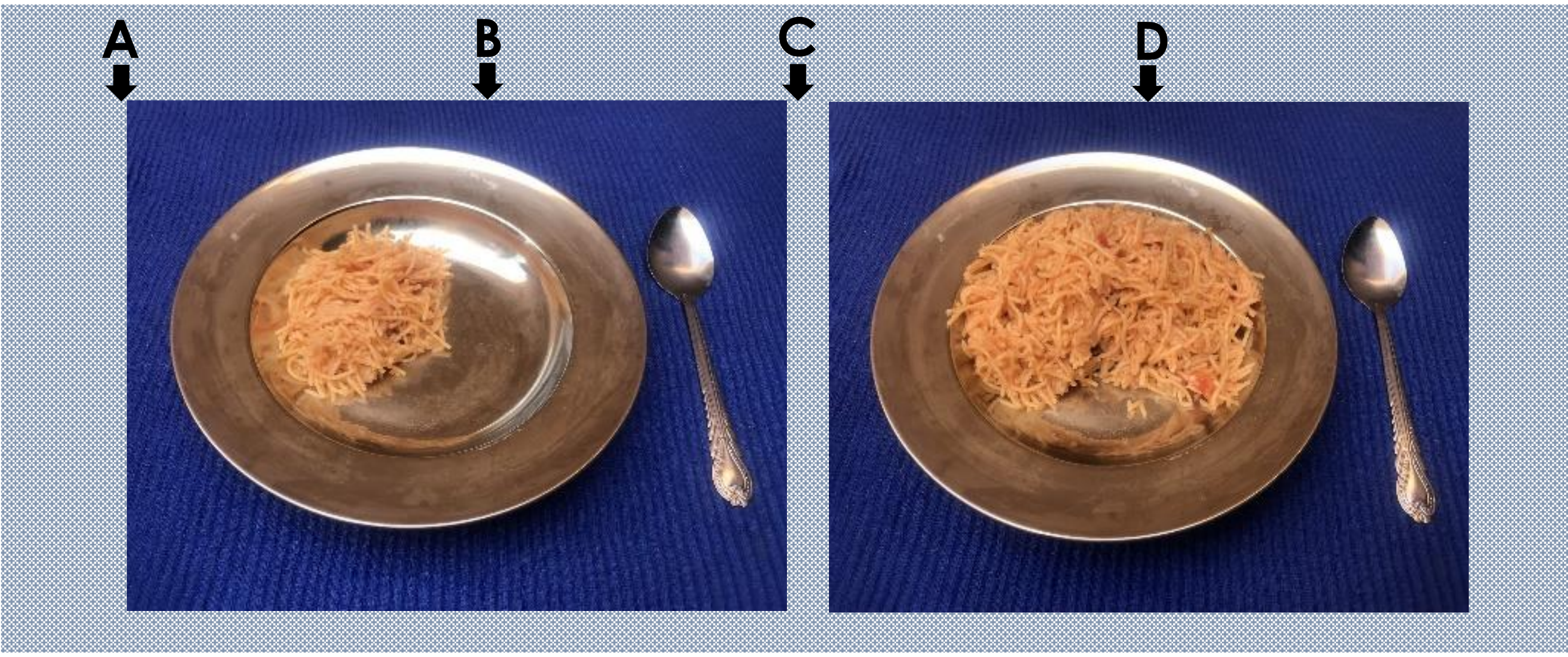

26 WCA – Sauce, groundnut paste

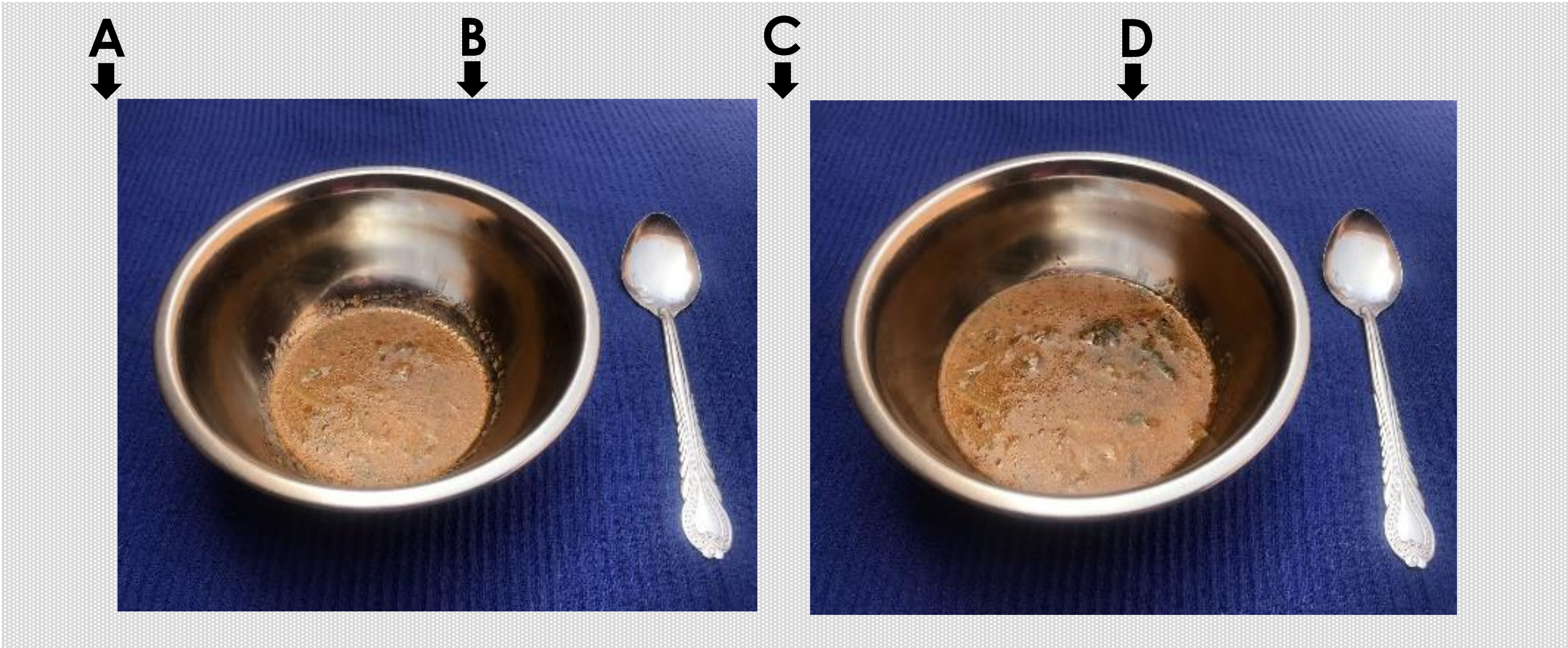

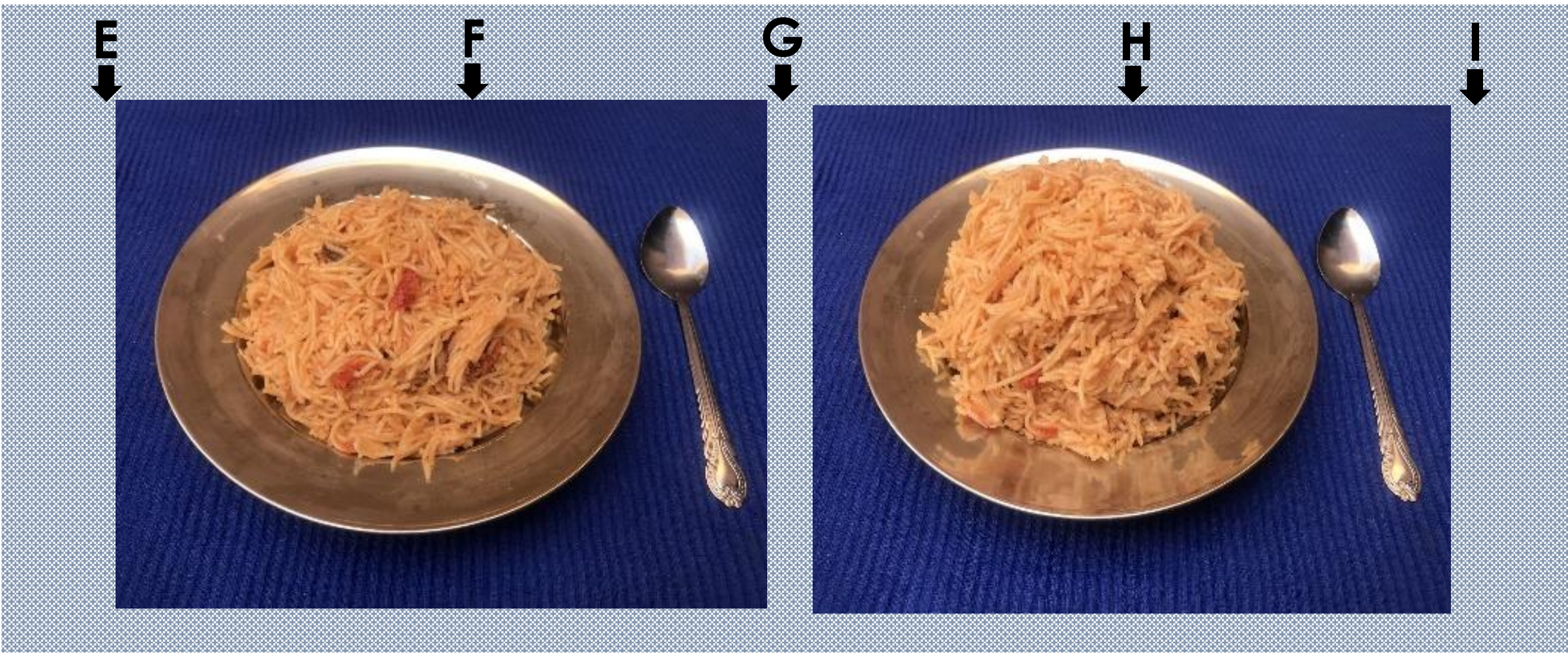
E
F
G
H
I

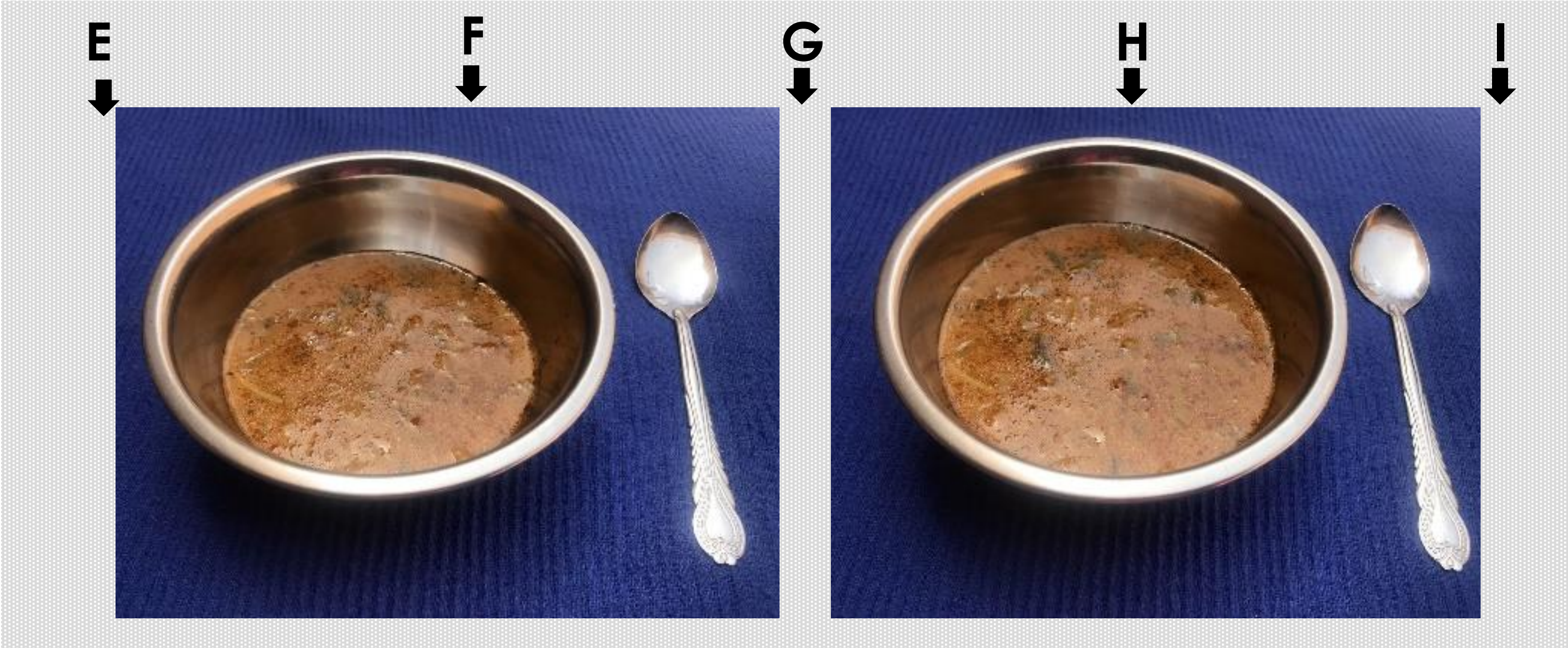
E
F
G
H
I

27 WCA - Sauce, vegetables (djabadji)

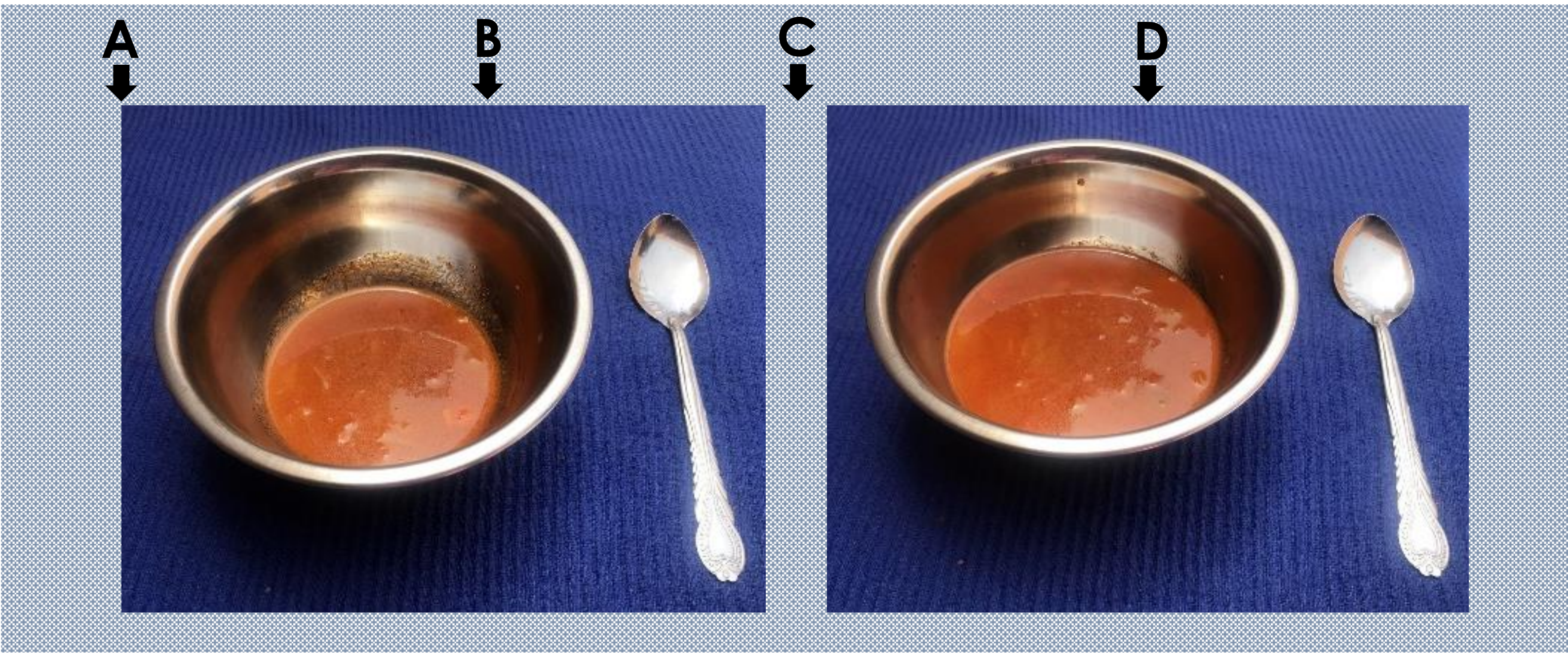

28 WCA – Sauce, dry okra

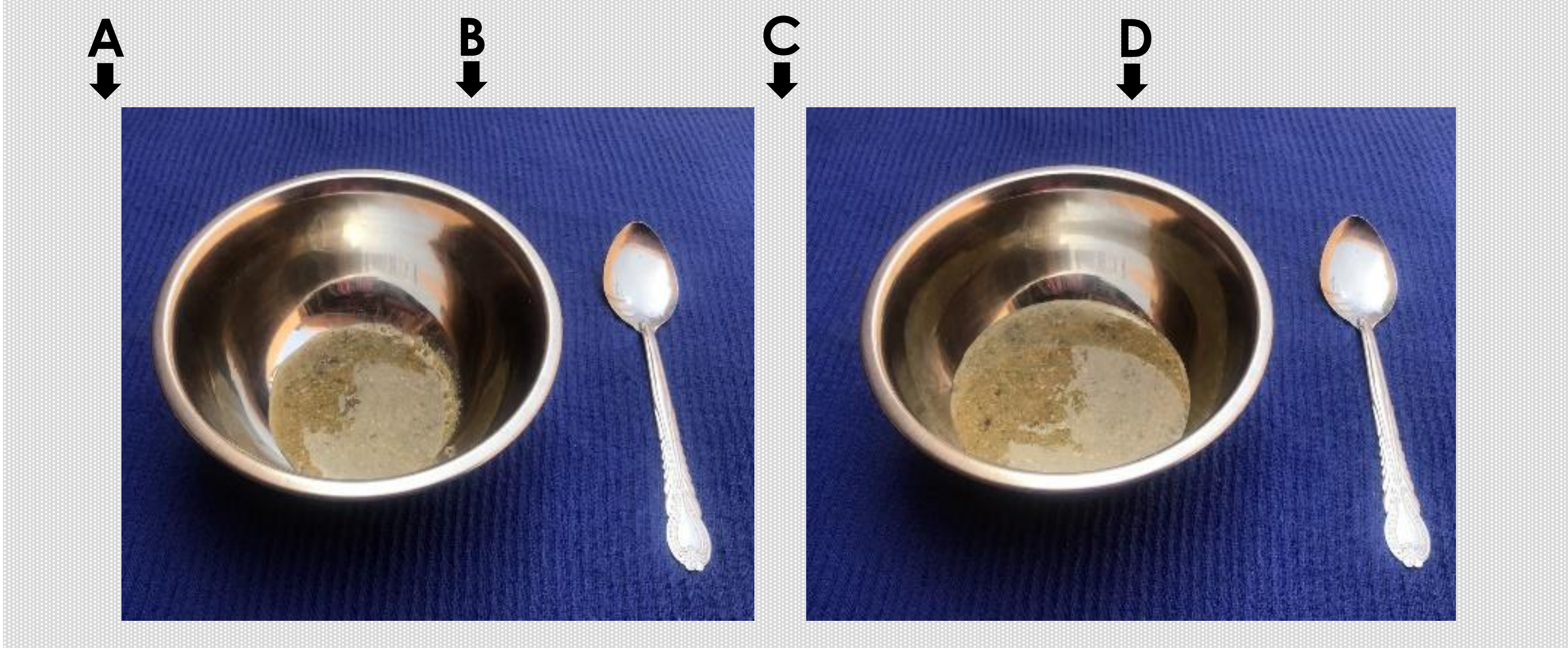

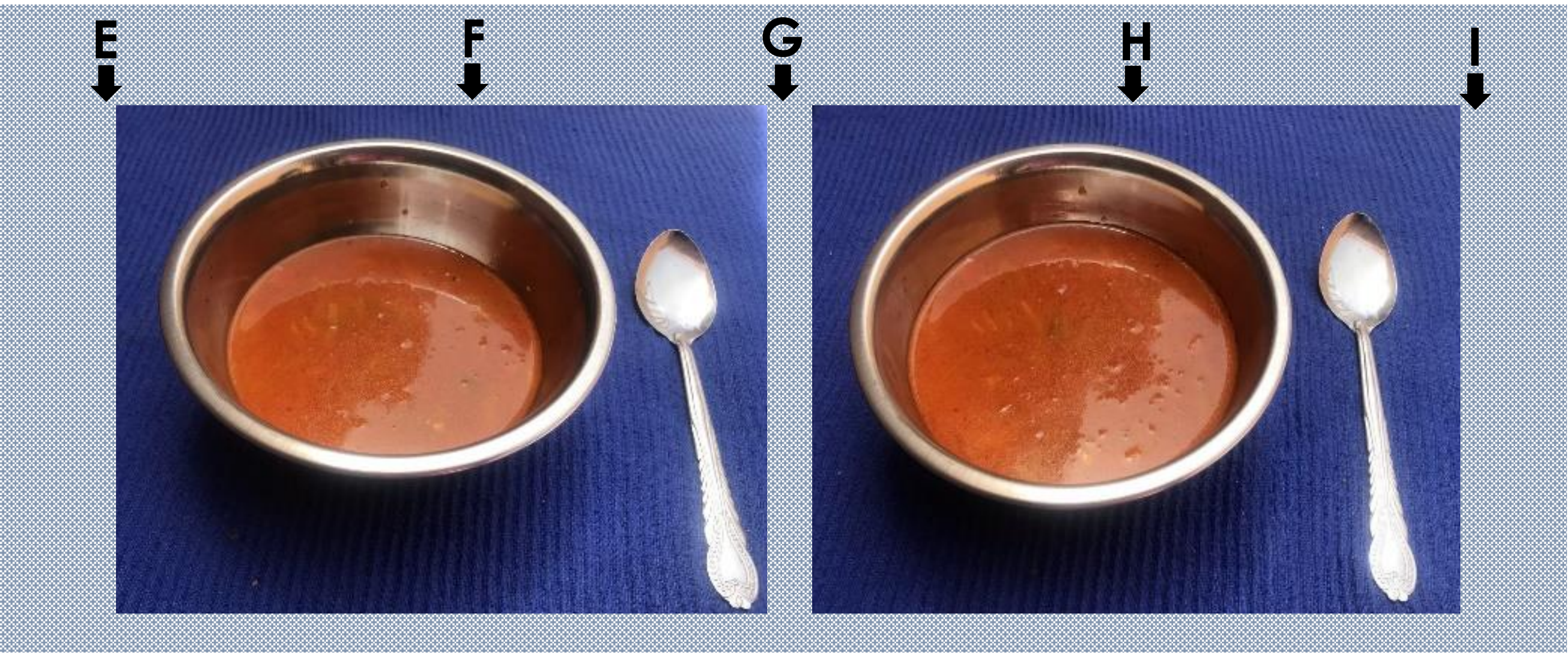
E
F
G
H
I

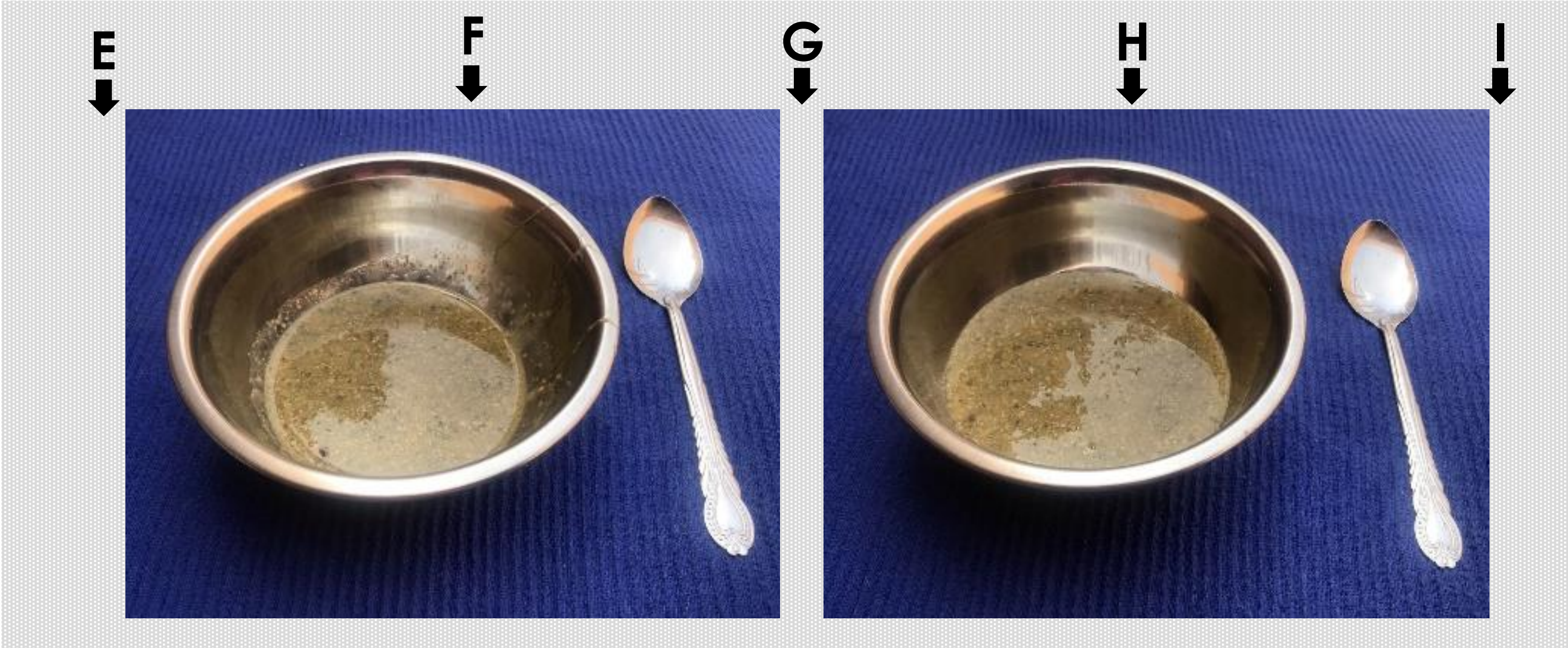
E
F
G
H
I

29 WCA - Dish, leafy vegetables

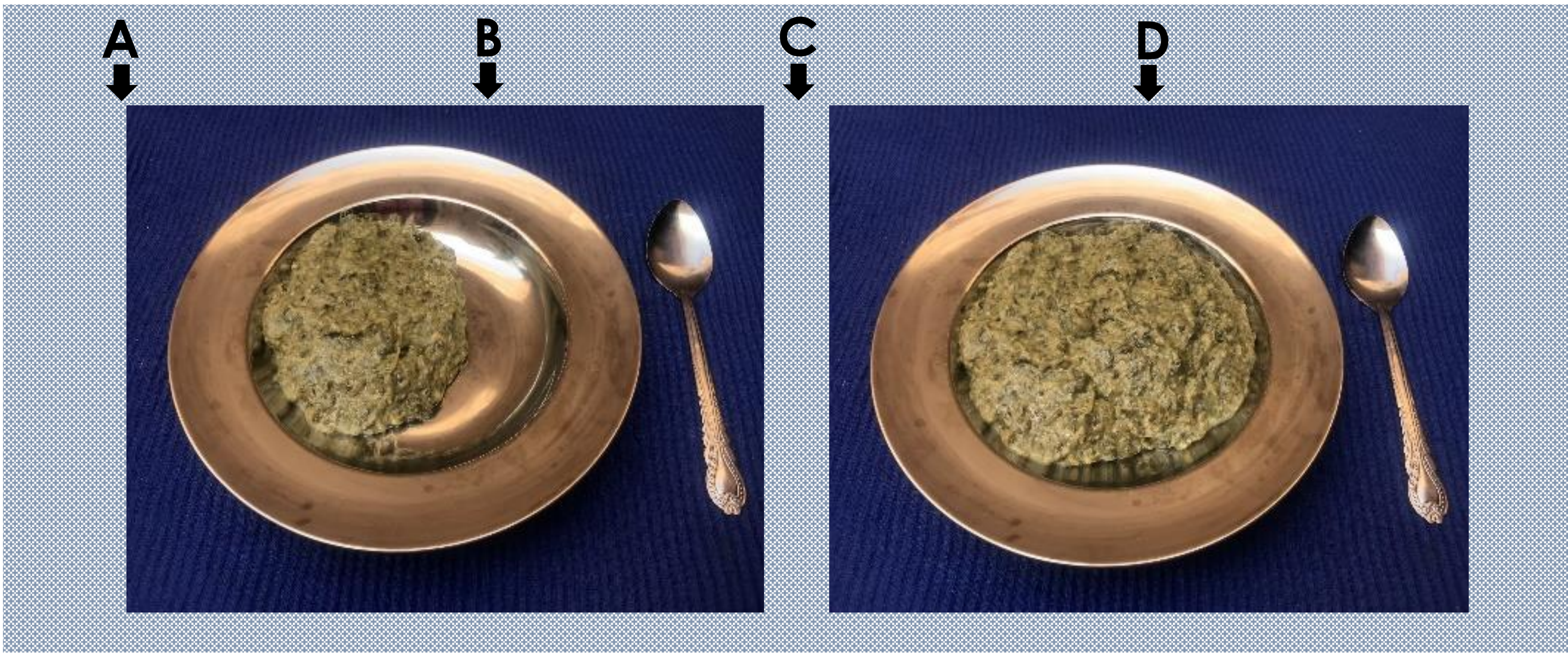

30 WCA – Pieces of meat

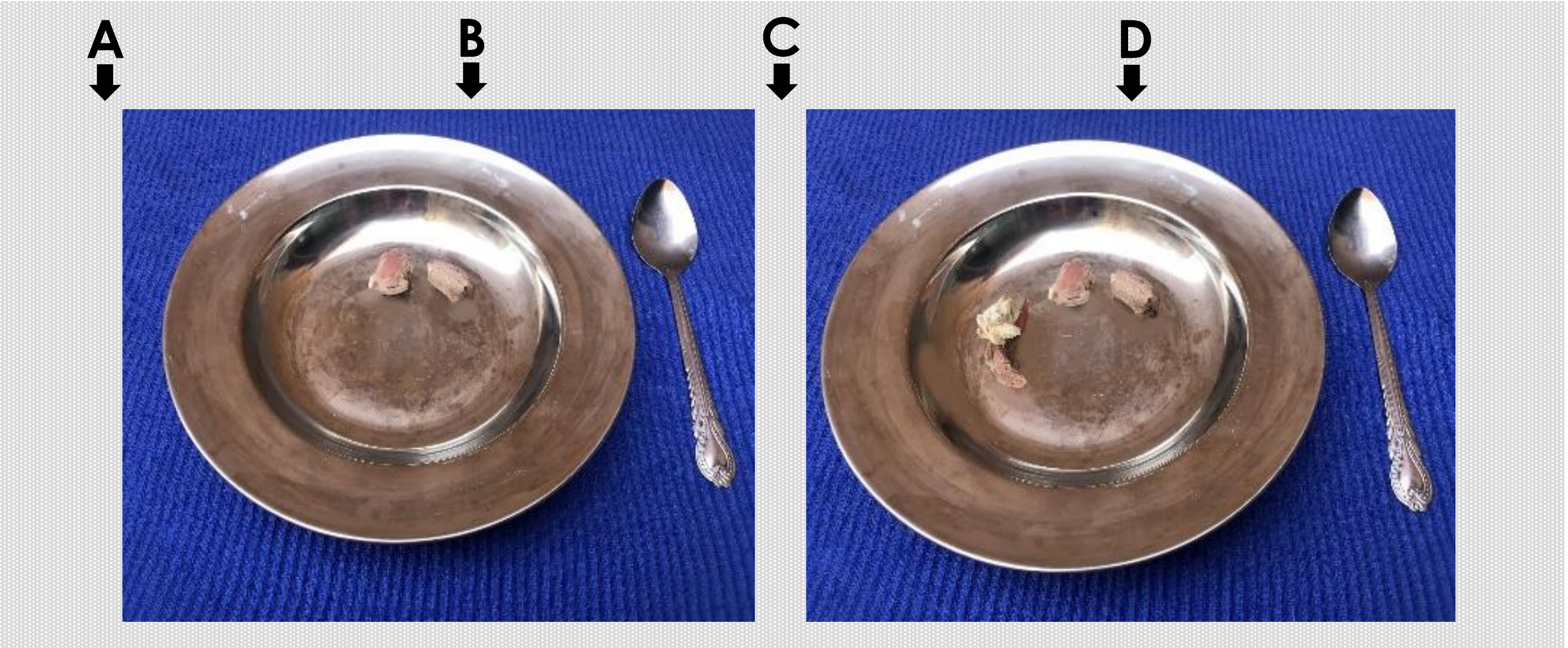

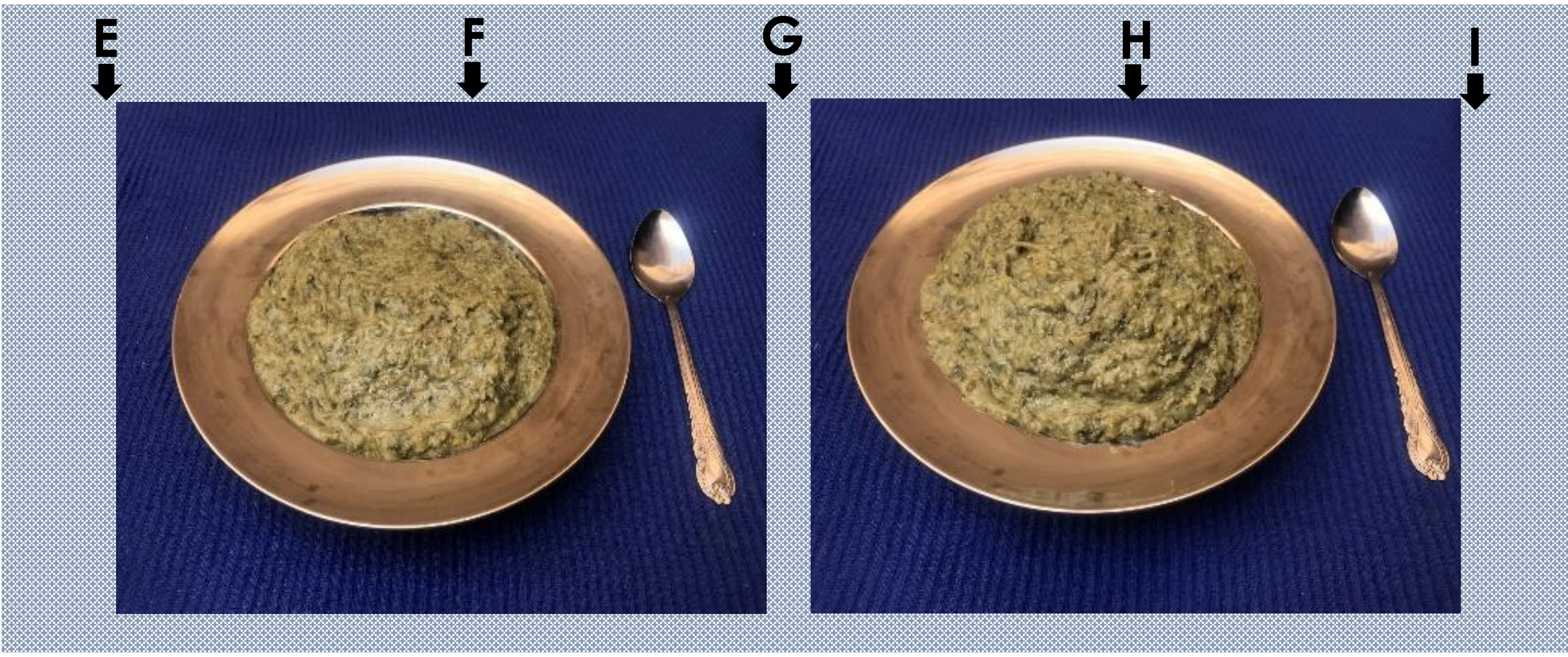
E
F
G
H
I

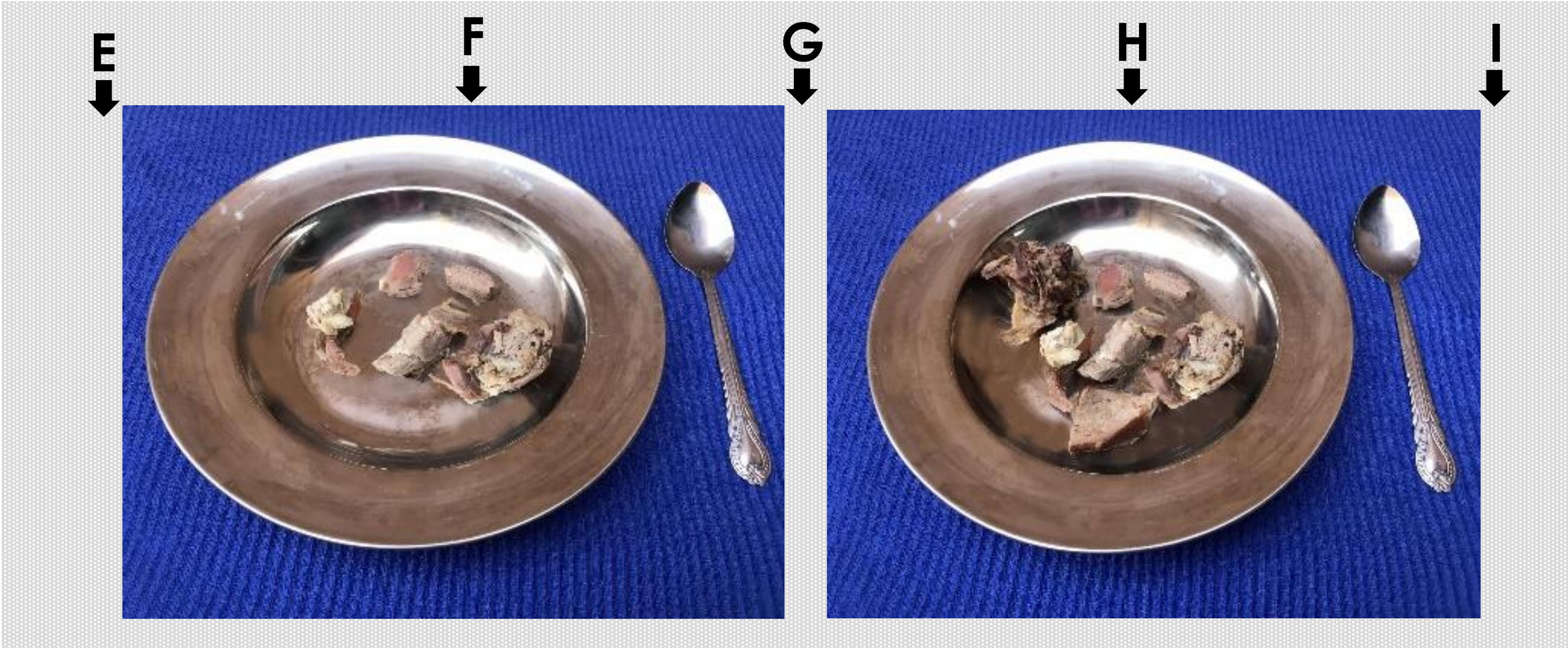
E
F
G
H
I

31 WCA – Fish

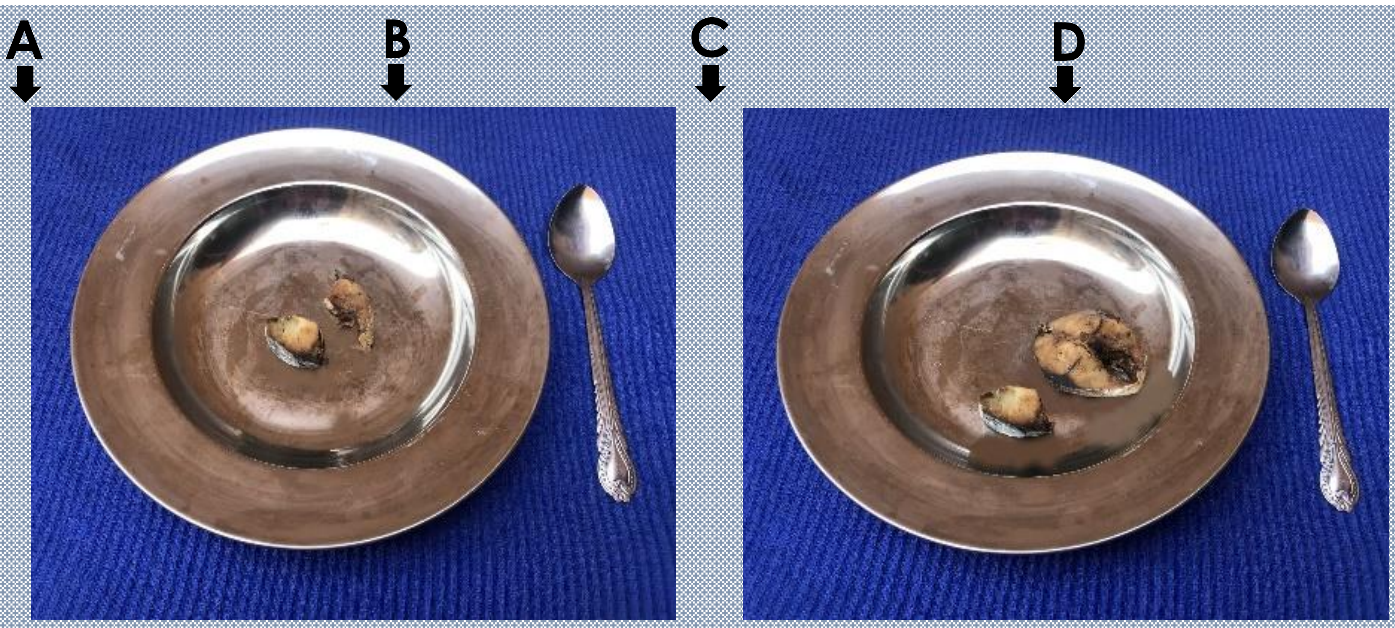

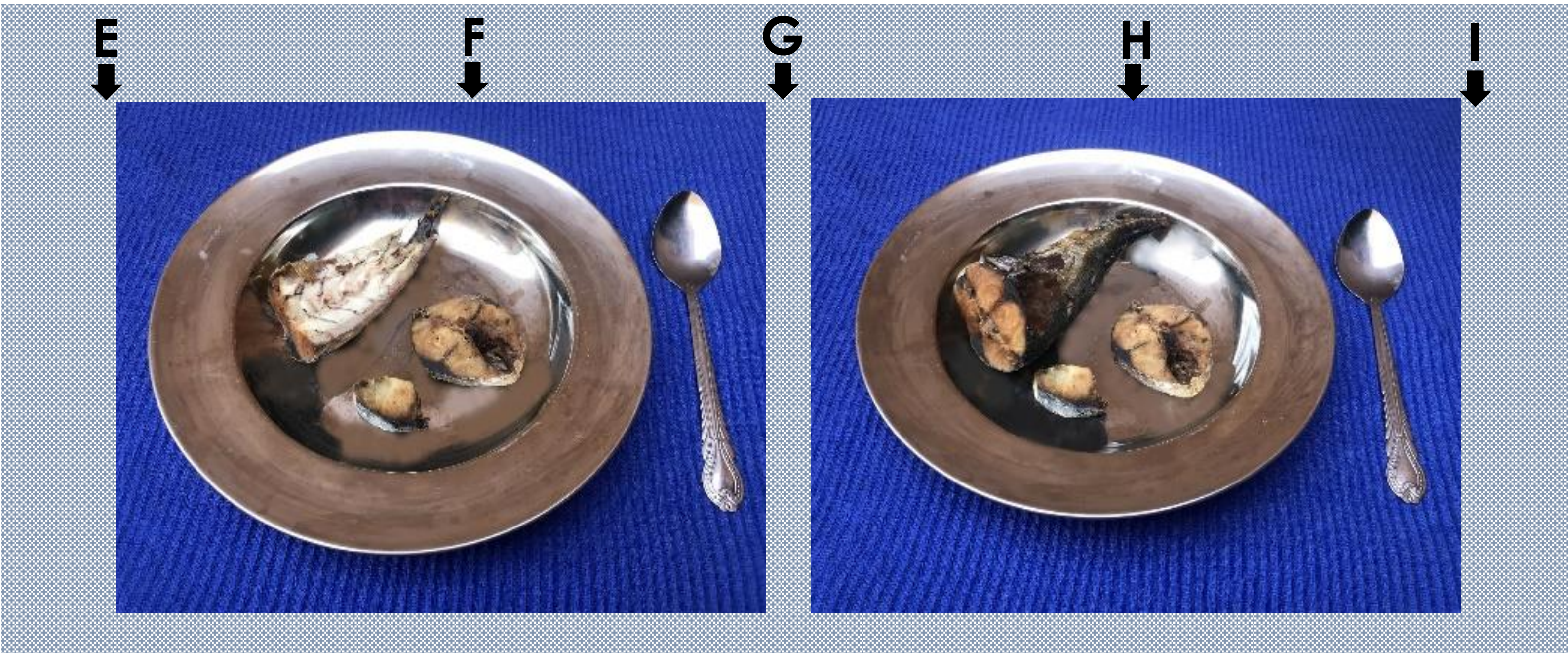
E
F
G
H
I

Portion weights

	For 6-11-months-children								
	Portion A (g) (B/2)	Portion B (g) (mean - 1.5 SD)	Portion C (g) (B+D) /2	Portion D (g) (mean - 0.5 SD)	Portion E (g) (mean)	Portion F (g) (mean + 0.5 SD)	Portion G (g) (F+H) /2	Portion H (g) (mean + 1.5 SD)	Portion I (g) (H+ H/2)
Fermented millet porridge	17	35	65	95	125	155	185	215	323
Stiff corn porridge	15	30	53	77	100	124	148	171	257
Rice, boiled	4	8	33	58	82	107	131	156	234
Cowpea with rice	11	21	39	58	76	94	113	131	197
Spaghetti	24	48	66	84	103	121	139	158	237
Sauce, groundnut paste	4	7	24	41	59	76	93	110	165
Sauce, vegetables (djabadji)	7	14	33	51	70	89	107	126	189
Sauce, dry okra	16	31	44	57	70	82	95	108	162
Dish, leafy vegetables (babenda)	25	49	70	91	112	133	154	175	262
	**For 12-23-months-children**								
	Portion A (g) (B/2)	Portion B (g) (mean - 1.5 SD)	Portion C (g) (B+D) /2	Portion D (g) (mean - 0.5 SD)	Portion E (g) (mean)	Portion F (g) (mean + 0.5 SD)	Portion G (g) (F+H) /2	Portion H (g) (mean + 1.5 SD)	Portion I (g) (H+ H/2)
Fermented millet porridge	17	**34**	91	148	194	239	285	330	495
Stiff corn porridge	15	29	71	113	155	196	238	280	419
Rice, boiled	17	35	60	86	111	136	162	187	280
Cowpea with rice	22	**44**	71	99	120	142	163	185	278
Spaghetti	32	65	85	106	126	146	167	187	281
Sauce, groundnut paste	14	27	50	72	94	117	139	161	242
Sauce, vegetables (djabadji)	16	31	53	75	97	119	141	163	244
Sauce, dry okra	11	**21**	41	61	75	88	101	115	172
Dish, leafy vegetables (babenda)	29	59	106	154	202	249	297	345	517
Pieces of meat	3	6	7	9	11	12	14	16	24
Fish	4	**8**	10	12	17	21	28	**34**	51

	For women of chilbearing age (WCA)								
	Portion A (g) (B/2)	Portion B (g) (mean - 1.5 SD)	Portion C (g) (B+D) /2	Portion D (g) (mean - 0.5 SD)	Portion E (g) (mean)	Portion F (g) (mean + 0.5 SD)	Portion G (g) (F+H) /2	Portion H (g) (mean + 1.5 SD)	Portion I (g) (H+ H/2)
Fermented millet porridge	142	285	409	534	659	784	877	**971**	1456
Stiff corn porridge	76	151	237	322	407	492	578	663	994
Rice, boiled	92	184	256	327	399	470	542	613	920
Cowpea with rice	30	60	139	218	297	376	455	534	801
Spaghetti	39	**78**	180	281	407	533	660	786	1179
Sauce, groundnut paste	33	66	114	162	211	259	307	356	533
Sauce, vegetables (djabadji)	41	83	132	182	232	281	331	381	571
Sauce, dry okra	23	46	83	119	155	192	228	265	397
Dish, leafy vegetables (babenda)	107	213	317	422	526	630	734	838	1257
Pieces of meat	5	**10**	14	17	33	48	64	79	118
Fish	8	**16**	28	40	64	88	112	136	204

In bold: adjusted portions depending on whether they were considered too large or too small compared to the average portion
